# Cross-species oncogenomics offers insight into human muscle-invasive bladder cancer

**DOI:** 10.1186/s13059-023-03026-4

**Published:** 2023-08-28

**Authors:** Kim Wong, Federico Abascal, Latasha Ludwig, Heike Aupperle-Lellbach, Julia Grassinger, Colin W. Wright, Simon J. Allison, Emma Pinder, Roger M. Phillips, Laura P. Romero, Arnon Gal, Patrick J. Roady, Isabel Pires, Franco Guscetti, John S. Munday, Maria C. Peleteiro, Carlos A. Pinto, Tânia Carvalho, João Cota, Elizabeth C. Du Plessis, Fernando Constantino-Casas, Stephanie Plog, Lars Moe, Simone de Brot, Ingrid Bemelmans, Renée Laufer Amorim, Smitha R. Georgy, Justina Prada, Jorge del Pozo, Marianne Heimann, Louisiane de Carvalho Nunes, Outi Simola, Paolo Pazzi, Johan Steyl, Rodrigo Ubukata, Peter Vajdovich, Simon L. Priestnall, Alejandro Suárez-Bonnet, Franco Roperto, Francesca Millanta, Chiara Palmieri, Ana L. Ortiz, Claudio S. L. Barros, Aldo Gava, Minna E. Söderström, Marie O’Donnell, Robert Klopfleisch, Andrea Manrique-Rincón, Inigo Martincorena, Ingrid Ferreira, Mark J. Arends, Geoffrey A. Wood, David J. Adams, Louise van der Weyden

**Affiliations:** 1 Experimental Cancer Genetics, Wellcome Sanger Institute, Wellcome Genome Campus, Hinxton, Cambridge, CB10 1SA UK; 2 Department of Pathobiology, University of Guelph, Guelph, ON Canada; 3 Laboklin GmbH & Co. KG, Bad Kissingen, Germany and Institute of Pathology, Department Comparative Experimental Pathology, School of Medicine, Technical University of Munich, Munich, Germany; 4 School of Pharmacy and Medical Sciences, University of Bradford, West Yorkshire, UK; 5 Department of Pharmacy, University of Huddersfield, Queensgate, Huddersfield, UK; 6 Departmento de Patología, Facultad de Medicina Veterinaria Y Zootecnia, Universidad Nacional Autónoma de México (UNAM), CDMX, Mexico City, México; 7 Department of Veterinary Clinical Medicine, College of Veterinary Medicine, University of Illinois at Urbana-Champaign, Urbana, IL USA; 8 Department of Veterinary Science, CECAV-Veterinary and Animal Research Center, University of Trás-Os-Montes and Alto Douro, Vila Real, Portugal; 9 Institute of Veterinary Pathology, University of Zurich, Zurich, Switzerland; 10 School of Veterinary Science, Massey University, Palmerston North, New Zealand; 11 Faculty of Veterinary Medicine, Centre for Interdisciplinary Research in Animal Health (CIISA), University of Lisbon, Lisbon, Portugal; 12 Champalimaud Foundation, Lisbon, Portugal; 13 Division of Pathology, IDEXX Laboratories, Kyalami, South Africa; 14 Department of Veterinary Medicine, University of Cambridge, Cambridge, UK; 15 The Veterinary Pathology Group (VPG), Bristol, UK; 16 Department of Companion Animal Clinical Sciences, Norwegian University of Life Sciences, Ås, Norway; 17 Institute of Animal Pathology, COMPATH, University of Bern, Bern, Switzerland; 18 Cerba Vet, Île-de-France, 91300 Massy, France; 19 Veterinary Clinic Department, School of Veterinary Medicine and Animal Science, São Paulo State University, Botucatu, Brazil; 20 Department of Anatomic Pathology, Faculty of Veterinary and Agricultural Sciences, University of Melbourne, Victoria, Australia; 21 Royal Dick School of Veterinary Sciences, University of Edinburgh, Roslin, Scotland, UK; 22 Anapet, Montigny-Le-Tilleul, Belgium; 23 Departamento de Medicina Veterinária, Universidade Federal Do Espírito Santo, Alegre, ES Brazil; 24 Finnish Food Authority, Helsinki, Finland; 25 Department of Companion Animal Clinical Studies, Faculty of Veterinary Science, University of Pretoria, Pretoria, South Africa; 26 Department of Paraclinical Sciences, Faculty of Veterinary Science, University of Pretoria, Pretoria, South Africa; 27 E+ Especialidades Veterinárias - Veterinary Oncology, São Paulo, Brazil; 28 Department of Clinical Pathology and Oncology, University of Veterinary Medicine Budapest, Budapest, Hungary; 29 Department of Pathobiology and Population Sciences, The Royal Veterinary College, Hatfield, UK; 30 Dipartimento Di Biologia, Università Degli Studi Di Napoli Federico II, Napoli, Italy; 31 Department of Veterinary Sciences, University of Pisa, Pisa, Italy; 32 School of Veterinary Science, The University of Queensland, Brisbane, QLD Australia; 33 School of Veterinary Medicine and Science, University of Nottingham, Nottingham, UK; 34 Faculdade de Medicina Veterinária E Zootecnia, Universidade Federal de Mato Grosso Do Sul, Campo Grande, MS Brazil; 35 Pathology Laboratory of the Centro de Ciencias Agro-Veterinarias, Universidade Do Estado de Santa Catarina, Lages, SC Brazil; 36 Department of Veterinary Biosciences, Faculty of Veterinary Medicine, University of Helsinki, Helsinki, Finland; 37 Department of Pathology, Western General Hospital, Edinburgh, Scotland, UK; 38 Institute of Veterinary Pathology, Freie Universität Berlin, Berlin, Germany; 39 University of Edinburgh Division of Pathology, Cancer Research UK Edinburgh Cancer Centre, Institute of Genetics & Cancer, Edinburgh, Scotland, UK

**Keywords:** Canine, Feline, Bovine, Urinary bladder, Cancer, Mutational signature, Bracken, Ptaquiloside, *Pteridium aquilinum*, Cross-species comparison

## Abstract

**Background:**

In humans, muscle-invasive bladder cancer (MIBC) is highly aggressive and associated with a poor prognosis. With a high mutation load and large number of altered genes, strategies to delineate key driver events are necessary. Dogs and cats develop urothelial carcinoma (UC) with histological and clinical similarities to human MIBC. Cattle that graze on bracken fern also develop UC, associated with exposure to the carcinogen ptaquiloside. These species may represent relevant animal models of spontaneous and carcinogen-induced UC that can provide insight into human MIBC.

**Results:**

Whole-exome sequencing of domestic canine (*n* = 87) and feline (*n* = 23) UC, and comparative analysis with human MIBC reveals a lower mutation rate in animal cases and the absence of APOBEC mutational signatures. A convergence of driver genes (*ARID1A, KDM6A, TP53*, *FAT1*, and *NRAS*) is discovered, along with common focally amplified and deleted genes involved in regulation of the cell cycle and chromatin remodelling. We identify mismatch repair deficiency in a subset of canine and feline UCs with biallelic inactivation of *MSH2*. Bovine UC (*n* = 8) is distinctly different; we identify novel mutational signatures which are recapitulated in vitro in human urinary bladder UC cells treated with bracken fern extracts or purified ptaquiloside.

**Conclusion:**

Canine and feline urinary bladder UC represent relevant models of MIBC in humans, and cross-species analysis can identify evolutionarily conserved driver genes. We characterize mutational signatures in bovine UC associated with bracken fern and ptaquiloside exposure, a human-linked cancer exposure. Our work demonstrates the relevance of cross-species comparative analysis in understanding both human and animal UC.

**Supplementary Information:**

The online version contains supplementary material available at 10.1186/s13059-023-03026-4.

## Background

The incidence of urinary bladder cancer (UBC) in humans varies significantly between countries, largely due to the differential exposure to established risk factors such as smoking, arsenic in drinking water, and chemical carcinogens (such as aromatic amines) from occupational exposures, and endemic chronic urinary infections with *Schistosoma* [1]. In the USA, UBC remains the fourth most common cancer in men and is predicted to account for 4% of all cancer deaths in 2022 [2]. In the USA, UK, and Europe, > 90% of UBC cases are urothelial carcinoma (UC; formerly known as transitional cell carcinoma, TCC). At the time of diagnosis, approximately 25% of UCs are muscle-invasive (MIBC); highly aggressive tumors that are considered high risk due to their propensity for rapid growth and metastasis. Platinum-based neoadjuvant chemotherapy followed by radical cystectomy with lymph node dissection is recommended. However, this is not always possible and the 5-year survival rate is only 38% if the tumor has spread to the surrounding tissues or regional lymph nodes (6% if there are distant metastases) [2]. Despite progress being made with treatment options, such as immune checkpoint inhibitors which are currently being offered in clinical trials [3–5], prognosis remains poor [6, 7].

While both chemical carcinogen-induced and genetically engineered mouse models of MIBC [8, 9] share some histological and molecular similarities to human MIBC, spontaneously occurring urinary bladder UC in pet dogs and cats may offer distinct advantages as models of MIBC. Dogs and cats represent a genetically heterogeneous population sharing the same co-morbidities and environment as humans [10–13], and UC is their most common type of UBC. Additionally, most urinary bladder UCs in dogs and cats are also high-grade and invasive, with a high propensity for both recurrence and metastasis [14]. In some countries, there is a high prevalence of UBC in cattle associated with chronic ingestion of bracken fern (BF; *Pteridium aquilinum* (L.) Kuhn) [15], which contains several toxic components including the carcinogen ptaquiloside (PT) [16]. UC is the most common type of urinary bladder wall lesion in these cattle [17] and, thus, cattle that have consumed BF may represent an animal model of carcinogen-induced UC. Further relevance for this model comes from the fact that humans can be exposed to PT from spore inhalation, consumption of milk from BF-fed cattle [18, 19], and the groundwater from regions where BF grows [20]. Additionally, BF used by humans either as a food or traditional medicine, has recently been shown to contain detectable levels of PT [21].

Here, we have sequenced the exomes of canine, feline, and bovine UC and matched normal tissue and profiled somatic mutations and copy number alterations. Sequencing of matched normal tissue also allowed us to search for germline variants that may predispose to UC. We performed a comparative cross-species analysis with human MIBC and identified important differences between the animal models and human UC, while the commonalities between the species enabled us to refine the list of candidate driver genes previously identified in human MIBC.

## Results

### Whole-exome sequencing of urinary bladder UC in dogs, cats, and cows

We performed whole-exome sequencing (WES) of tumor and matched normal tissue on the largest cohort of canine UC tumor-normal matched samples to date (*n* = 87 cases; 29 males and 58 females, representing 36 different pure and mixed breeds), and the first cohort of feline UC (*n* = 23 cases; 14 males and 9 females, representing 6 different breeds) and bovine UC samples (*n* = 8 cases from 7 females that had been grazing in pastures where BF grow; one cow having 2 independent lesions). The samples were collected from multiple institutions (*n* = 25) across different countries (*n* = 17), so as to minimize ascertainment bias and account for geographical differences. A summary of cases from these three species, their geographic location and signalment data is provided in Additional file 1: Table S1. For each cohort, we profiled somatic mutations, somatic copy number alterations (SCNA), germline variants, and mutational signatures and performed a comparative analysis with a previously published cohort of 412 WES human MIBC samples collected from 36 institutions across 6 countries [22]). Examples of UC tumor pathology from canine, feline, bovine, and human UC cases are shown in Additional file 2: Fig. S1.

### Cross-species comparison of frequently mutated genes

To gain an overview of the somatic mutational landscape in the exomes of UC across species, we identified somatic single-nucleotide variants (SNVs), multi-nucleotide variants (MNVs), and small insertions/deletions (indels), present in canine, feline, and bovine UC cases (Additional File 3: Table S2, Additional File 4: Table S3 and Additional File 5: Table S4, respectively) and compared these catalogs of mutations to those found in human UC, revealing both notable similarities and differences between the species. With a median of 5.5 mutations/Mb, human MIBC has one of the highest somatic mutation rates [23], with levels similar to that seen in non-small cell lung cancer and melanoma [24]. Lower somatic mutation rates were seen in canine UC (median 1.0 mutations/Mb; 0.94 SNV or MNV/Mb and 0.074 indels/Mb) and feline UC (median 1.1 mutations/Mb; 0.96 SNV or MNV/Mb and 0.18 indels/Mb); however, bovine UC had significantly higher rates (median 65 mutations/Mb; 51 SNV or MNV/Mb and 11 indels/Mb), reflecting exposure to a strong environmental mutagen. The high mutation rate in the bovine samples convolutes any exome-wide comparisons with the other 3 species, as a large proportion of genes have passenger mutations, and thus, only cross-species comparisons between human, canine, and feline UC were made.

**Fig. 1:**
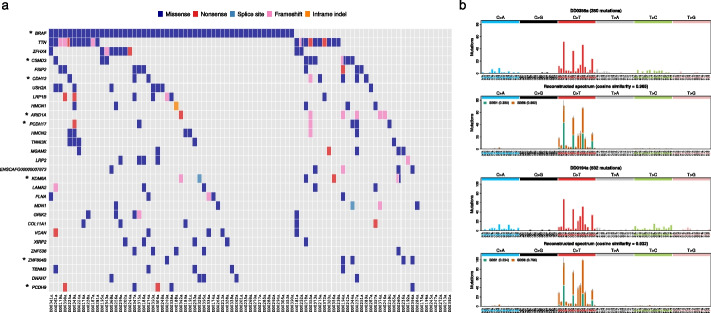
Somatic mutational landscape of canine urinary bladder UC. **a** Somatic mutations in genes mutated in five or more samples. Asterisk indicates significantly mutated gene (SMG); *GPRASP1* is also a SMG but is not shown, as it is mutated in 2 samples. A summary of the canine cases analyzed in this study is provided in Additional File 1: Table S1a and a full list of variants is provided in Additional File 3: Table S2. **b** Single base substitution (SBS) observed mutational spectra and reconstructed spectra for samples DD0194a and DD0355a. Mutational signatures SBS1 and SBS6 were found in both samples as well as SBS21 in sample DD0194a. The reconstructed mutational spectra based on these signatures have cosine similarity > 0.93 when compared to the observed spectra

In contrast to human MIBC, in which *BRAF* is mutated in 2.7% of cases [22], the most commonly mutated gene in canine UC (53/87, 61%) was *BRAF,* as reported previously [25–28] (Fig. 1a). These cases harbored the equivalent of the human BRAF p.V600E hotspot mutation, BRAF p.V588E (relative to the canonical transcript ENSCAFT00000006305.5; also known as p.V595E). There is a strong breed-associated predisposition of urinary bladder UC in dogs; for example, Scottish Terriers and West Highland White Terriers have increased risk, although heritable risk factors have not yet been identified [29, 30]. Within our canine cohort, we found that the somatic mutation of *BRAF* was significantly higher (*p* = 0.0313, chi-squared test) in terrier breeds (19/24) compared to non-terriers (34/63). Using the DISCOVER algorithm [31], we did not find any mutually exclusive or co-occurring mutated genes in the whole cohort or when comparing tumors with mutated and wildtype *BRAF*, which suggests that there are no differences in the driver gene landscape between these tumors. Recent WES of canine urine sediments with no detectable BRAF V595E (as determined by ddPCR) identified 46% of samples harboring short in-frame deletions within exon 12 of *BRAF* (7/28 cases) or exons 2/3 of *MAP2K1* (6/28 cases) [32]. The authors proposed these genetic alterations as alternative MAPK-pathway activating events. In our cohort, we had 2/87 samples with short in-frame deletions in exon 2 of *MAP2K1* (both of which did not carry the BRAF V595E mutation); however, we did not find any samples showing in-frame deletions within exon 12 of *BRAF*, consistent with a previous WES canine bladder UC study [28].

**Fig. 2:**
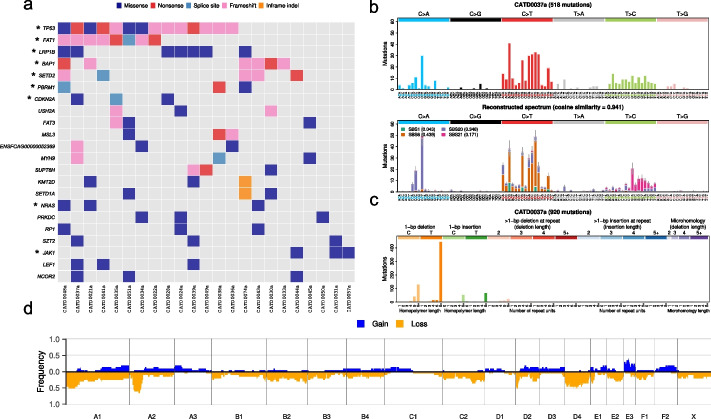
Somatic mutational landscape of feline urinary bladder UC. **a** Somatic mutations in genes mutated in three or more samples. Asterisk indicates significantly mutated gene (SMG). A summary of feline cases analyzed in this study is provided in Additional File 1: Table S1b and a list of all variants is provided in Additional File 4: Table S3. **b** Single base pair (SBS) mutational spectrum for sample CATD0037a (upper panel). The activities of mutational signatures SBS1, SBS6, SBS20, and SBS21 are shown in the reconstructed mutational spectrum (lower panel). **c** Indel mutational spectrum for sample CATD0037a, showing a prevalence of single base pair deletions in homopolymer regions. **d** Penetrance plot showing somatic copy number gains and losses 5 Mb or larger in 1-Mb windows along each chromosome. Only samples with sufficient quality, based on manual inspection of Sequenza plots, are represented (*n* = 21; see ‘‘Methods’’)

In contrast to canine UC, exome sequencing of the first feline UC cohort to date revealed similarity to human MIBC, as the most frequently mutated gene was *TP53* (14/23, 61%). Notably, the majority of these mutations were loss-of-function mutations (Fig. 2a). No mutually exclusive or co-occurring mutated genes were found in the feline UC cohort.

In the bovine UC cohort, all cases had mutations in *CSMD3*, *LRP1B*, and *ROS1*. Interestingly, cow BTAUD0031 had two independent primary UC lesions (BTAUD0031a and BTAUD0031c) and the only mutation they shared was in the putative tumor suppressor *LRP1B* (p.S2686P), implicating this as a driver mutation in the tumors from this cow. *HRAS* activation has previously been suggested to represent an early event of the PT carcinogenesis model [33, 34]; however, only one sample (BTAUD0031c) had a mutation in *HRAS* (p.G12D, which is homologous to human HRAS p.G12D) and we did not find any non-silent mutations in *KRAS* or *NRAS*, which suggests that RAS oncogenes are not frequent drivers of BF-induced carcinogenesis.

**Fig. 3:**
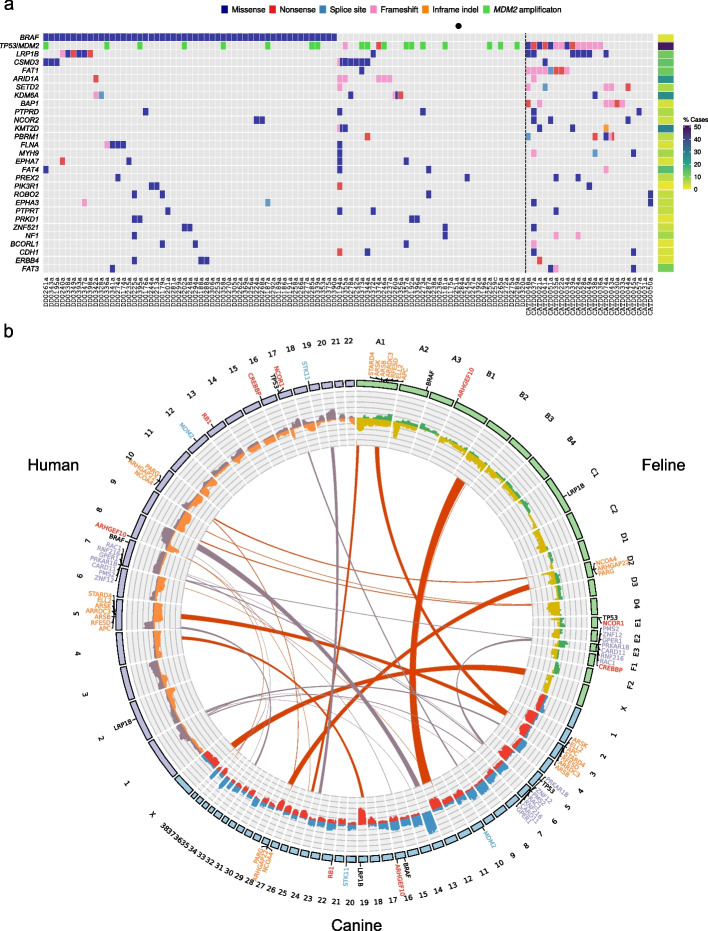
Comparative mutational landscape of human, canine, and feline urinary bladder UC.** a** The proportion of human MIBC cases [22] (*n* = 412) with somatic mutations in COSMIC Cancer Gene Census genes that have a one-to-one orthologous relationship with both a canine and a feline gene. Shown are mutations present in 4 or more canine or feline samples, which are prefixed with DD and CATD, respectively. Also shown are canine samples with *MDM2* amplification, which is shown in the same row as *TP53* mutations, to enable visual comparison with feline and human *TP53* mutations. *CDKN2A* has not been included, as, although the feline cohort had 4 samples with *CDKN2A* mutations, Ensembl does not classify the human and feline genes as orthologs, and, in canines, human *CDKN2B* is designated an ortholog of canine *CDKN2A*. **b** Circos plot displaying genomic regions with recurrent somatic copy number alterations in human, feline, and canine UC. Chromosomes are represented by the outer track. Data for human chromosome X was not available. The histogram (inner track) shows the frequencies of copy number gains (purple, blue, and green) and losses (orange, red, and yellow) in human, canine, and feline, respectively. Links between chromosomes show syntenic regions within recurrently amplified/deleted chromosomes (feline and canine) or chromosome arms (human). Red links represent deletions and purple links represent amplifications. Genes shown in orange and purple text are in syntenic regions in chromosomes or chromosome arms that were recurrently deleted or amplified, respectively, in all 3 species. Genes in red and blue text are genes that were focally amplified or deleted in 2 or more species. *ARHGEF10* is the only gene focally deleted in all 3 species. Shown in black text are other genes of interest

Comparing canine and feline UC, the exome-wide somatic mutational profiles from these species shared only 5 recurrently mutated genes (defined as genes mutated in ≥ 5% of samples in both species): *ZFHX4*,* FSIP2*,* USH2A*,* LRP1B*, and *XIRP2* (Additional file 6: Fig. S2a). There was also little overlap when comparing COSMIC Cancer Gene Census (CGC) genes that have a one-to-one orthologous relationship with both a dog and a cat gene (Fig. 3a). Only *LRP1B* was among the top 5 recurrently mutated CGC genes in both canine and feline UC, with canine UC mainly characterized by mutations in *BRAF*, *LRP1B*, *CSMD3*, and *ARID1A*, and feline UC by mutations in *TP53*, *LRP1B*, and *FAT1* (Fig. 3a). Similarly, with the exception of *TP53* in feline UC, the most frequently mutated genes in human MIBC are not mutated at similar proportions in feline or canine UC (Additional file 6: Fig. S2b), possibly due to the higher mutation rates in human MIBC.

### Cross-species comparison of significantly mutated genes identifies common driver genes in human MIBC and animal UC

**Fig. 4:**
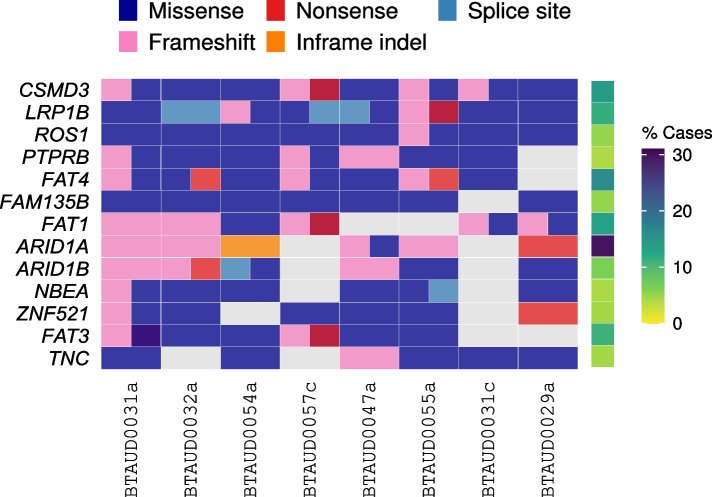
Recurrently mutated Cancer Gene Census (CGC) genes in bovine urinary bladder UC. Shown are COSMIC CGC genes mutated in at least 6 bovine UC cases (left), and the proportions of human UC cases [22] with mutations in these genes (right). Genes shown are those that had a one-to-one orthologous relationship between the human and bovine gene

We next identified significantly mutated genes (SMGs) in each species and compared them to those previously identified in human MIBC. In canine UC, we identified 9 significantly mutated genes (*BRAF*,* CSMD3*,* CDH12*,* ARID1A*,* PCDH17*,* KMD6A*,* ZNF804B*,* PCDH9*, and *GPRASP1*; Additional file 7: Table S5). There was no overlap with the 9 SMGs found in feline UC (*TP53*, *BAP1*, *FAT1*, *PBRM1*, *LRP1B*, *SETD2*, *NRAS*, *CDKN2A*, and *JAK1*; Additional file 8: Table S6). In bovine UC cases, with 4–16% (median 11%) of the exome affected by protein-altering mutations, candidate driver gene analysis was difficult. For example, when considering one-to-one orthologs of COSMIC CGC genes [35], we found 13 genes with one or more mutations in 6 or more samples (Fig. 4), and 54 genes were commonly mutated in at least half of the samples (Additional file 9: Fig. S3), indicating a high background mutation rate. Due to this limitation, and the small cohort size, no significantly mutated genes could be conclusively identified (see ‘‘Methods’’).

Comparison of SMGs found in canine and feline UC to those found in human MIBC revealed similarities that can guide refinement and prioritization of candidate gene lists in human UC. Robertson et al. [22] identified 58 SMGs in human MIBC. Of these, *ARID1A* and *KDM6A* are SMGs in canine UC, and *TP53*,* FAT1*, and *NRAS* and SMGs in feline UC, which indicated that these are key UC driver genes across species. In the bovine UC cohort, although the small sample size and high mutation rate prevented identification of SMGs, it is worth noting that *ARID1A* and *FAT1* were also mutated in 6/8 (75%) samples. In addition, *CSMD3* and *LRP1B*, which were identified as SMGs in canine and feline UC, respectively, were mutated in all eight bovine UC samples.

### Mismatch repair deficiency in canine and feline UC

A proportion of human MIBC patients (1.1–7.7%) show mismatch repair (MMR) deficiency [36–39]; thus, we searched for evidence of MMR deficiency in canine and feline UC. A recent study using immunohistochemistry of MMR proteins (MSH2, MSH6, and MLH1) found no loss of immunolabeling in ≥ 1 MMR proteins in canine UC; however, only 15 samples were examined [40]. In this study, in dog DD0355, we identified a somatic frameshift insertion and a germline frameshift deletion in *MSH2* (p.T234Yfs*22 and p.R1076X, respectively). In tumor sample DD0194a from another dog, we can predict that there has been biallelic somatic inactivation of *MSH2*, through frameshift mutation and loss of heterozygosity, as the allele frequency (AF) of the frameshift was 0.87 and this and 4 other upstream mutations have an AF of between 0.89 and 0.96 and fall near or within a predicted > 1 Mb deletion. Using SigFit [41] to fit known COSMIC mutational signatures [42], we identified single base substitution signatures SBS1 and SBS6 in the tumors of both of these dogs, and SBS21 in DD0194a (Fig. 1b); while SBS1 is found in most cancers and normal cells [43], SBS6 and SBS21 are associated with defective mismatch repair (dMMR) and microsatellite instability (MSI) [42, 44]. Additionally, the indel mutation spectra have similarity to COSMIC signatures ID2 and ID7 (Additional file 10: Fig. S4a), which are associated with dMMR and MSI. In line with dMMR and MSI, the tumor samples from both dogs had elevated single SNV and indel mutation rates (13.0 and 8.9 mutations/Mb, respectively) relative to the other canine samples.

Similarly, feline case CATD0037a, which had an elevated mutation rate relative to the other feline samples in the cohort (13.7 mutations/Mb compared to a median of 1.1 mutations/Mb; Fig. 2b), had two mutations affecting *MSH2*. We identified a frameshift deletion in exon 10 (p.G508Afs*18) and a single base change affecting the splice acceptor site of intron 1, which suggests both alleles were inactivated. Mutational signature fitting identified COSMIC mutational signature SBS1 and signatures associated with dMMR and MSI (SBS6 and SBS44), and, in line with dMMR and MSI, the indel spectrum had a prevalence of single base pair deletions in homopolymer regions greater than 5 bp in length (Fig. 2c). In addition to CATD0037a, CATD0050a had an elevated SNV and indel mutation rate (3.8 mutations/Mb); however, signature fitting and reconstruction did not result in any significant similarity to the observed mutation spectrum and no somatic or germline mutations were identified in *MSH2*, *MLH1*, *PMS2* or *MSH6.* Given that we identified *MSH2* mutations and corresponding SBS and ID mutational signatures in both feline and canine UC cases with high mutation burden, we can conclude that these samples are MMR-deficient, and similar to human MIBC, MMR deficiency plays a role in tumorigenesis in a subset of canine and feline bladder cancers.

### Characterization of a bracken fern-associated mutational signature

**Fig. 5:**
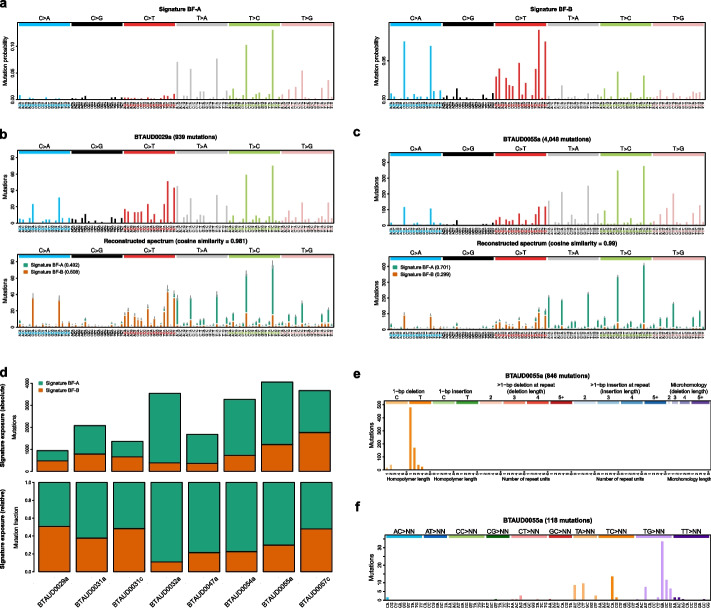
Mutational signatures in bovine UC. **a** Signature extraction identified 2 novel single base substitution (SBS) signatures, designated Signatures BF-A and BF-B. The SBS mutational spectra are comprised of 96 substitution types, which are derived from six possible SBS mutations, each with 4 possible bases directly 5′ and 3′. The observed and reconstructed SBS mutation spectra of the samples with the lowest and highest mutation rates, BTAUD0029a (**b**) and BTAU0055a (**c**), respectively, are shown. **d** The absolute (upper panel) and relative (lower panel) proportion of mutations attributed to Signatures BF-A and BF-B in bovine UC samples. **e** The indel mutation spectrum of BTAUD0055a. **f** The doublet substitution spectrum in BTAUD0055a

The bovine UC cases we sequenced in this study came from cows that had developed UC after grazing on pastures with bracken fern, which has been associated with UC in cattle. Bovine UC was distinctly different than canine and feline UC, with an extremely high mutation rate (median 65 mutations/Mb) and unique mutational signatures. The SBS mutational spectra of the bovine UC revealed a preponderance of T nucleotide substitutions in specific trinucleotide contexts. To characterize the underlying BF-induced mutagenesis, we extracted mutational signatures from single base substitutions, and profiled dinucleotide substitutions and indels. Based on goodness-of-fit (see “Methods”), it was determined that there were two SBS signatures, designated Signature BF-A and Signature BF-B, and both were active across all 8 bovine UC samples (Fig. 5a). There was no confident match with any known mutational signatures, as the highest cosine similarity between bovine Signature BF-A and a COSMIC or Signal [45] signature was only 0.49 and 0.63, respectively, and for bovine Signature BF-B, the highest cosine similarities were 0.68 and 0.73, respectively. Visual comparison of the spectra also confirmed the matches as low confidence (Additional file 11: Fig. S5). The mutation spectra of the samples with the lowest (BTAUD0029a) and highest (BTAUD0055a) mutation rates were reconstructed using these novel signatures yielding cosine similarities of 0.981 and 0.990, respectively (Fig. 5b,c). The activity (exposure) of Signature BF-A was responsible for 49–89% of mutations across the 8 bovine samples (Fig. 5d). In Signature BF-A, a large proportion of point mutations were T > C, with the majority occurring in trinucleotide context CTC and TTC, T > A in sequence context ATA, CTA or TTA, and there also was significant bias toward the genic transcribed strand (*p* < 0.01) in all of these contexts in two or more bovine UC cases (Additional file 12: Fig. S6). A number of T > G mutations also occurred in specific sequence contexts (Fig. 5a), with significant transcriptional strand bias (*p* < 0.01; Additional file 12: Fig. S6). Similarly, a novel indel signature involving deletion of T was identified in all 8 bovine UC samples (Fig. 5e). Interestingly, the majority of T deletions did not occur in homopolymer runs, but rather in a CTG or CTC trinucleotide context (Additional file 13: Fig. S7), something not seen in COSMIC indel signatures. Finally, although there were far fewer dinucleotide variants, the majority were TC > CA and TG > GN (Fig. 5f); again, this does not match any known COSMIC doublet base substitution (DBS) signature. In summary, there is a strong pattern of substitution and deletion of T nucleotides in specific trinucleotide contexts, and these signatures do not resemble any COSMIC or Signal signatures.

**Fig. 6:**
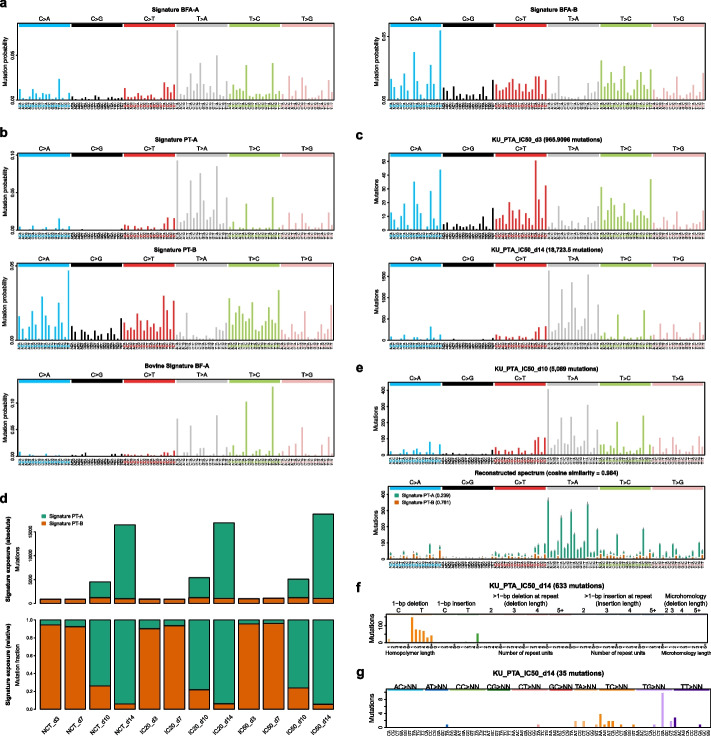
Mutational signatures in human bladder cancer cell lines after exposure to bracken extracts and ptaquiloside. **a** Signature extraction identified 2 novel single base substitution (SBS) signatures, Signatures BFA-A and BFA-B in KU-19–19 cells exposed to BF whole extract (BFA). **b** Similar signatures, Signature PT-A and PT-B, were identified in KU-19–19 cells exposed to purified PT. For comparison, bovine Signature BF-A is shown (lower panel). **c** The SBS mutation spectra after 3 days (upper panel) and 14 days (lower panel) of PT exposure at IC_50_. **d** The absolute (upper panel) and relative (lower panel) proportion of mutations attributed to Signatures PT-A and PT-B in KU-19–19 cells exposed to PT. NTC is non-toxic concentration; IC_20_ and IC_50_ are 20 and 50% inhibitory concentration (of cell growth), respectively; d3, d7, d10, and d14 are the number of days. **e** The observed and reconstructed SBS spectra from KU-19–19 cells exposed to PT for 10 days (IC_50_), showing the activity of each signature for each substitution type. **f** The indel mutation spectrum observed at day 14 (IC_50_) in KU-19–19 cells exposed to PT. **g** The doublet substitution spectrum observed at day 14 (IC_50_) in KU-19–19 cells exposed to PT

It has been shown previously that DNA alkylation by ptaquiloside has a strong preference for adenine bases [33, 46]; therefore, we next asked whether BF exposure, or specifically, PT was responsible for the mutational signatures we observed. We generated whole BF extracts, from freshly collected BF fronds (Additional file 14: Fig. S8) using two methods, acetone extraction (BFA) and ethyl acetate extraction (BFE), and treated human urinary bladder UC KU-19–19 cells every 24 h for 3–14 days (see “Methods”). The BF extracts had increased cytotoxicity in chemosensitivity dose response assays with increasing number of days of exposure; thus, the IC_20_ and IC_50_ concentrations of the BF extracts applied to the cells for mutational analyses were different for each time point (with longer exposure times requiring lower doses; Additional file 15: Table S7 and Additional file 16: Table S8). Using mutations pooled from all BF extract doses and time points, mutational signature extraction was performed (see “Methods”). Based on goodness-of-fit (see “Methods”), it was estimated that two SBS signatures, which we designated in vitro Signatures BFA-A and BFA-B, were active in the human UC cells treated with BFA (Fig. 6a). The relative exposure of each signature within each individual sample is shown in Additional file 15: Table S7. The cosine similarities between the observed mutational spectra in the BFA-treated cells and the mutational spectra reconstructed from the extracted signatures at each dose and time point were between 0.974 and 0.992. An example from cells exposed to BFA for 3 days (at IC_50_) is shown in Additional file 17: Fig. S9a. The in vitro Signatures BFA-A and BFA-B highly resembled the two in vitro SBS signatures active in cells treated with BFE (cosine similarity = 0.94 and 0.98 to Signatures BFE-A and BFE-B, respectively; Additional file 17: Fig. S9b-c); henceforth, the discussion will focus on the signatures identified in cells treated with BFA. In vitro Signature BFA-A did not confidently match any known mutational signature, with the highest cosine similarities being 0.7 to COSMIC SBS25 and 0.74 to Signal SBS141 (Additional file 11: Fig. S5a). Additionally, a visual comparison of the mutational spectra for COSMIC SBS25 and Signal SBS141 do not contain the T > C and T > A peaks in specific sequence contexts, which are the distinguishing feature of the novel Signature A observed in bovine UC and in vitro. Signature BFA-A had a good resemblance to the bovine UC Signature BF-A that we identified (cosine similarity = 0.82), with a prevalence of T mutations in specific trinucleotide contexts (Fig. 6a). In vitro Signature BFA-B did not resemble bovine UC Signature BF-B (cosine similarity = 0.57). In vitro signature BFA-B had cosine similarities of 0.84 to 0.87 to three very distinct known signatures, COSMIC SBS40, Signal SBS18 and Signal SBS167, which is annotated as tentative or having a lack of evidence (Additional file 11: Fig. S5b), which provides some uncertainty as to whether any of these are true matches. In summary, there is a good resemblance between the bovine UC Signature BF-A and the in vitro Signature BFA-A that was seen in the BF extract-treated human UC cell line, and these signatures do not resemble any COSMIC or Signal signatures. The number of indels found in BFA-treated cells ranged from 108 to 193 and we did not find a propensity toward deletion of T in CTG or CTC sequence context as we did with bovine UC.

To determine if PT was the component of BF extract primarily responsible for the mutational signatures that we identified in the cells treated with BF extract and in bovine UC tumors, and to investigate the effect of exposure time, we purified PT from BF fronds and treated KU-19–19 cells every 24 h at a fixed concentration (non-toxic dose (NTC), 0.2 µM; IC_20_, 10 µM; and IC_50_, 30 µM), and collected the cells from 3 to 14 days after the first day of exposure (see “Methods”). Signature extraction was performed (see “Methods”), using mutations from all time points and PT doses. Based on goodness-of-fit (see “Methods”), it was estimated that two SBS signatures, which we designated in vitro Signature PT-A and PT-B, were active. The in vitro signatures BFA-A and BFA-B, found in cells treated with BF extract were recapitulated in cells treated with PT (cosine similarity = 0.91 when compared to PT-A and 0.97 when compared to PT-B), suggesting that PT was indeed associated with the observed signatures. When in vitro signature PT-A was compared to bovine UC signature BF-A, the resemblance to bovine UC Signature BF-A was lower (cosine similarity = 0.77); however, the signatures shared the key features of the bovine Signature BF-A, specifically, T mutations in specific mutational contexts and a lack of C mutations (Fig. 6a, b and Additional file 17: Fig. S9b). Additionally, the indel and doublet substitution profiles from cells treated with PT mirrored those from bovine UC.

Of the known signatures, Signature PT-A had the highest cosine similarity to COSMIC SBS90 (0.75) and Signal SBS90 (0.78). Of note, although similar to in vitro signature PT-A in sharing some peaks of T > A mutations in specific sequence contexts, SBS90 lacks the distinctive T > C mutations observed in bovine UC and the in vitro signatures PT-A and BFA-A (Additional file 11: Fig. S5a), and we therefore concluded that Signature PT-A was novel. Similar to in vitro signature BFA-B, PT-B has cosine similarity to COSMIC SBS40 and two Signal signatures (cosine similarity 0.83 to 0.88), and with visual inspection, it was unclear whether any of the signatures represents PT-B (Additional file 11: Fig. S5b).

Similar to bovine UC, there was a significant bias toward the genic transcribed strand for T > C, T > G, and T > A mutations in specific trinucleotide contexts (Additional file 18: Fig. S10). Strand bias was observed in more highly expressed genes, which also had lower a mutational burden; this suggests transcription coupled repair activity rather than transcription coupled damage. We did not find replication-associated strand bias.

To determine the effect of PT dose over time, we compared mutational spectra for each time point. At all doses of PT, a similar pattern was observed, where the mutational spectra seen at day 3 resembled Signature PT-B, and, after the accumulation of more mutations, the spectra at day 14 resembled Signature PT-A. Examples of day 3 and day 14 mutational spectra from the IC_50_ dosage are shown in Fig. 6c. Indeed, in the earlier time points (days 3 and 7), Signature PT-B was predominately active, whereas in the later time points (days 10 and 14), the number of mutations increases due to Signature PT-A activity (Fig. 6d). The cosine similarities between the mutational spectra reconstructed with Signatures PT-A and PT-B and the observed mutational spectra at each dose and time point were 0.950–0.999. An example from cells treated with PT for 10 days (at IC_50_) is shown in Fig. 6e.

Similar to SBSs, the mutational spectra of indels in PT-exposed KU-19–19 cells showed more similarity to the bovine UC indel mutational spectra at later time points, with an increasing proportion of T deletions occurring outside of homopolymer runs (Additional file 19: Fig. S11). For cells treated with PT for 14 days (at IC_50_; Fig. 6f), deletion of T was the most common indel, as it was in bovine UC. For 1 bp deletions occurring outside of homopolymers, although there was a preponderance for deletion of T, as observed in bovine UC, there was a wider preference for sequence context ATN and CTN (Additional file 20: Fig. S12) rather than primarily in the CTG or CTC context. Although we did not observe an obvious preference for T deletions in sequence-specific contexts in cells treated with BFA, this maybe be due to the difference in the dosage protocols used (see “Methods”); for longer time periods, lower doses of BFA were required for cell survival, whereas a consistent dose of PT over various treatment times showed that only after 14 days, the indel profile in treated cells was similar to that found in bovine UC. Also consistent with bovine UC, there were relatively fewer dinucleotide variants than SBSs and indels, and the majority of these were TG > GA (Fig. 6g).

In summary, we have characterized a unique signature in bovine UC, BF-A, consisting of mutation and deletion of T/A nucleotides in specific mutation contexts, and there is a good resemblance of novel signatures found in the bovine UC and the in vitro signatures found in the BF- and PT-treated UC cell lines (Additional file 11: Fig. S5). Treatment of cell lines with BF extract and purified PT produced highly similar mutational signatures, and the increase in mutations over time in PT-treated cells was predominately due to the activity of Signature A, which implicates PT as the primary mutagen associated with this signature.

### Germline predisposition variants

As we sequenced matched normal tissue for each tumor in our cohorts, we were able to search for putative pathogenic germline variants. We focussed on genes with nonsense and frameshift variants, as these variants will have a predictable detrimental effect on gene function. In canine UC cases, we identified nonsense variants in 6 genes, and frameshift variants in 27 genes (Additional file 21: Table S9). As discussed above, we identified a germline frameshift variant in *MSH2* in dog DD0355; *MSH2* has been validated as a UC risk gene in humans, with germline pathogenic variants in *MSH2* reported in 1.4–3.5% of UC patients [47, 48]. In addition, germline samples DD0191b and DD0281b had frameshift variants in *NBN*, which is a moderate-penetrance gene with pathogenic or likely pathogenic germline variants in 0.5% of human patients with UC [48]. Another canine UC case had a *ATM* germline frameshift mutation; *ATM* has also been highlighted as a potential human UC predisposition gene [47]. In feline UC cases, we identified germline nonsense variants in 9 genes, and frameshift variants in 10 genes (Additional file 22: Table S10). Importantly, a frameshift variant in *CHEK2* was identified in one cat (CATD0037); 1% of human MIBC patients have been found to have pathogenic or likely pathogenic variants in *CHEK2*, which is a moderate-penetrance gene [48]. In bovine UC cases, we identified nonsense variants in 9 genes, and frameshift variants in 7 genes (Additional file 23: Table S11). However, none of these genes have previously been reported to have pathogenic variants in human UC patients. Comparing across species, we also note the presence of germline loss-of-function variants in *SMAD3*, *POLQ*, and *CBFA2T3* in both canine and feline UC cases, *BARD1* in feline and bovine UC cases, and *TSC2* in canine and bovine UC cases. Further studies are required to determine if these are true UC predisposition variants, and whether any of these candidates can inform on human predisposition to MIBC.

### Cross-species comparative analysis of somatic copy number alterations

Analysis of the SCNA profiles of the canine UC samples (*n* = 62) derived from exome sequencing data revealed substantial chromosomal gains and losses, the most frequent of which were copy number (CN) gains along chromosomes 13, 36, and 38 and CN losses along chromosomes 5, 12, and 19 (Additional file 10: Fig. S4b). This pattern is similar to that identified in canine primary UC by oligonucleotide array comparative genomic hybridization (oaCGH; *n* = 31) [49], in which CN gain of chromosomes 13 and 36 and loss of chromosome 19 were most prevalent. In feline UC (*n* = 21), the most frequent CN gains were found on chromosome E3 and the most frequent losses were found on chromosomes A1 and D4 and part of chromosome A2 (Fig. 2d). From the bovine UC sample cohort, only 3/8 samples had SCNA profiles suitable for CN analysis (BTAUD00029a, BTADU00031c, and BTAUD00055a; see “Methods”); however, it is worth noting the paucity of structural variants in these samples relative to human, canine, and feline UC, with only chromosomal copy-neutral LOH of one chromosome per sample (chromosomes 11, 29, and 7, respectively) and very few focal SCNAs (Additional file 24: Fig. S13). The lack of SCNAs in bovine UC is in line with the previous observation that there is an inverse relationship between the number of somatic mutations and SCNAs [50]. Additionally, PT preferentially alkylates adenine bases [51] leading to small DNA aberrations rather than genomic instability.

Chromothripsis has been observed in human MIBC [52], with one study identifying 11/23 (47.8%) UC samples with low- or high-confidence chromothripsis events [53]. Using the criteria described by Voronina et al. [54] to score chromothripsis predications as high, intermediate, and low confidence (see “Methods”), we identified chromothripsis-like events in 60% of canine UC samples (37/62) for which we had high-quality SNCA profiles (Additional file 10: Fig. S4c; see “Methods”). An example of a canine UC sample with chromothripsis-like events on two chromosomes is shown in Additional file 10: Fig. S4d. Chromothripsis-like events were most frequently found on chromosome 36 (*n* = 10 samples), followed by chromosome 10 (*n* = 7), which is similar to that previously identified in canine primary UC by oaCGH [49], where they estimated 74% of cases had 1 or more chromothripsis-like events. There were also some notable differences; for example, we also identified chromosome 9 as a frequent target of chromothripsis-like events, while Shapiro et al. found 16% of samples with chromothripsis-like events on chromosome 16, which we did not observe. These differences may be due to the differences in the criteria for defining chromothripsis-like events and/or differences in the technologies used to detect SCNAs. These chromothripsis events could be confirmed by combining structural variant and CN analysis and performing whole-genome sequencing [53]. As with canine UC, we identified chromothripsis-like events in feline UC samples. Of the 8/21 (38.1%) samples with chromothripsis-like events, chromosomes A2 and E1 were most frequently affected (Additional file 25: Fig. S14a). An example of a feline UC sample with chromothripsis-like events on 3 chromosomes is shown in Additional file 25: Fig. S14b.

A cross-species comparison between canine UC, feline UC, and previously analyzed human MIBC [22] found that within recurrently amplified or deleted chromosomes, or chromosome arms in the human MIBC samples, there were only three regions with CGC genes and synteny between all three species, including a recurrently deleted region containing the tumor suppressors *APC* and *ARRDC3* (Additional file 26: Table S12 and Fig. 3b). We next used STAC [55] to identify significantly amplified and deleted sub-chromosomal regions in dog and cat samples (see “Methods”) and compared these to significant SCNAs previously identified in human MIBC (Additional file 27: Table S13 and Fig. 3b). In focal regions less than 10 Mb, we found significant recurrent amplification of the oncogene *MDM2* and deletion of the tumor suppressor and DNA repair gene *RB1* in canine samples, which is also the case for human MIBC [22]. In the feline UC samples, it was interesting to note deletion of the transcriptional co-activators and chromatin remodelling genes, *CREBBP* and *NCOR1*, as 57/97 (59%) of human UC patients harbor nonsynonymous mutations in chromatin remodelling genes (including *CREBBP* and *NCOR1*) [56], which suggests that aberration of chromatin regulation might be a hallmark of urinary bladder cancer [56]. To identify further genes of interest, we expanded the cross-species comparison to wider peak regions identified by STAC and GISTIC analyses (Additional file 27: Table S13). The guanine nucleotide exchange factor family member *ARHGEF10*, a candidate tumor suppressor gene (TSG) which has reduced *ARHGEF10* expression in > 50% human UC cell lines [57], was the only gene significantly deleted in all three species, providing further support that *ARHGEF10* is an important TSG in UC.

In summary, with WES, we have been able to recapitulate canine SCNA profiles observed with oaCGH [49] and offer a first glimpse of SNCA in feline and bovine UC. Chromothripsis occurs in canine and feline UC, in line with previous reports of chromothripsis in human MIBC [52]. As with somatic mutations, cross-species analysis enabled the identification of common significantly amplified or deleted genes as key driver events in bladder cancer.

## Discussion

Advances in genome sequencing have enabled comprehensive cataloging of mutations and copy number events in cancer. However, distinguishing between passenger and driver mutations remains challenging. Cross-species analysis is one method that can contribute to advancing our understanding of tumorigenesis. Firstly, identification of SMGs or SCNAs that are common between species can help prioritize and refine candidate driver genes and potentially refine our mechanistic understanding of cancer gene function. Secondly, elucidation of the similarities and differences in the oncogenomic landscape of tumors in non-human species allows us to determine whether they represent relevant models that can be utilized as a means to improve and expedite our understanding of cancer biology and potential therapies.

Domestic dogs and cats spontaneously develop tumors that share many similarities with human tumors, including anatomical location, histological appearance and therapeutic response, and canines in particular have been proposed as a model of human MIBC (reviewed in [58]). The transcriptomes of small canine UC cohorts have been studied (*n* = 4 to *n* = 18) [26, 28, 59–62]; however, only two studies have performed WES on canine UC, with their findings limited by small sample size and a lack of matched normal tissue (*n* = 3/11 [28] and *n* = 0/28 [32] of tumor samples had normal tissue samples from the same animal). In contrast, there have been no whole-exome analyses of feline UC, likely as a consequence of both the relative low frequency of occurrence (0.38–0.56% of all feline malignancies [63, 64] versus 1.5–2% of all canine neoplasms [65]), and the comparatively lower investment made in sequencing feline cancers [66]. Therefore, in this study, we sequenced the exomes of canine and feline UC, which not only provided insights into UC in these companion animals, in line with the ‘One Medicine, One Health’ approach [67], but also allowed us to identify conserved genetic alterations involved in tumor development in human MIBC by leveraging cross-species comparative analysis.

Canine and feline UC are genetically heterogeneous, as is human MIBC, and both shared some aspects of the mutational landscape of human MIBC, as well as important differences. The most striking similarity was the significant proportion of samples with *TP53* mutations in both feline UC and human MIBC. We did not, however, find the co-occurrence of mutation of *TP53* and *RB1*, that is observed in human MIBC [22]. Mutation of *TP53* was notably absent in canine UC; however, like human MIBC, *MDM2*, which is a negative regulator of *TP53* [68] and *RB1*[69], was significantly amplified in canine UC (Fig. 3), and *RB1* itself was also significantly deleted. This suggests that disruption of the p53 pathway, through amplification of *MDM2* rather than *TP53* mutation, may be a key driver of tumorigenesis in canine UC. Importantly, *RB1* was one of 3 genes, that, if mutated, was found to predict response and benefit from cisplatin-based neoadjuvant chemotherapy for human MIBC [70]. The most striking difference between canine UC and human MIBC was the high proportion of canine UC tumors with BRAF p.V588E (V595E) mutations, which corresponds to the human BRAF p.V600E hotspot mutation. This mutation has previously been reported in 65–87% of canine UC [25–28], and *BRAF* mutation testing of DNA in urine samples has emerged as a non-invasive diagnostic option for canine UC [71]. However, *BRAF* is infrequently mutated in human UC and MIBC [22, 72, 73], is not mutated in any of the 23 feline UC cases and is mutated in only 1 of the 8 bovine UC cases, which suggests a different etiology for tumorigenesis in dogs.

While a subset of human MIBC with a high mutational load are associated with APOBEC activation signatures [22], we did not find elevated mutation rates in feline or canine UC cases other than those associated with MMR deficiency. Although de novo signature extraction was limited by the low number of mutations available for analysis, it is unlikely that APOBEC-mediated mutagenesis signatures are present, given the very low mutation rate. Nonetheless, performing whole-genome sequencing or collecting much larger WES data sets may enable discovery of other signatures that contribute to a lower mutational burden. One feline and two canine samples had elevated mutation loads that are attributed to MSI/dMMR from loss-of-function mutations in *MSH2*. *MSH2* is an established UC risk gene in humans [47, 48]; UC is the third most common Lynch syndrome-associated tumor [74], with increased risk of urinary bladder UC reported in Lynch syndrome patients carrying *MSH2* mutations [75]. Previous studies have reported MSI in 1.1% of human urinary bladder UC patients [76] and dMMR in 1.1–7.7% of patients [36–39]. Defective MMR in urinary bladder UC shows temporal and spatial homogeneity throughout the tumor [37], and there is a strong correlation with cytotoxic T lymphocyte infiltration and PD-L1 tissue expression [36]. Indeed, there is a case report showing complete response to anti-PD-L1 antibody (atezolizumab) in metastatic MIBC patient that had MSI associated with a novel *MSH4* somatic mutation [77]. Thus, not only do a proportion of canine and feline UC cases potentially represent a model of MSI/dMMR-mediated urinary bladder UC, they themselves may also benefit from immune checkpoint inhibitor therapy.

With a high mutation rate and nearly 60 SMGs identified in human MIBC [22], it is difficult to identify true driver genes and driver events. Cross-species comparative analysis of SMGs in canine, feline, and human UC enabled the refinement of *ARID1A*, *KDM6A*, *TP53, FAT1*, and *NRAS* as key driver genes in human MIBC. Similarly, while somatic CN analysis of human MIBC identified numerous genes within significantly amplified or deleted chromosomal regions [22], cross-species comparison of significant copy number changes revealed a small overlap between UC in the 3 species. This enabled further refinement of relevant CN changes and identification of key driver events in tumorigenesis across species, including amplification of the oncogene *MDM2*, deletion of the tumor suppressor *RB1* and deletion of the candidate tumor suppressor gene *ARHGEF10*, which was the only significantly deleted gene in UC in all 3 species. Additionally, we identified deletion of chromatin remodelling genes *CREBBP* and *NCOR1* as common driver events in human and feline UC.

BF exposure has been linked to esophageal and gastric cancer in humans (reviewed in [78–81]). Exposure can occur directly by consumption of the plant or by spore inhalation, and PT, a carcinogen found in BF, has been found in the milk of cows grazing on BF and in surface and ground water [21, 78, 82, 83]. It has been estimated that PT accounts for > 50% of the carcinogenic potency of BF [84]. The carcinogenic effect of PT is based on its hydrolysis and the formation of a dienone intermediate (APT) that can produce DNA adducts (via alkylation), which are responsible for inducing carcinoma [51]). Cattle do not commonly develop UBC; however, those that have grazed on BF pastures can develop bovine enzootic hematuria due to chronic BF toxicity, which results in urinary bladder hemorrhages and the development of multiple lesions in the urinary bladder wall, most of which are UC (67%) [17]. DNA adducts have been detected in the ileum of calves that were fed BF [33], and in the upper gastrointestinal tissues of mice that were fed BF extract or spores [85], providing direct evidence for BF-induced carcinogenesis.

Sequencing of bovine UC revealed mutational profiles vastly different to those in spontaneous UC arising in cats, dogs, and humans. We identified novel SBS, DBS, and indel mutational signatures that were present in all eight bovine urinary bladder UCs we sequenced, which arose in cows that grazed on pastures with BF in Portugal and Brazil. In line with the previous observation that APT preferentially alkylates adenines [33, 86], the predominant signatures in bovine urinary bladder UC were deletion of T in the CTG or CTC context and point mutation of T in specific dinucleotide and trinucleotide contexts. Notably, we were able to identify similar mutational signatures in a human UC cell line exposed to either BF extracts or purified PT, providing evidence that PT exposure was the main contributor to the mutational profiles observed in bovine UC. Bovine Signature BF-B was not recapitulated by the human cell line experiments; it could represent other mutational processes active in bovine UC that were not present in vitro. Additionally, the second SBS signature extracted from the human UC cell line experiments, Signature BFA-B, was not observed in bovine UC, which could be due to processes occurring in vitro or the fact that the cell population was not clonal.

## Conclusion

We have identified the key similarities and differences between the genetic landscape of spontaneously arising urinary bladder UC in pet dogs and cats and MIBC in humans. The similarities show that both canine and feline UC could be informative as models for human MIBC, which has the additional benefit of informing further investigation of UC of these companion animals as well has human MIBC. Cross-species comparative analysis was used to prioritize the top candidate driver genes and copy number events in human MIBC, which will help focus future research into treatment options. Finally, we identified in BF-consuming cattle an extremely high mutational load and novel mutational signature, characterized by point mutation and deletion of T/A nucleotides in specific sequence contexts. In vitro recapitulation of this signature in cell lines implicate PT as the mutagen. These findings could have implications for studies examining the health effects of BF and PT exposure in humans.

## Methods

### Sample collection and DNA isolation

The samples of urinary bladder urothelial carcinoma consisted of formalin-fixed, paraffin-embedded (FFPE) canine, feline, and bovine tissues that had been collected as part of routine diagnostic procedures or necropsy (or at the slaughterhouse), with the owner’s consent. The use of the samples adhered to Nagoya Protocol guidelines. The cases were selected based on the availability of matched FFPE normal (healthy) tissue from the same animal (which in some cases was urinary bladder tissue adjacent to the tumor and in other cases was a different tissue altogether) and from a range of breeds and institutions in different countries. The country from which the case was obtained, the tissue that was sampled and the signalment data for each case, including the species, tumor and normal tissue sampled, breed, sex, neutering status, and age at diagnosis is provided in Additional file 1: Table S1. The 87 canine cases (29 male, 58 female) represented 36 different breeds and were collected from 20 institutes across 16 countries. The 23 feline cases (14 males, 9 females) represented 6 different breeds and were collected from 8 different institutions across 8 countries. The 8 bovine cases (from 7 different female cows, as one cow had 2 independent lesions) were collected from 3 different institutes across 2 countries. The institutions were a mixture of private veterinary pathology companies and university or governmental veterinary pathology departments. All cases were examined by experienced pathologists, who then annotated the tumor and normal areas to be sampled. All tumor and normal tissue samples were obtained as either 0.6 or 1-mm-diameter cores or as unstained 10-micron-thick tissue sections attached to glass slides. Genomic DNA was extracted from the tumor and normal cores or unstained tissue sections (scraped from the tumor and normal areas on the slides) using a QIAamp DNA FFPE Tissue Kit (Qiagen), according to the manufacturer’s instructions.

### Whole-exome bait design

Agilent SureSelect bait libraries were designed for the canine, feline, and bovine exomes using gene models from Ensembl v98 and genome references CanFam3.1 [87] (OLIDs: 3263651, 3263641, 3263631), Felis_catus_9.0 [88] (OLIDs: 3261601, 3261611, 3261621), and ARS-UCD1.2 [89] (OLIDs: 3263141, 3263131, 3263121), respectively. Baits were designed against regions in protein-coding transcripts annotated on the main autosomes and chromosome X, with an additional 25 bp flanking each side. In the final bait design, 7.4, 5.1, and 7.2% of the original canine, feline, and bovine coding regions targeted had no bait coverage. Balanced bait boosting was applied for high GC regions.

### Sequencing, read alignment, and quality control

Sequencing libraries were prepared from the FFPE-extracted DNA as previously described [90] and were pooled (8-plex) in an equimolar fashion and hybridized with baits overnight. The multiplexed samples were paired-end sequenced using the NovaSeq platform (Illumina) to generate 101-bp reads.

Sequencing reads from canine, feline, and bovine samples were aligned to the CanFam3.1, Felis_Catus_9.0, and ARS-UCD1.2 reference genomes, respectively, using BWA-MEM (v0.7.17-r1188) [91]. PCR duplicates were marked using Biobambam2 bammarkduplicates (v2.0.29) [92]. Samples with contamination, sample swaps, and less than 11x coverage across 80% of the targeted regions were excluded. Canine and feline tumors where 95% of the VAFs from somatic mutation calling was ≤ 0.25 were also excluded. In total, there were 87 canine, 23 feline, and 8 bovine matched tumor-normal pairs. The mean sequence coverage of targeted regions was 123x, 113x, and 86x for these canine, feline, and bovine samples, respectively, when PCR duplicates were excluded.

### Variant calling

Somatic SNVs were identified using MuTect (v1.7) [93]. Default parameters were used, with the exception of a minimum base quality score requirement of 30 and a maximum of three alternative alleles allowed in a matched normal sample. MAC (v1.2) [94] was used to identify multi-nucleotide variants from MuTect output by identifying adjacent SNVs on the same strand. Strelka2 (v2.9.10) [95] was used to identify small indels using default parameters, with the empirical variant scoring (EVS) option disabled. Gene models and the Variant Effect Predictor (VEP) [96] from Ensembl v98 were used to predict the consequences of base changes and indels on proteins. The canonical transcript, as defined by Ensembl, was used to determine the variant consequence. Common SNVs, defined as variants present in 1% or more of the reference SNV databases listed below, were removed. We identified C > T transition artifacts from deamination of cytosine present at low variant allele frequency (VAF) and attempted to remove these by removing C > T (or G > A) with VAF < 0.1, total depth of coverage < 20x or VAF < 0.2 if the coverage was 20-99x. We also identified artifacts with similar mutational profiles as COSMIC signatures SBS45 and SBS52, and therefore removed C > A (or G > T) transversions that occurred in sequence context CCN or TCN (NGG or NGA for G > Ts) if the VAF was < 0.1, the depth of coverage was less than 20, or the coverage was 20-99x and the VAF was < 0.2. Additionally, general filtering was applied and variants were removed if the tumor or matched normal depth of coverage was < 10x , if the coverage < 300x and VAF in the tumor was < 0.1 or if the coverage ≥ 300x and the VAF in the tumor was < 0.05. Additionally, the VAF in the matched normal was required to be < 0.01. DISCOVER (v.0.9.4) [31] was used to search for mutually exclusive and co-occurring somatic mutations.

Catalogs of known variants in the cat, dog, and cow genomes were obtained from the 99 Lives Cat Genome Consortium (v9, from 54 cat genomes)[88], the National Human Genome Research Institute (NHGRI) Dog Genome Project [97], and the 1000 Bull Genomes Project [98], respectively. The VAFs from these databases were used to remove any common variants (AF ≥ 0.01) from the somatic variant calls. Variants in the normal germline samples were identified using the Genome Analysis Toolkit (GATK, v4.2.4.1) [99]. GATK HaplotypeCaller was run with a minimum base quality score 20 and soft-clipped bases were not used. This was followed by CombineGVCFs, GenotypeGVCFs, and SelectVariants to create a file for SNVs and another for indels. Finally, GATK VariantFiltration was run, with the following options for SNVs: QC < 2, QUAL < 30, SOR > 3, FS > 60, MQ < 40, MQRankSum < -12.5, ReadPosRankSum < -8.0. For indels, the options were as follows: QD < 2, QUAL < 30, FS > 200, ReadPosRankSum < -20. Ensembl VEP was run, as described above, to predict variant consequences. To identify candidate risk alleles in the canine, feline, and bovine germlines, variants with a population allele frequency (AF) > 0.001 in the corresponding variant databases (described above) were removed, along with multi-allelic sites, and sites where more than half of the samples were not genotyped. Finally, we selected variants resulting in frameshift and nonsense mutations in genes that had a one-to-one orthology with a CGC gene.

### Somatic copy number alterations

Sequenza (v3.0.0) [100] was used to identify allele-specific SCNAs from aligned tumor and matched normal sequence reads (described above). All results were manually curated, and, where applicable, alternative ploidy and purity estimates were used to replace the default best-fit solutions, as previously described [100]. While Sequenza does not provide a specific measurement to evaluate noise and the quality of the estimates, various plots are provided for this purpose. Manual curation included visual inspection of the model fit plots, which show the correlation of the B-allele frequencies (BAF) and depth ratios with joint log posterior probability (LPP) density, the contours plot, which show the LPPs of a range of ploidy and purity combinations, and genome plots showing BAF and depth ratios, as described in the Supplemental Materials of Favero et al. [100]. Samples that were deemed to have excessive noise were excluded from CN analysis. Sequenza provides absolute copy number calls for segments, therefore, to determine if a segment represents a relative CN gain or loss, the tumor ploidy was first determined as the most frequent CN assigned to segments with mean B-allele frequencies > 0.3, and a CN gain or loss was called if the absolute CN of the segment was above or below the ploidy, respectively. STAC [55] was used to find significant CN gains and losses in the canine and feline samples. Regions of gain or loss with frequency *p*-value < 0.05 or footprint *p*-value < 0.05 were considered statistically significant. The derivation of the frequency and footprint *p*-values are described in the STAC publication [55].

### Significantly mutated genes

To identify candidate driver genes, we used dNdScv (v0.0.1.0, git commit ID 0633182) [101], which identifies genes under positive selection, and MuSiC2 [102], which uses multiple statistical tests to identify genes significantly mutated above a background mutation rate. The dNdScv reference databases for each species were built using Ensembl v98 canonical transcripts and only genes targeted in bait design were included. Genes with a *q*-value < 0.01, when considering either substitutions only or all mutation types, were considered significant. For MuSiC2 analysis, genes with an FDR < 0.01 from the convolution test were considered significant. The convolution test is described in the original MuSiC publication [102].

### Somatic mutational signatures

For canine and feline UC samples with elevated mutation rates, SigFit (v2.2) [41] was used to fit COSMIC mutational signatures (v3.2). Using the mutational opportunities calculated from the canine or feline exome, including exons plus 2 bp flanking each exon to account for splice sites, mutational catalog counts were converted relative to the human genome. The SigFit ‘fit_signatures’ function was then used to fit COSMIC signatures (v3.2), using 10,000 sampling iterations. Signatures contributing to more than 5% of mutations were used to refit signatures, and the combination of signatures that resulted in the highest cosine similarity after mutation spectra reconstruction was chosen as the best solution.

For bovine UC samples, SigFit (v2.2) [41] was used to extract mutational signatures de novo by first converting mutational catalog counts relative to the human genome, using mutational opportunities calculated from the bovine exome plus 2 bp flanking each exon. Signature extraction was performed using 10,000 sampling iterations and a goodness-of-fit plot was generated using the SigFit functions ‘calculate_gof’ and ‘plot_gof’ to estimate the optimal number of signatures. Signature re-fitting was then performed using the extracted signatures in order to calculate the signature exposures per sample. Signature extraction was performed in the same manner for the mutational catalogs from canine and feline UC, converting mutational counts relative to the human genome using mutational opportunities calculated from the canine and feline exome plus 2 bp flanking each exon. Signature extraction was performed for mutational catalogs from the human urinary bladder cells as described above, without conversion of the catalog counts. All novel signatures were compared to COSMIC signatures (v3.2) [42] and Signal [45] SBS reference signatures (v2.03; https://github.com/Nik-Zainal-Group/signature.tools.lib/tree/master/data/RefSigSBS_v2.03).

SigProfilerMatrixGenerator (v1.2.9; [103]) was used to count the number of mutations on genic transcribed and untranscribed strands and an exact Poisson test was used to calculate significant strand bias for each mutation type. The bovine genome ARS-UCD1.2 was first installed, with a change made to the SigProfilerMatrixGenerator script ‘save_chrom_tsb_separate.py’ to include a list of bovine genome chromosomes. Canonical transcripts from Ensembl v98 and exome intervals list were used.

### Chromothripsis

We used the definition and scoring of chromothripsis as outlined in Vorinina et al. [54] to estimate the extent of these events in the canine and feline tumors from Sequenza CN segmentation results (see above). Regions positive for chromothripsis were defined as high confidence (10 or more CN state switches in 50 Mb), intermediate confidence (8 or 9 CN state switches in 50 Mb) or low confidence (6–7 CN state switches in 50 Mb). If positive, chromothripsis was further classified as canonical (2 or 3 CN states) or non-canonical (> 3 CN states). For chromosomes < 50 Mb, the number of required CN state switches was scaled accordingly and rounded to the nearest whole number. For example, if a chromosome is 30 Mb, a scaling factor of 0.6 (30/50 Mb) was applied to the definitions of high-confidence (6 or more CN state switches), intermediate-confidence (5 CN state switches), and low-confidence (4 CN state switches) regions of chromothripsis.

### Human urinary bladder cancer data

Mutation data from a TCGA human urinary bladder cancer study [22] were downloaded from the study’s cBioPortal website (https://www.cbioportal.org/study/summary?id=blca_tcga_pub_2017). Segmentation files from the same study were available from the International Cancer Genome Consortium (ICGC) Data Portal (https://dcc.icgc.org/) [104] for 278 donors; as we were only interested in comparing primary tumors, 1 metastatic tumor was excluded for our analysis. The reference genome for the study was GRCh37.

### Cross-species comparison

Orthologous genes and syntenic regions between human, canine, feline, and bovine genes and genomes were downloaded from Ensembl v98 [105]. For cross-species comparisons, only genes with a one-to-one orthologous relationship were included. Cancer Gene Consensus genes (v96) were downloaded from the COSMIC website (https://cancer.sanger.ac.uk/census) [106].

### Bracken fern extract and ptaquiloside purification

Fresh, uncurled fronds of bracken (fiddleheads), *Pteridium aquilinum* (L.) Khun. (Dennstaedtiaceae) were collected early in the growing season (mid-late May) from Baildon Moor, Bradford, West Yorkshire, UK, and processed within a few hours as previously reported [107]. Briefly, fiddleheads were frozen with liquid N_2_, powdered and macerated with acetone. The filtered extract was dried under N_2_ and then under vacuum to provide the acetone extract used (BFA) for cytotoxicity studies. The ethyl acetate extract (BFE) was obtained by concentrating an acetone extract under reduced pressure at 30 °C, diluting with water and partitioning several times with ethyl acetate. The extract was concentrated under reduced pressure at 40 °C then dried under vacuum. Ptaquiloside (PT) was isolated from an ethyl acetate extract by low-pressure column chromatography over silica gel as previously reported [107].

### Cell culture and chemosensitivity

KU-19–19 (RRID: CVCL_1344) are a human urinary bladder urothelial carcinoma (UC) cell line [108] with low/absent intrinsic APOBEC activity [109]. Cells used were authenticated as KU-19–19 by STR profiling and were verified as being mycoplasma-free [110]. Cells were maintained at low passage in antibiotic-free RPMI-1640 media (Sigma, R0883) supplemented with 10% fetal bovine serum and 2 mM L-glutamine. Chemosensitivity of KU-19–19 cells to purified BFA/BFE and PT was determined by MTT assay [111] following continuous daily cell exposure for 3, 7, 10, and 14 days (twofold serial dilution of BFA/BFE from 79.7 µg/ml to 0.156 µg/ml and twofold serial dilution of PT from 100 µM to 195 nM). KU-19–19 cells were seeded in 96-well cell culture plates at 1000 cells per well in 200µL of media and incubated for 24 h at 37 °C prior to BFA/BFE or PT exposure. BFA/BFE or PT at the required concentrations for testing were freshly prepared each day just before use by diluting in RPMI-1640 cell culture media from stock solutions in DMSO that were stored in single use aliquots at − 20 °C. All cells were exposed to a final DMSO concentration of 0.1% with percentage cell growth inhibition at each tested concentration of BFA/BFE or PT determined relative to ‘vehicle control’ (0.1% DMSO)-treated cells. Each day of cell treatment up to the day of the MTT assay, media was carefully removed from the wells and replaced with fresh culture media containing freshly diluted BFA/BFE or PT, or solvent. For the MTT assay, media was removed and fresh media containing MTT (0.5 mg/mL) was added to wells and cells were further incubated at 37 °C for 4 h to allow for formazan crystals to form. Crystals were dissolved in DMSO and absorbance readings at 540 nm were used to generate dose response curves for determination of IC_20_ and IC_50_ concentrations [111].

### KU-19–19 cell treatment with bracken fern extracts or purified ptaquiloside for mutational analyses

For DNA sequencing following the treatment of KU-19–19 cells with BFA, BFE or PT, experiments were scaled up from 96-well plates to 25cm^2^-cell culture flasks. KU-19–19 cells were seeded at 7.8 × 10^4^ cells per T25 flask in 5 ml of complete RPMI-1640 media 24 h prior to treatment. For the BFA/BFE experiments, KU-19–19 cells were treated daily for 3, 7, 10, and 14 days by media replacement with freshly diluted BFA/BFE at their IC_50_ and IC_20_ concentrations at each time point as pre-determined by MTT chemosensitivity assays. Chemosensitivity dose responses showed variation in their IC_50_ and IC_20_ concentrations depending on the number of days of cell treatment, and the concentrations used ranged from 3.1 to 79.7 µg/ml. For the PT experiments, KU-19–19 cells were treated daily for 3, 7, 10, and 14 days by media replacement with freshly diluted PT at a fixed concentration of 30 µM (mean IC_50_ concentration for these timepoints as determined by MTT chemosensitivity dose response curves), or 10 µM (approximate IC_20_ concentration) or 0.2 µM (‘non-cytotoxic’ concentration). For both experiments, solvent-exposed samples were used at the controls for each timepoint (designated by a ‘0’ in their sample name to indicate they had no BFA, BFE or PT exposure). At the endpoint of treatment for both experiments, cells were harvested from flasks by trypsinisation and cell pellets were washed twice with PBS to remove serum-containing media before ‘dry’ cell pellets were snap-frozen in liquid nitrogen. Genomic DNA was extracted from the cell pellets using the Purgene Cell Kit (Qiagen), according to the manufacturer’s instructions.

### Sequencing, read alignment and variant calling of the treated KU-19–19 cells

NanoSeq libraries were prepared from the KU-19–19 DNA following the duplex sequencing protocol as previously described [112]. A dilution of 0.2 fmols was taken for amplification and sequencing to 15x coverage (x: human haploid genome equivalents) using 150-bp paired-end reads on a NovaSeq 6000. The controls (KU_PTA_0_d3, d7, d10, and d14) were sequenced from these libraries by taking 5 fmols into amplification and sequencing to 15x coverage. Sequencing reads were aligned to the human reference genome (GRCh38, including decoys and HLA) using BWA-MEM (v0.7.17) [91] and processing of the data was performed as previously described [112]. Variant calling was done with the NanoSeq pipeline version 2 (March 24th, https://github.com/cancerit/NanoSeq), using the following parameters: -a 50 -b 0 -c 0 -d 2 -f 0.9 -i 0.2 -m 8 -n 3 -p 0 -q 60 -r 144 -v 0.01  -x 8 -z 15. For each time point, the treated sample was compared to the untreated control (KU_PTA_0) from the corresponding time point. Mutational signature extraction was performed as described above.

### Supplementary Information

**Additional file 1: Table S1. **Clinical details of the patients in the study.

**Additional file 2: Fig. S1. **Pathological presentation of invasive UC of the urinary bladder in a canine, feline, bovine and human.

**Additional file 3: Table S2. **Somatic mutations in urinary bladder canine UC.

**Additional file 4: Table S3. **Somatic mutations in urinary bladder feline UC.

**Additional file 5: Table S4. **Somatic mutations in urinary bladder bovine UC.

**Additional file 6: Fig. S2. **Recurrently mutated genes in human, canine and feline urinary bladder UC.

**Additional file 7: Table S5.** Significantly mutated genes (SMGs) in canine urinary bladder canine UC.

**Additional file 8: Table S6. **Significantly mutated genes (SMGs) in feline urinary bladder UC.

**Additional file 9: Fig. S3. **Recurrently mutated Cancer Gene Census (CGC) genes in bovine urinary bladder UC.

**Additional file 10: Fig. S4. **Indel mutational spectra, copy number and chromothripsis in canine urinary bladder UC.

**Additional file 11: Fig. S5. **Comparison of known mutational signatures with signatures found in bovine urinary bladder UC and in vitro in treated cell lines.

**Additional file 12: Fig. S6. **Single base substitution (SBS) mutation spectra showing the number of mutations on the genic transcribed and untranscribed strand.

**Additional file 13: Fig. S7. **Sequence context of single base pair deletions identified in bovine urinary bladder UC.

**Additional file 14: Fig. S8. **Bracken fern.

**Additional file 15: Table S7. **Mutational load and signature exposure in cells treated with bracken fern extract from acetone extraction.

**Additional file 16: Table S8. **Mutational load and signature exposure in cells treated with bracken fern extract from ethyl acetate extraction.

**Additional file 17: Fig. S9. **Mutational signatures in human bladder cancer cell lines after exposure to bracken fern extracts.

**Additional file 18: Fig. S10. **Single base substitution (SBS) mutation spectra and transcriptional strand bias in ptaquiloside-treated human bladder UC cells.

**Additional file 19: Fig. S11. **The indel mutation spectra from ptaquiloside-treated human bladder UC cells and bovine urinary bladder UC.

**Additional file 20: Fig. S12. **Sequence context single base pair deletions identified in ptaquiloside-treated human bladder UC cells.

**Additional file 21: Table S9. **Germline variants in urinary bladder canine UC.

**Additional file 22: Table S10. **Germline variants in urinary bladder feline UC.

**Additional file 23: Table S11. **Germline variants in urinary bladder bovine UC.

**Additional file 24: Fig. S13. **Somatic copy number alterations in bovine urinary bladder UC.

**Additional file 25: Fig. S14. **Chromothripsis-like events in feline urinary bladder UC.

**Additional file 26: Table S12. **Syntenic regions in human, canine and feline UC on recurrently amplified or deleted chromosomes.

**Additional file 27: Table S13. **Commonly amplified or deleted cancer genes from a comparison of focal amplifications and deletions across human, canine and feline urinary bladder UC.

**Additional file 28. **Review history.

## Data Availability

The dataset supporting the conclusions of this article is available in the European Nucleotide Archive repository (https://www.ebi.ac.uk/ena/browser/home), under the study accession ERP142199 [113]. Catalogs of known variants in the feline genome were obtained from the 99 Lives Cat Genome Consortium (v9, from 54 cat genomes) [88]. Catalogs of known variants in the canine genome were obtained from the National Human Genome Research Institute (NHGRI) Dog Genome Project [97]. Catalogs of known variants in the bovine genome were obtained from and the 1000 Bull Genomes Project [98].

## References

[1] Richters A, Aben KKH, Kiemeney L (2020). The global burden of urinary bladder cancer: an update. World J Urol.

[2] Siegel RL, Miller KD, Fuchs HE, Jemal A (2022). Cancer statistics, 2022. CA Cancer J Clin.

[3] Necchi A, Anichini A, Raggi D, Briganti A, Massa S, Lucianò R, Colecchia M, Giannatempo P, Mortarini R, Bianchi M (2018). Pembrolizumab as neoadjuvant therapy before radical cystectomy in patients with muscle-invasive urothelial bladder carcinoma (PURE-01): an open-label, single-arm, phase II study. J Clin Oncol.

[4] Witjes JA, Bruins HM, Cathomas R, Compérat EM, Cowan NC, Gakis G, Hernández V, Linares Espinós E, Lorch A, Neuzillet Y (2021). European Association of Urology Guidelines on muscle-invasive and metastatic bladder cancer: summary of the 2020 Guidelines. Eur Urol.

[5] Bajorin DF, Witjes JA, Gschwend JE, Schenker M, Valderrama BP, Tomita Y, Bamias A, Lebret T, Shariat SF, Park SH (2021). Adjuvant nivolumab versus placebo in muscle-invasive urothelial carcinoma. N Engl J Med.

[6] Miller KD, Siegel RL, Lin CC, Mariotto AB, Kramer JL, Rowland JH, Stein KD, Alteri R, Jemal A (2016). Cancer treatment and survivorship statistics, 2016. CA Cancer J Clin.

[7] Bhindi B, Frank I, Mason RJ, Tarrell RF, Thapa P, Cheville JC, Costello BA, Pagliaro LC, Karnes RJ, Thompson RH (2017). Oncologic outcomes for patients with residual cancer at cystectomy following neoadjuvant chemotherapy: a pathologic stage-matched analysis. Eur Urol.

[8] Puzio-Kuter AM, Castillo-Martin M, Kinkade CW, Wang X, Shen TH, Matos T, Shen MM, Cordon-Cardo C, Abate-Shen C (2009). Inactivation of p53 and Pten promotes invasive bladder cancer. Genes Dev.

[9] Fantini D, Glaser AP, Rimar KJ, Wang Y, Schipma M, Varghese N, Rademaker A, Behdad A, Yellapa A, Yu Y (2018). A Carcinogen-induced mouse model recapitulates the molecular alterations of human muscle invasive bladder cancer. Oncogene.

[10] De Vico G, Maiolino P, Conn PM (2008). Canine and feline models for cancer. Sourcebook of Models for Biomedical Research.

[11] Schiffman JD, Breen M (2015). Comparative oncology: what dogs and other species can teach us about humans with cancer. Philos Trans R Soc Lond B Biol Sci.

[12] Cannon CM (2015). Cats, cancer and comparative oncology. Vet Sci.

[13] Nance RL, Sajib AM, Smith BF. Canine models of human cancer: bridging the gap to improve precision medicine. Prog Mol Biol Transl Sci. 2022;189:67–99. 10.1016/bs.pmbts.2021.12.003 35595353

[14] Meuten DJ, Meuten TLK (2016). Tumors of the urinary system. Tumors in Domestic Animals.

[15] Pinto C, Januário T, Geraldes M, Machado J, Lauren D, Smith B, Robinson R, Acamovic T, Stewart CS, Pennycott TW (2004). Bovine enzootic haematuria on Sao Miguel Island, Azores. oisonous Plants and Related Toxins.

[16] D'Mello JF. Handbook of plant and fungal toxicants. Boca Raton: CRC press; 1997.

[17] Carvalho T, Pinto C, Peleteiro MC (2006). Urinary bladder lesions in bovine enzootic haematuria. J Comp Pathol.

[18] Evans IA, Jones RS, Mainwaring-Burton R (1972). Passage of bracken fern toxicity into milk. Nature.

[19] Francesco B, Giorgio B, Rosario N, Saverio RF, Francesco DG, Romano M, Adriano S, Cinzia R, Antonio T, Franco R (2011). A new, very sensitive method of assessment of ptaquiloside, the major bracken carcinogen in the milk of farm animals. Food Chem.

[20] Clauson-Kaas F, Jensen PH, Jacobsen OS, Juhler RK, Hansen HC (2014). The naturally occurring carcinogen ptaquiloside is present in groundwater below bracken vegetation. Environ Toxicol Chem.

[21] Rasmussen LH (2021). Presence of the carcinogen ptaquiloside in fern-based food products and traditional medicine: four cases of human exposure. Curr Res Food Sci.

[22] Robertson AG, Kim J, Al-Ahmadie H, Bellmunt J, Guo G, Cherniack AD, Hinoue T, Laird PW, Hoadley KA, Akbani R (2017). Comprehensive molecular characterization of muscle-invasive bladder cancer. Cell.

[23] Network CGAR (2014). Comprehensive molecular characterization of urothelial bladder carcinoma. Nature.

[24] Martincorena I, Campbell PJ (2015). Somatic mutation in cancer and normal cells. Science.

[25] Mochizuki H, Kennedy K, Shapiro SG, Breen M (2015). BRAF mutations in canine cancers. PLoS One.

[26] Decker B, Parker HG, Dhawan D, Kwon EM, Karlins E, Davis BW, Ramos-Vara JA, Bonney PL, McNiel EA, Knapp DW, Ostrander EA (2015). Homologous mutation to human BRAF V600E is common in naturally occurring canine bladder cancer–evidence for a relevant model system and urine-based diagnostic test. Mol Cancer Res.

[27] Gedon J, Kehl A, Aupperle-Lellbach H, von Bomhard W, Schmidt JM (2022). BRAF mutation status and its prognostic significance in 79 canine urothelial carcinomas: a retrospective study (2006–2019). Vet Comp Oncol.

[28] Cronise KE, Das S, Hernandez BG, Regan DP, Dailey DD, McGeachan RI, Lana SE, Page RL, Gustafson DL, Duval DL (2022). Characterizing the molecular and immune landscape of canine bladder cancer. Vet Comp Oncol.

[29] Norris AM, Laing EJ, Valli VE, Withrow SJ, Macy DW, Ogilvie GK, Tomlinson J, McCaw D, Pidgeon G, Jacobs RM (1992). Canine bladder and urethral tumors: a retrospective study of 115 cases (1980–1985). J Vet Intern Med.

[30] Knapp DW, Ramos-Vara JA, Moore GE, Dhawan D, Bonney PL, Young KE (2014). Urinary bladder cancer in dogs, a naturally occurring model for cancer biology and drug development. Ilar j.

[31] Canisius S, Martens JW, Wessels LF (2016). A novel independence test for somatic alterations in cancer shows that biology drives mutual exclusivity but chance explains most co-occurrence. Genome Biol.

[32] Thomas R, Wiley CA, Droste EL, Robertson J, Inman BA, Breen M (2023). Whole exome sequencing analysis of canine urothelial carcinomas without BRAF V595E mutation: Short in-frame deletions in BRAF and MAP2K1 suggest alternative mechanisms for MAPK pathway disruption. PLoS Genet.

[33] Prakash AS, Pereira TN, Smith BL, Shaw G, Seawright AA (1996). Mechanism of bracken fern carcinogenesis: evidence for H-ras activation via initial adenine alkylation by ptaquiloside. Nat Toxins.

[34] Shahin M, Moore MR, Worrall S, Smith BL, Seawright AA, Prakash AS (1998). H-ras activation is an early event in the ptaquiloside-induced carcinogenesis: comparison of acute and chronic toxicity in rats. Biochem Biophys Res Commun.

[35] Tate JG, Bamford S, Jubb HC, Sondka Z, Beare DM, Bindal N, Boutselakis H, Cole CG, Creatore C, Dawson E (2018). COSMIC: the Catalogue Of Somatic Mutations In Cancer. Nucleic Acids Res.

[36] Hodgson A, Vesprini D, Liu SK, Xu B, Downes MR (2020). Correlation of mismatch repair protein deficiency, PD-L1 and CD8 expression in high-grade urothelial carcinoma of the bladder. J Clin Pathol.

[37] Fraune C, Simon R, Hube-Magg C, Makrypidi-Fraune G, Kähler C, Kluth M, Höflmayer D, Büscheck F, Dum D, Luebke AM (2020). MMR deficiency in urothelial carcinoma of the bladder presents with temporal and spatial homogeneity throughout the tumor mass. Urol Oncol.

[38] Sobrino-Reig E, Meizoso T, García J, Varillas-Delgado D, Martin YB (2021). Morphological predictors for microsatellite instability in urothelial carcinoma. Diagn Pathol.

[39] Mohamedali R, Adhya AK, Mandal S, Mitra S (2022). Expression of mismatch repair proteins in urothelial carcinoma of the urinary bladder. Indian J Cancer.

[40] Inanaga S, Igase M, Sakai Y, Tanabe M, Shimonohara N, Itamoto K, Nakaichi M, Mizuno T (2021). Mismatch repair deficiency in canine neoplasms. Vet Pathol.

[41] Gori K, Baez-Ortega A. sigfit: flexible Bayesian inference of mutational signatures. bioRxiv 2020:372896.

[42] Alexandrov LB, Kim J, Haradhvala NJ, Huang MN, Tian Ng AW, Wu Y, Boot A, Covington KR, Gordenin DA, Bergstrom EN (2020). The repertoire of mutational signatures in human cancer. Nature.

[43] Alexandrov LB, Jones PH, Wedge DC, Sale JE, Campbell PJ, Nik-Zainal S, Stratton MR (2015). Clock-like mutational processes in human somatic cells. Nat Genet.

[44] Alexandrov LB, Nik-Zainal S, Wedge DC, Aparicio SA, Behjati S, Biankin AV, Bignell GR, Bolli N, Borg A, Børresen-Dale AL (2013). Signatures of mutational processes in human cancer. Nature.

[45] Degasperi A, Amarante TD, Czarnecki J, Shooter S, Zou X, Glodzik D, Morganella S, Nanda AS, Badja C, Koh G (2020). A practical framework and online tool for mutational signature analyses show inter-tissue variation and driver dependencies. Nat Cancer.

[46] Potter DM, Baird MS (2000). Carcinogenic effects of ptaquiloside in bracken fern and related compounds. Br J Cancer.

[47] Nassar AH, Abou Alaiwi S, AlDubayan SH, Moore N, Mouw KW, Kwiatkowski DJ, Choueiri TK, Curran C, Berchuck JE, Harshman LC (2020). Prevalence of pathogenic germline cancer risk variants in high-risk urothelial carcinoma. Genet Med.

[48] Carlo MI, Ravichandran V, Srinavasan P, Bandlamudi C, Kemel Y, Ceyhan-Birsoy O, Mukherjee S, Mandelker D, Chaim J, Knezevic A (2020). Cancer susceptibility mutations in patients with urothelial malignancies. J Clin Oncol.

[49] Shapiro SG, Raghunath S, Williams C, Motsinger-Reif AA, Cullen JM, Liu T, Albertson D, Ruvolo M, Bergstrom Lucas A, Jin J (2015). Canine urothelial carcinoma: genomically aberrant and comparatively relevant. Chromosome Res.

[50] Ciriello G, Miller ML, Aksoy BA, Senbabaoglu Y, Schultz N, Sander C (2013). Emerging landscape of oncogenic signatures across human cancers. Nat Genet.

[51] Hirono I, Ogino H, Fujimoto M, Yamada K, Yoshida Y, Ikagawa M, Okumura M (1987). Induction of tumors in ACI rats given a diet containing ptaquiloside, a bracken carcinogen. J Natl Cancer Inst.

[52] Morrison CD, Liu P, Woloszynska-Read A, Zhang J, Luo W, Qin M, Bshara W, Conroy JM, Sabatini L, Vedell P (2014). Whole-genome sequencing identifies genomic heterogeneity at a nucleotide and chromosomal level in bladder cancer. Proc Natl Acad Sci U S A.

[53] Cortés-Ciriano I, Lee JJK, Xi R, Jain D, Jung YL, Yang L, Gordenin D, Klimczak LJ, Zhang CZ, Pellman DS (2020). Comprehensive analysis of chromothripsis in 2,658 human cancers using whole-genome sequencing. Nat Genet.

[54] Voronina N, Wong JKL, Hübschmann D, Hlevnjak M, Uhrig S, Heilig CE, Horak P, Kreutzfeldt S, Mock A, Stenzinger A (2020). The landscape of chromothripsis across adult cancer types. Nat Commun.

[55] Diskin SJ, Eck T, Greshock J, Mosse YP, Naylor T, Stoeckert CJ, Weber BL, Maris JM, Grant GR (2006). STAC: a method for testing the significance of DNA copy number aberrations across multiple array-CGH experiments. Genome Res.

[56] Gui Y, Guo G, Huang Y, Hu X, Tang A, Gao S, Wu R, Chen C, Li X, Zhou L (2011). Frequent mutations of chromatin remodeling genes in transitional cell carcinoma of the bladder. Nat Genet.

[57] Williams SV, Taylor C, Platt F, Hurst CC, Aveyard J, Knowles MA (2010). Mutation and homozygous deletion of ARHGEF10 in bladder cancer; a candidate tumour suppressor gene at 8p23.3. Cancer Genet.

[58] Knapp DW, Dhawan D, Ramos-Vara JA, Ratliff TL, Cresswell GM, Utturkar S, Sommer BC, Fulkerson CM, Hahn NM (2019). Naturally-occurring invasive urothelial carcinoma in dogs, a unique model to drive advances in managing muscle invasive bladder cancer in humans. Front Oncol.

[59] Dhawan D, Paoloni M, Shukradas S, Choudhury DR, Craig BA, Ramos-Vara JA, Hahn N, Bonney PL, Khanna C, Knapp DW (2015). Comparative gene expression analyses identify luminal and basal subtypes of canine invasive urothelial carcinoma that mimic patterns in human invasive bladder cancer. PLoS One.

[60] Maeda S, Tomiyasu H, Tsuboi M, Inoue A, Ishihara G, Uchikai T, Chambers JK, Uchida K, Yonezawa T, Matsuki N (2018). Comprehensive gene expression analysis of canine invasive urothelial bladder carcinoma by RNA-Seq. BMC Cancer.

[61] Dhawan D, Hahn NM, Ramos-Vara JA, Knapp DW (2018). Naturally-occurring canine invasive urothelial carcinoma harbors luminal and basal transcriptional subtypes found in human muscle invasive bladder cancer. PLoS Genet.

[62] Parker HG, Dhawan D, Harris AC, Ramos-Vara JA, Davis BW, Knapp DW, Ostrander EA (2020). RNAseq expression patterns of canine invasive urothelial carcinoma reveal two distinct tumor clusters and shared regions of dysregulation with human bladder tumors. BMC Cancer.

[63] Wimberly HC, Lewis RM (1979). Transitional cell carcinoma in the domestic cat. Vet Pathol.

[64] Shida T, Yamada T, Maruo T, Ishida T, Kawamura H, Takeda H, Sugiyama H, Ishikawa T, Ito T, Madarame H (2010). A retrospective study in 1,070 feline tumor cases of Japan. J Japan Vet Cancer Soc.

[65] Knapp D, McMillan S, Withrow S, Vail D (2013). Tumors of the urinary system in Withrow and MacEwen's Small Animal Clinical Oncology.

[66] Ludwig L, Dobromylskyj M, Wood GA, Van der Weyden L (2022). Feline oncogenomics: what do we know about the genetics of cancer in domestic cats?. Vet Sci.

[67] Gyles C (2016). One Medicine, One Health. One World Can Vet J.

[68] Brown CJ, Lain S, Verma CS, Fersht AR, Lane DP (2009). Awakening guardian angels: drugging the p53 pathway. Nat Rev Cancer.

[69] Sdek P, Ying H, Chang DL, Qiu W, Zheng H, Touitou R, Allday MJ, Xiao ZX (2005). MDM2 promotes proteasome-dependent ubiquitin-independent degradation of retinoblastoma protein. Mol Cell.

[70] Plimack ER, Dunbrack RL, Brennan TA, Andrake MD, Zhou Y, Serebriiskii IG, Slifker M, Alpaugh K, Dulaimi E, Palma N (2015). Defects in DNA repair genes predict response to neoadjuvant cisplatin-based chemotherapy in muscle-invasive bladder cancer. Eur Urol.

[71] Mochizuki H, Shapiro SG, Breen M (2015). Detection of BRAF mutation in urine DNA as a molecular diagnostic for canine urothelial and prostatic carcinoma. PLoS One.

[72] Stoehr R, Brinkmann A, Filbeck T, Gamper C, Wild P, Blaszyk H, Hofstaedter F, Knuechel R, Hartmann A (2004). No evidence for mutation of B-RAF in urothelial carcinomas of the bladder and upper urinary tract. Oncol Rep.

[73] Boulalas I, Zaravinos A, Delakas D, Spandidos DA (2009). Mutational analysis of the BRAF gene in transitional cell carcinoma of the bladder. Int J Biol Markers.

[74] Win AK, Lindor NM, Young JP, Macrae FA, Young GP, Williamson E, Parry S, Goldblatt J, Lipton L, Winship I (2012). Risks of primary extracolonic cancers following colorectal cancer in lynch syndrome. J Natl Cancer Inst.

[75] van der Post RS, Kiemeney LA, Ligtenberg MJ, Witjes JA, Hulsbergen-van de Kaa CA, Bodmer D, Schaap L, Kets CM, van Krieken JH, Hoogerbrugge N. Risk of urothelial bladder cancer in Lynch syndrome is increased, in particular among MSH2 mutation carriers. J Med Genet. 2010;47:464–70. 10.1136/jmg.2010.076992 PMC2991077 20591884

[76] Audenet F, Isharwal S, Cha EK, Donoghue MTA, Drill EN, Ostrovnaya I, Pietzak EJ, Sfakianos JP, Bagrodia A, Murugan P (2019). Clonal relatedness and mutational differences between upper tract and bladder urothelial carcinoma. Clin Cancer Res.

[77] Yang Y, Jain RK, Glenn ST, Xu B, Singh PK, Wei L, Hu Q, Long M, Hutson N, Wang J (2020). Complete response to anti-PD-L1 antibody in a metastatic bladder cancer associated with novel MSH4 mutation and microsatellite instability. J Immunother Cancer.

[78] Shahin M, Smith BL, Prakash AS (1999). Bracken carcinogens in the human diet. Mutat Res.

[79] Alonso-Amelot M, Avendaño M (2002). Human Carcinogenesis and Bracken Fern: A Review of the Evidence. Curr Med Chem.

[80] Gil da Costa RM, Bastos MM, Oliveira PA, Lopes C (2012). Bracken-associated human and animal health hazards: chemical, biological and pathological evidence. J Hazard Mater.

[81] Nagaraja V, Eslick GD (2015). The role of the bracken fern in upper gastrointestinal tract malignancies: a systematic review of the evidence. Am J Cancer Epidemiol Prevent.

[82] Rasmussen LH, Kroghsbo S, Frisvad JC, Hansen HC (2003). Occurrence of the carcinogenic Bracken constituent ptaquiloside in fronds, topsoils and organic soil layers in Denmark. Chemosphere.

[83] Kisielius V, Drejer M, Dornhoff JK, Mrkajic NS, Lindqvist DN, Hansen HCB, Rasmussen LH (2022). Occurrence and stability of ptesculentoside, caudatoside and ptaquiloside in surface waters. Environ Sci Process Impacts.

[84] van der Hoeven JCM, Lagerweij WJ, Posthumus MA, van Veldhuizen A, Holterman HAJ (1983). Aquilide A, a new mutagenic compound isolated from bracken fern ( Pteridium aquilinum (L.) Kuhn). Carcinogenesis.

[85] Povey AC, Potter D, O'Connor PJ (1996). 32P-post-labelling analysis of DNA adducts formed in the upper gastrointestinal tissue of mice fed bracken extract or bracken spores. Br J Cancer.

[86] Smith BL, Seawright AA, Ng JC, Hertle AT, Thomson JA, Bostock PD (1994). Concentration of ptaquiloside, a major carcinogen in bracken fern (Pteridium spp.), from eastern Australia and from a cultivated worldwide collection held in Sydney, Australia. Nat Toxins.

[87] Hoeppner MP, Lundquist A, Pirun M, Meadows JR, Zamani N, Johnson J, Sundström G, Cook A, FitzGerald MG, Swofford R (2014). An improved canine genome and a comprehensive catalogue of coding genes and non-coding transcripts. PLoS One.

[88] Buckley RM, Davis BW, Brashear WA, Farias FHG, Kuroki K, Graves T, Hillier LW, Kremitzki M, Li G, Middleton RP (2020). A new domestic cat genome assembly based on long sequence reads empowers feline genomic medicine and identifies a novel gene for dwarfism. PLoS Genet.

[89] Rosen BD, Bickhart DM, Schnabel RD, Koren S, Elsik CG, Tseng E, Rowan TN, Low WY, Zimin A, Couldrey C (2020). De novo assembly of the cattle reference genome with single-molecule sequencing. Gigascience.

[90] Wong K, Ludwig L, Krijgsman O, Adams DJ, Wood GA, van der Weyden L (2021). Comparison of the oncogenomic landscape of canine and feline hemangiosarcoma shows novel parallels with human angiosarcoma. Dis Model Mech.

[91] Li H. Aligning sequence reads, clone sequences and assembly contigs with BWA-MEM. 2013;arXiv:1303.3997.

[92] Tischler G, Leonard S (2014). biobambam: tools for read pair collation based algorithms on BAM files. Source Code Biol Med.

[93] Cibulskis K, Lawrence MS, Carter SL, Sivachenko A, Jaffe D, Sougnez C, Gabriel S, Meyerson M, Lander ES, Getz G (2013). Sensitive detection of somatic point mutations in impure and heterogeneous cancer samples. Nat Biotechnol.

[94] Wei L, Liu LT, Conroy JR, Hu Q, Conroy JM, Morrison CD, Johnson CS, Wang J, Liu S (2015). MAC: identifying and correcting annotation for multi-nucleotide variations. BMC Genomics.

[95] Kim S, Scheffler K, Halpern AL, Bekritsky MA, Noh E, Källberg M, Chen X, Kim Y, Beyter D, Krusche P, Saunders CT (2018). Strelka2: fast and accurate calling of germline and somatic variants. Nat Methods.

[96] McLaren W, Gil L, Hunt SE, Riat HS, Ritchie GR, Thormann A, Flicek P, Cunningham F (2016). The Ensembl Variant Effect Predictor. Genome Biol.

[97] Plassais J, Kim J, Davis BW, Karyadi DM, Hogan AN, Harris AC, Decker B, Parker HG, Ostrander EA (2019). Whole genome sequencing of canids reveals genomic regions under selection and variants influencing morphology. Nat Commun.

[98] Hayes BJ, Daetwyler HD (2019). 1000 Bull Genomes Project to map simple and complex genetic traits in cattle: applications and outcomes. Annu Rev Anim Biosci.

[99] DePristo MA, Banks E, Poplin R, Garimella KV, Maguire JR, Hartl C, Philippakis AA, del Angel G, Rivas MA, Hanna M (2011). A framework for variation discovery and genotyping using next-generation DNA sequencing data. Nat Genet.

[100] Favero F, Joshi T, Marquard AM, Birkbak NJ, Krzystanek M, Li Q, Szallasi Z, Eklund AC (2015). Sequenza: allele-specific copy number and mutation profiles from tumor sequencing data. Ann Oncol.

[101] Martincorena I, Raine KM, Gerstung M, Dawson KJ, Haase K, Van Loo P, Davies H, Stratton MR, Campbell PJ (2017). Universal patterns of selection in cancer and somatic tissues. Cell.

[102] Dees ND, Zhang Q, Kandoth C, Wendl MC, Schierding W, Koboldt DC, Mooney TB, Callaway MB, Dooling D, Mardis ER (2012). MuSiC: identifying mutational significance in cancer genomes. Genome Res.

[103] Bergstrom EN, Huang MN, Mahto U, Barnes M, Stratton MR, Rozen SG, Alexandrov LB (2019). SigProfilerMatrixGenerator: a tool for visualizing and exploring patterns of small mutational events. BMC Genomics.

[104] Zhang J, Bajari R, Andric D, Gerthoffert F, Lepsa A, Nahal-Bose H, Stein LD, Ferretti V (2019). The International Cancer Genome Consortium Data Portal. Nat Biotechnol.

[105] Cunningham F, Allen JE, Allen J, Alvarez-Jarreta J, Amode MR, Armean IM, Austine-Orimoloye O, Azov AG, Barnes I, Bennett R (2022). Ensembl 2022. Nucleic Acids Res.

[106] Futreal PA, Coin L, Marshall M, Down T, Hubbard T, Wooster R, Rahman N, Stratton MR (2004). A census of human cancer genes. Nat Rev Cancer.

[107] Williams C, Allison SJ, Phillips RM, Linley PA, Wright CW (2021). An efficient method for the isolation of toxins from Pteridium aquilinum and evaluation of ptaquiloside against cancer and non-cancer cells. Planta Med.

[108] Tachibana M, Miyakawa A, Tazaki H, Nakamura K, Kubo A, Hata J, Nishi T, Amano Y (1995). Autocrine growth of transitional cell carcinoma of the bladder induced by granulocyte-colony stimulating factor. Cancer Res.

[109] Glaser AP, Fantini D, Wang Y, Yu Y, Rimar KJ, Podojil JR, Miller SD, Meeks JJ (2018). APOBEC-mediated mutagenesis in urothelial carcinoma is associated with improved survival, mutations in DNA damage response genes, and immune response. Oncotarget.

[110] Burns JE, Hurst CD, Knowles MA, Phillips RM, Allison SJ (2021). The Warburg effect as a therapeutic target for bladder cancers and intratumoral heterogeneity in associated molecular targets. Cancer Sci.

[111] Allison SJ, Bryk J, Clemett CJ, Faulkner RA, Ginger M, Griffiths HBS, Harmer J, Jane Owen-Lynch P, Pinder E, Wurdak H (2021). Self-assembly of an anion receptor with metal-dependent kinase inhibition and potent in vitro anti-cancer properties. Nat Commun.

[112] Abascal F, Harvey LMR, Mitchell E, Lawson ARJ, Lensing SV, Ellis P, Russell AJC, Alcantara RE, Baez-Ortega A, Wang Y (2021). Somatic mutation landscapes at single-molecule resolution. Nature.

[113] Wong K, Abascal F, Ludwig L, Aupperle-Lellbach H, Grassinger J, Wright CW, Allison SJ, Pinder E, Phillips RM, Romero LP, et al. Cross species analysis of the genomic landscape of canine, feline, bovine and human bladder urothelial carcinoma reveals a novel mutational signature associated with bracken fern consumption. European Nucleotide Archive: https://www.ebi.ac.uk/ena/browser/text-search?query=ERP142199; 2023.

