# Supplementary material for: Cross-species oncogenomics offers insight into human muscle-invasive bladder cancer
Source: Genome Biol. 2023 Aug 28;24:191. doi: 10.1186/s13059-023-03026-4 (PMC10464500; doi:10.1186/s13059-023-03026-4)
Supplement: Supplementary file 2 — Additional file 2: Fig. S1. Pathological presentation of invasive UC of the urinary bladder in a canine, feline, bovine and human. [file 13059_2023_3026_MOESM2_ESM.pdf]

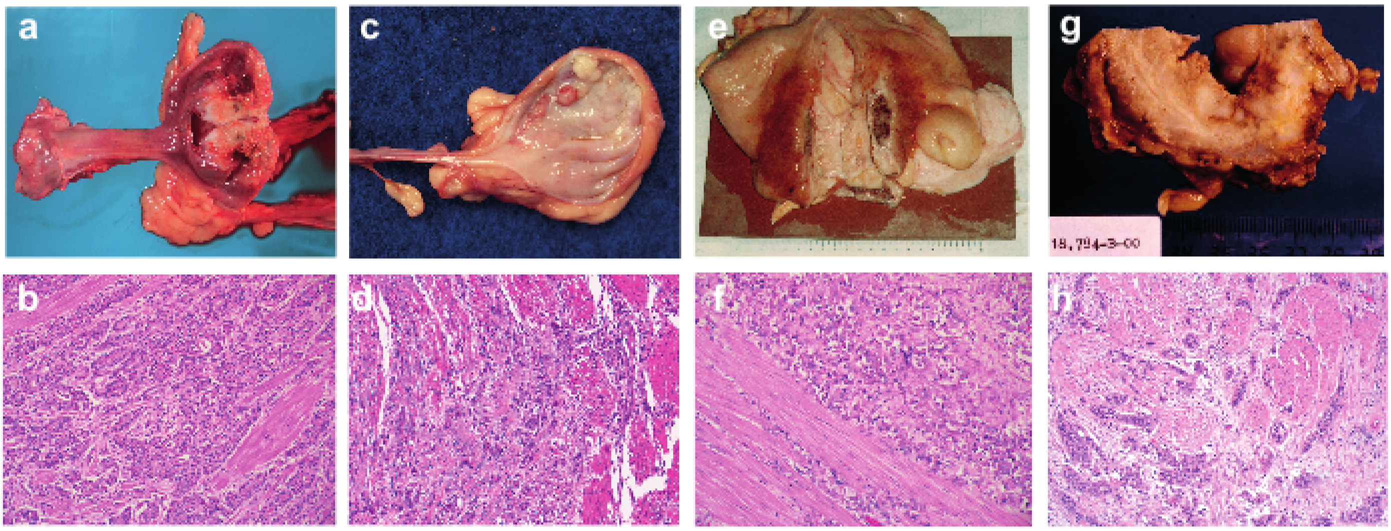

Fig. S1. Pathological presentation of UC of the urinary bladder in a canine, feline, bovine and human. Macroscopic image of an invasive UC from an **a,** 7-year old female neutered Great Dane dog, **c,** 18-year old female neutered Domestic shorthair cat, **e,** 4-year old female Holstein-Friesian cow and **g,** 73-year old male. Histopathological image of a H&E-stained section (x20 magnification) of invasive UC from an **b,** 7-year old female dog of unknown neutering status and breed, **d,** 5-year old male neutered Domestic shorthair cat, **f,** female Holstein-Friesian cow of unknown age and **h,** 73-year old male. Photo credits: Dr. Jeff Caswell, Department of Pathobiology, University of Guelph, Canada (**a, c**) and Professor Antonio Lopez-Beltran, Champalimaud Clinical Center, Portugal (**g, h**).
