# Supplementary material for: Cross-species oncogenomics offers insight into human muscle-invasive bladder cancer
Source: Genome Biol. 2023 Aug 28;24:191. doi: 10.1186/s13059-023-03026-4 (PMC10464500; doi:10.1186/s13059-023-03026-4)
Supplement: Supplementary file 6 — Additional file 6: Fig. S2. Recurrently mutated genes in human, canine and feline urinary bladder UC. [file 13059_2023_3026_MOESM6_ESM.pdf]

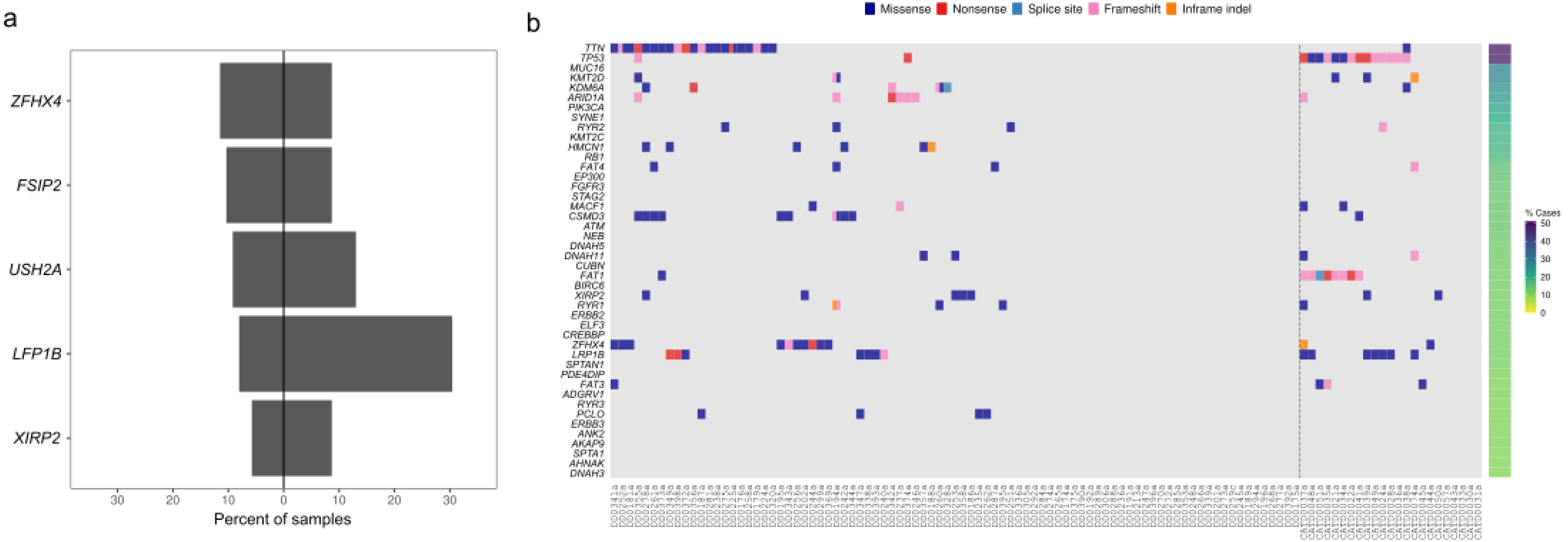

Fig. S2. Recurrently mutated genes in human, canine and feline urinary bladder UC. a, A comparison of genes that were mutated in at least 5% of samples in both the canine (left) and feline cohort (right). Genes shown have a one-to-one orthologous relationship between the canine and feline gene. **b,** A comparison of genes mutated in at least 10% of human UC samples and their mutation status in the canine and feline UC samples. Gene symbols shown are human gene symbols, if different or not assigned in the dog or cat Ensembl gene build. The human MIBC data from 412 samples were obtained from the TCGA and the genes mutated are represented as a percentage of total cases.
