# Supplementary material for: Cross-species oncogenomics offers insight into human muscle-invasive bladder cancer
Source: Genome Biol. 2023 Aug 28;24:191. doi: 10.1186/s13059-023-03026-4 (PMC10464500; doi:10.1186/s13059-023-03026-4)
Supplement: Supplementary file 7 — Additional file 7: Table S5. Significantly mutated genes (SMGs) in canine urinary bladder canine UC. [file 13059_2023_3026_MOESM7_ESM.pdf]

Table S5a. Significantly mutated genes (SMGs) in canine urinary bladder canine UC.

**Ensembl ID**	**Gene**	***q* (subs)**	***q* (global)**
ENSCAFG00000003907	*BRAF*	0.00E+00	0.00E+00
ENSCAFG00000019028	*CDH12*	4.08E-02	3.41E-02
ENSCAFG00000012314	*ARID1A*	9.96E-01	4.73E-02
ENSCAFG00000014589	*KDM6A*	9.96E-01	8.36E-02

SMGs identified using dNdScv. A gene was considered a SMG if the *q* value based on substitutions only (subs) or the *q* value based on all mutation types (global) was < 0.1.

Table S5b. Significantly mutated genes in canine urinary bladder canine UC.

**Ensembl ID**	**Gene**	**FDR CT**
ENSCAFG00000003907	*BRAF*	0.00E+00
ENSCAFG00000019028	*CDH12*	3.92E-06
ENSCAFG00000017775	*GPRASP1*	1.12E-04
ENSCAFG00000004870	*PCDH17*	1.08E-03
ENSCAFG00000012314	*ARID1A*	1.72E-03
ENSCAFG00000004944	*PCDH9*	1.72E-03
ENSCAFG00000000786	*CSMD3*	1.72E-03
ENSCAFG00000014589	*KDM6A*	8.25E-03
ENSCAFG00000001862	*ZNF804B*	8.25E-03

SMGs identified using MuSiC2 with FDR CT < 0.01, where CT is the convolution test.
