# Supplementary material for: Cross-species oncogenomics offers insight into human muscle-invasive bladder cancer
Source: Genome Biol. 2023 Aug 28;24:191. doi: 10.1186/s13059-023-03026-4 (PMC10464500; doi:10.1186/s13059-023-03026-4)
Supplement: Supplementary file 8 — Additional file 8: Table S6. Significantly mutated genes (SMGs) in feline urinary bladder UC. [file 13059_2023_3026_MOESM8_ESM.pdf]

Table S6a. Significantly mutated genes (SMGs) in feline urinary bladder UC.

Ensembl ID	Gene	*q* (subs)	*q* (global)
ENSFCAG00000009623	*TP53*	2.45E-06	0
ENSFCAG00000015711	*BAP1*	1.80E-01	7.83E-05
ENSFCAG00000025616	*FAT1*	3.42E-02	2.96E-04
ENSFCAG00000015718	*PBRM1*	8.49E-03	8.94E-03
ENSFCAG00000026584	*LRP1B*	8.49E-03	1.59E-02
ENSFCAG00000001953	*JAK1*	7.32E-03	3.68E-02
ENSFCAG00000037994	*NRAS*	1.64E-02	1.37E-01
ENSFCAG00000022826	*CDKN2A*	3.39E-02	2.81E-01

SMGs identified using dNdScv. A gene was considered a SMG if the *q* value based on substitutions only (subs) or the *q* value based on all mutation types (global) was < 0.1.

Table S6b. Significantly mutated genes in feline urinary bladder UC.

Ensembl ID	Gene	FDR CT
ENSFCAG00000009623	*TP53*	0.00E+00
ENSFCAG00000025616	*FAT1*	6.28E-07
ENSFCAG00000015711	*BAP1*	2.55E-06
ENSFCAG00000019287	*SETD2*	1.77E-04
ENSFCAG00000026584	*LRP1B*	5.12E-04
ENSFCAG00000037994	*NRAS*	1.60E-03
ENSFCAG00000001953	*JAK1*	2.02E-03

SMGs identified using MuSiC2 with FDR CT < 0.01, where CT is the convolution test.
