# Supplementary material for: Cross-species oncogenomics offers insight into human muscle-invasive bladder cancer
Source: Genome Biol. 2023 Aug 28;24:191. doi: 10.1186/s13059-023-03026-4 (PMC10464500; doi:10.1186/s13059-023-03026-4)
Supplement: Supplementary file 9 — Additional file 9: Fig. S3. Recurrently mutated Cancer Gene Census (CGC) genes in bovine urinary bladder UC. [file 13059_2023_3026_MOESM9_ESM.pdf]

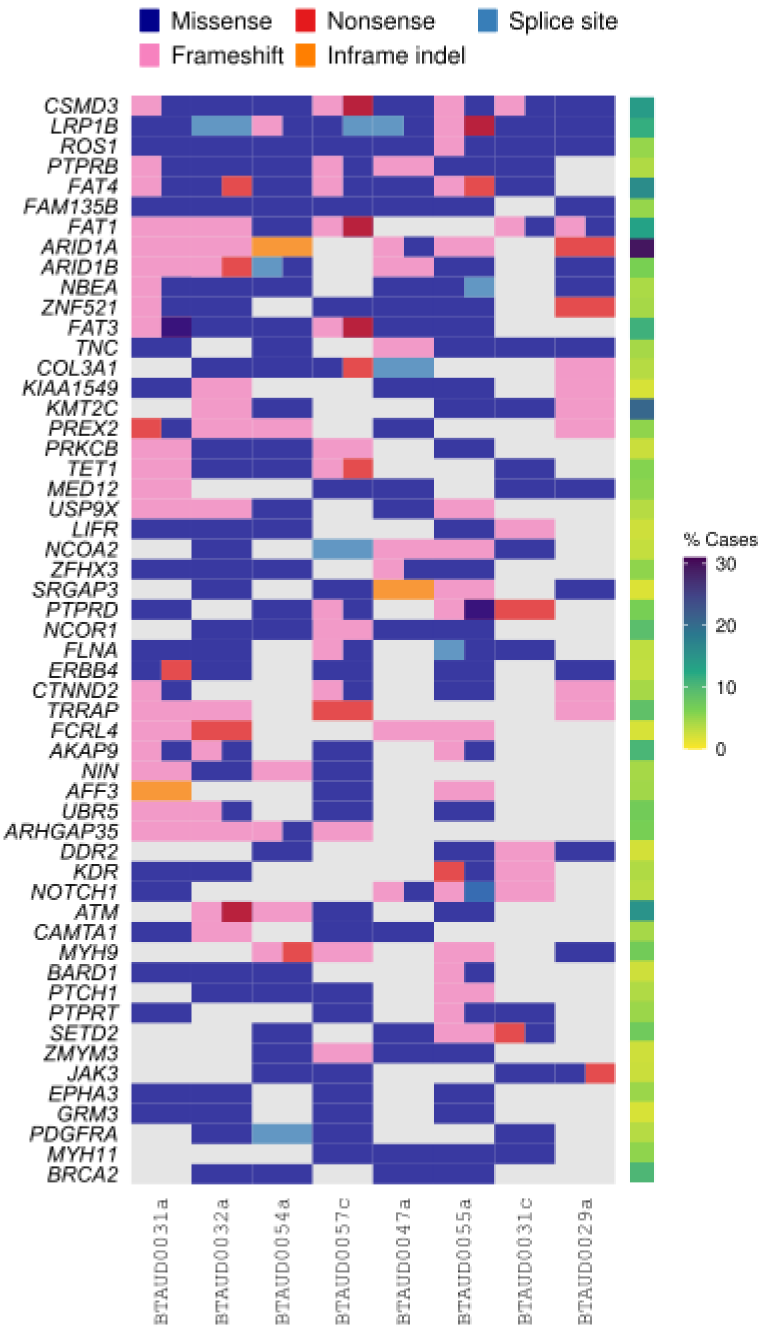

Fig. S3. Recurrently mutated Cancer Gene Census (CGC) genes in bovine urinary bladder UC. Shown are COSMIC CGC genes mutated in at least 4 bovine UC cases (left), and the proportion of human UC cases with mutations in these genes (right). Genes shown are those that had a one-to-one orthologous relationship between the human and bovine gene.
