# Supplementary material for: Cross-species oncogenomics offers insight into human muscle-invasive bladder cancer
Source: Genome Biol. 2023 Aug 28;24:191. doi: 10.1186/s13059-023-03026-4 (PMC10464500; doi:10.1186/s13059-023-03026-4)
Supplement: Supplementary file 10 — Additional file 10: Fig. S4. Indel mutational spectra, copy number and chromothripsis in canine urinary bladder UC. [file 13059_2023_3026_MOESM10_ESM.pdf]

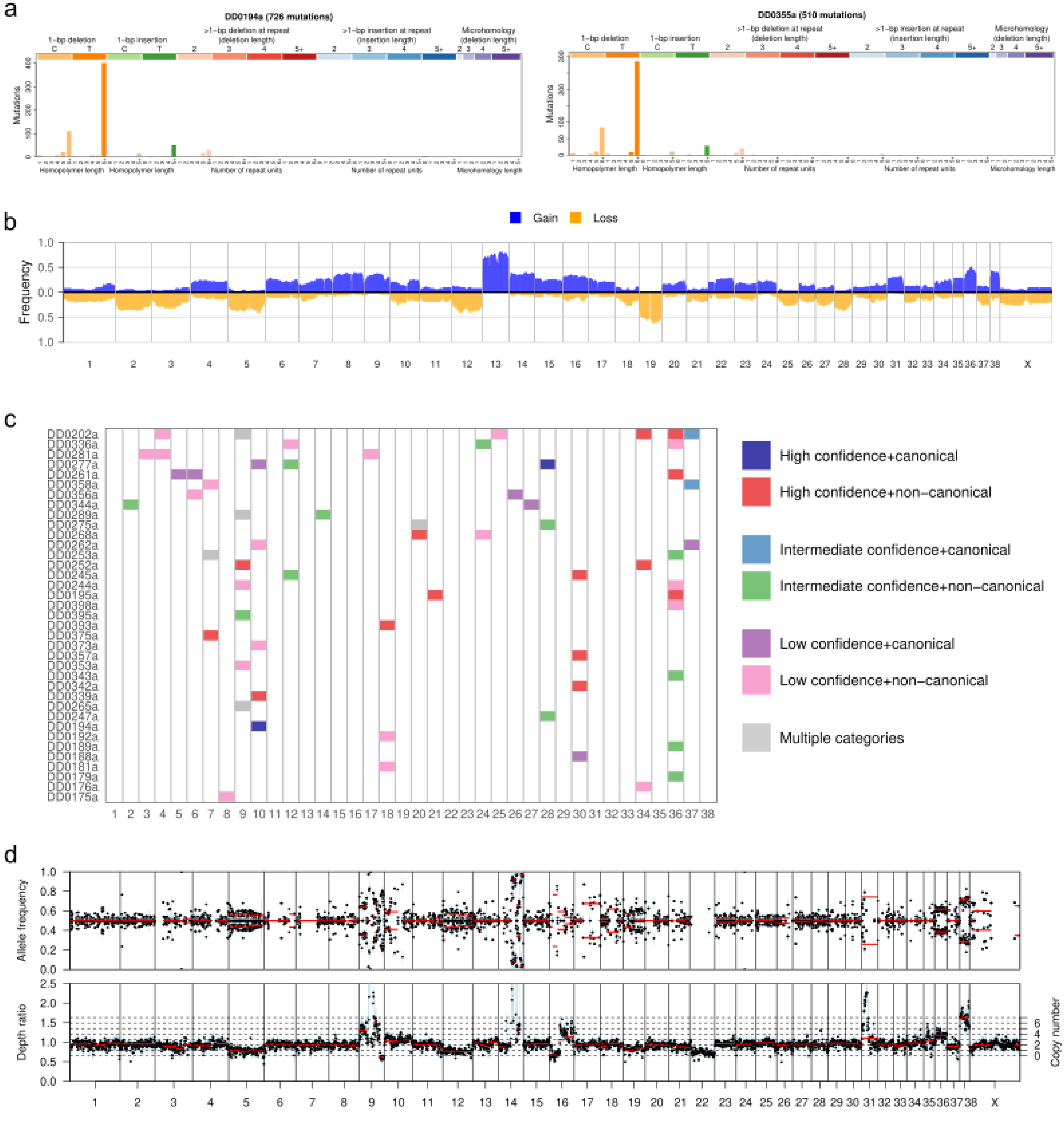

Fig. S4. Indel mutational spectra, copy number and chromothripsis in canine urinary bladder UC. a, Indel mutational spectra for samples DD0194a (left) and DD0355a (right), which show similarities to COSMIC signatures ID2 and ID7. **b,** Somatic copy number alteration (SCNA) penetrance plot (using SCNA data from *n*=62 samples) showing gains and losses 5Mb or larger in 1 Mb windows along each chromosome. **c,** Chromosomes with one or more chromothripsis-like events of high, intermediate and low confidence identified in 37 samples. **d,** Genome plot for DD0289a showing B-allele frequencies and depth ratios derived from sequencing data, with chromothripsis-like events on chromosomes 9 and 14.
