# Supplementary material for: Cross-species oncogenomics offers insight into human muscle-invasive bladder cancer
Source: Genome Biol. 2023 Aug 28;24:191. doi: 10.1186/s13059-023-03026-4 (PMC10464500; doi:10.1186/s13059-023-03026-4)
Supplement: Supplementary file 11 — Additional file 11: Fig. S5. Comparison of known mutational signatures with signatures found in bovine urinary bladder UC and in vitro in treated cell lines. [file 13059_2023_3026_MOESM11_ESM.pdf]

a

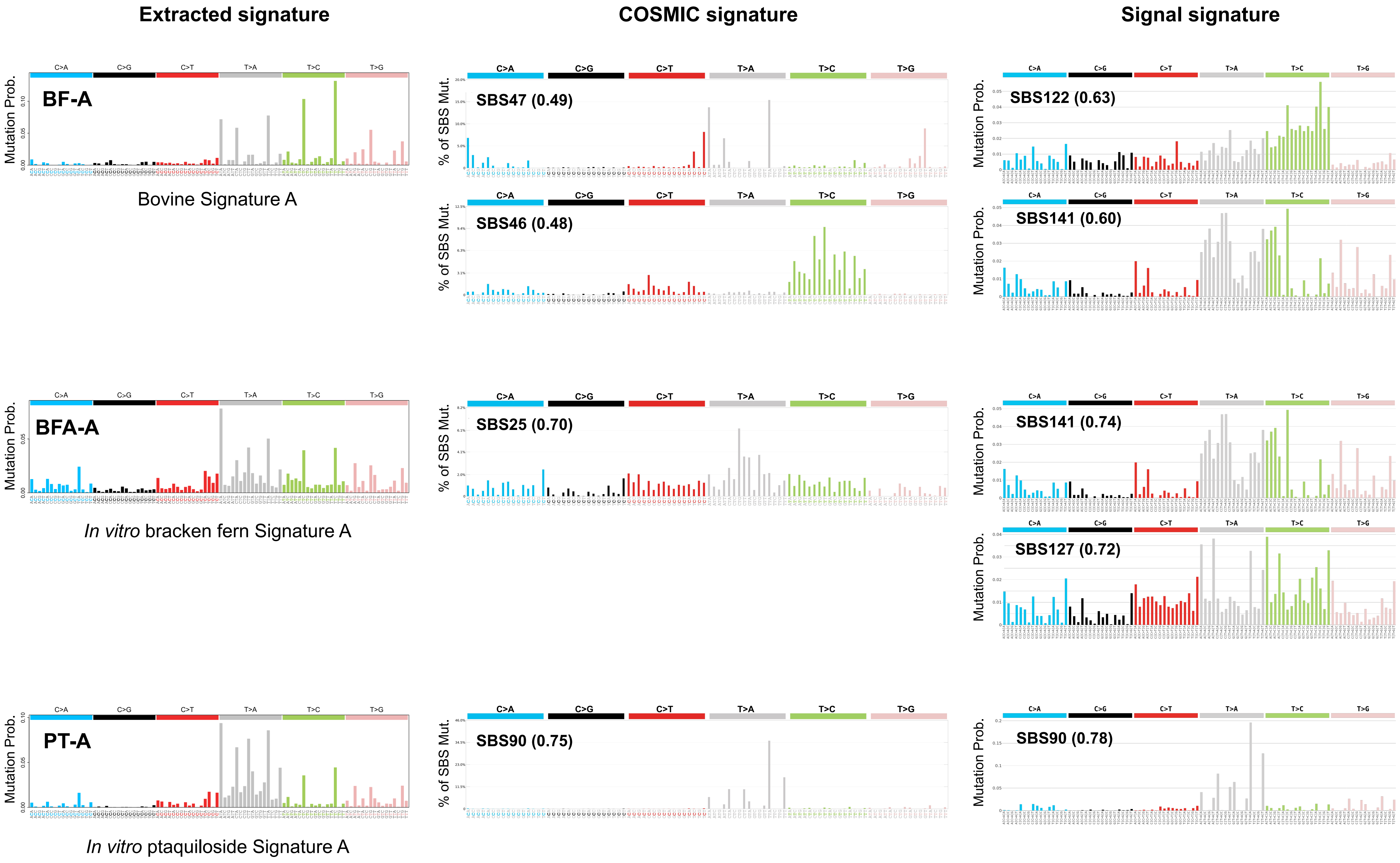
Extracted signature
COSMIC signature
Signal signature
C>A
C>G
C>T
T>A
T>C
T>G
Mutation Prob.
% of SBS Mut.
BF-A
Bovine Signature A
SBS47 (0.49)
SBS46 (0.48)
SBS122 (0.63)
SBS141 (0.60)
BFA-A
In vitro bracken fern Signature A
SBS25 (0.70)
SBS141 (0.74)
SBS127 (0.72)
PT-A
In vitro ptaquiloside Signature A
SBS90 (0.75)
SBS90 (0.78)

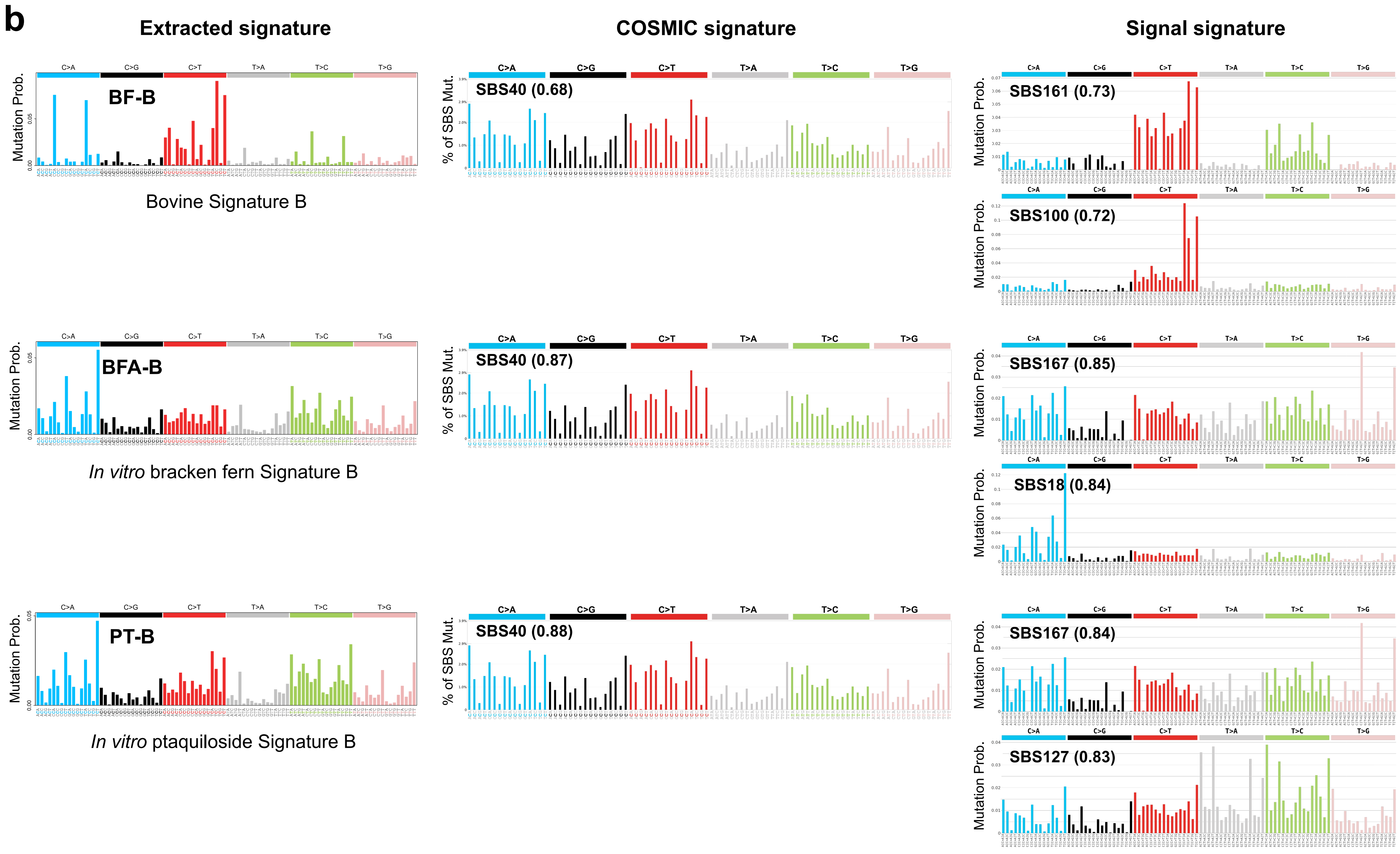

Fig. S5. Comparison of known mutational signatures with signatures found in bovine urinary bladder UC and *in vitro* in treated cell lines. Shown are ***a,*** mutational signature A and ***b,*** signature B in bovine UC (top), KU-19-19 cells treated with bracken fern whole extract (middle) and KU-19-19 cells treated with purified ptaquiloside (lower) and signatures from COSMIC (middle column) and Signal (right column) with the highest cosine similarities (shown in parentheses). The signature of the second highest match is shown if the cosine similarity is within 0.03 of the highest match. The *y*-axis show the mutation probability or percent of total SBS mutations and the *x*-axis shows the mutation context.
