# Supplementary material for: Cross-species oncogenomics offers insight into human muscle-invasive bladder cancer
Source: Genome Biol. 2023 Aug 28;24:191. doi: 10.1186/s13059-023-03026-4 (PMC10464500; doi:10.1186/s13059-023-03026-4)
Supplement: Supplementary file 12 — Additional file 12: Fig. S6. Single base substitution (SBS) mutation spectra showing the number of mutations on the genic transcribed and untranscribed strand. [file 13059_2023_3026_MOESM12_ESM.pdf]

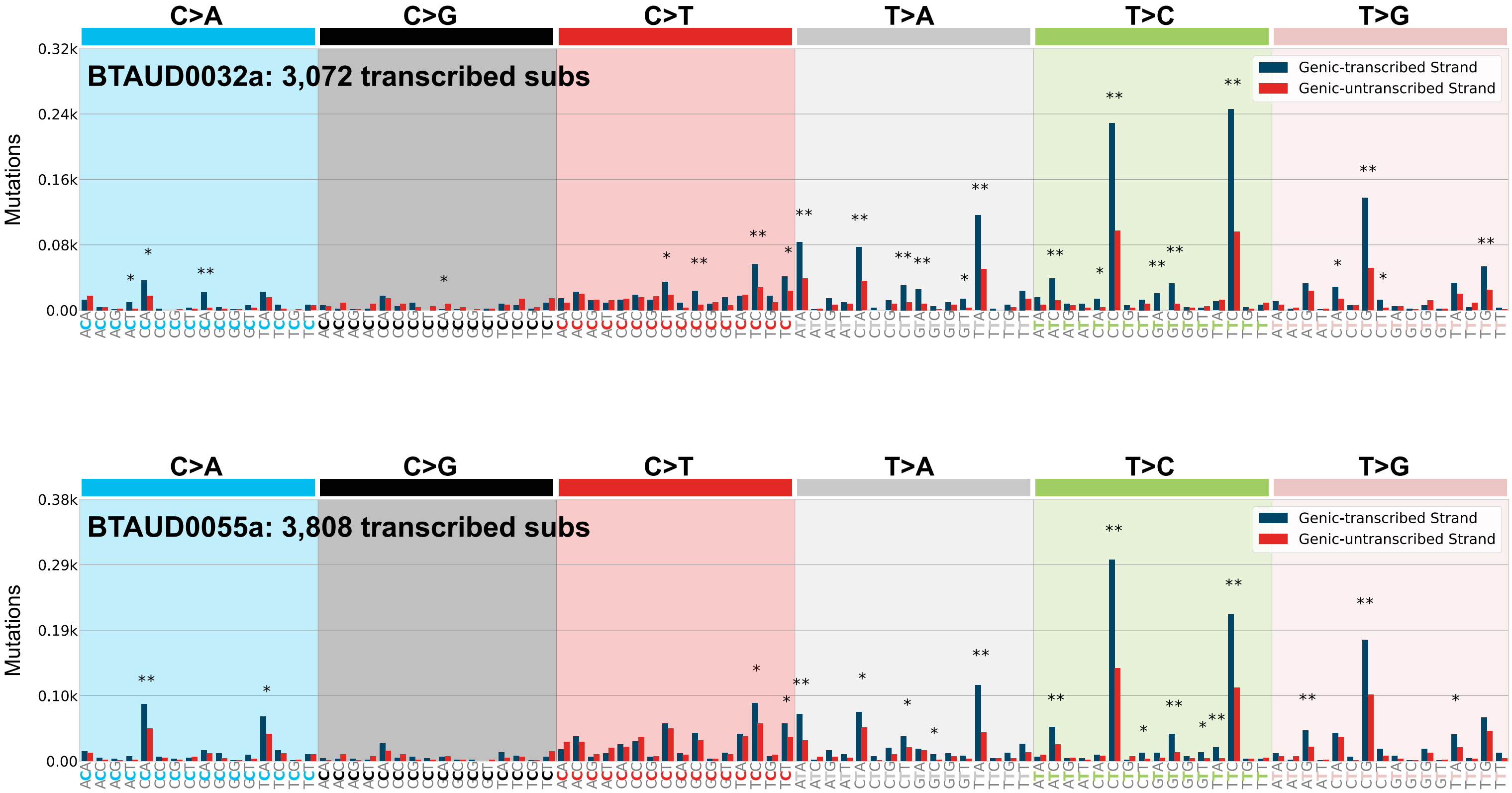

Fig. S6. Single base substitution (SBS) mutation spectra showing the number of mutations on the genic transcribed and untranscribed strand. The spectra from two examples from the bovine UC cohort are shown. The SBS mutation spectra are comprised of 96 substitution types, which are derived from six possible SBS mutations, each with 4 possible bases directly 5' and 3'. Double and single asterisks indicate significant enrichment of mutations on one strand at $p < 0.01$ and $p < 0.05$, respectively.
