# Supplementary material for: Cross-species oncogenomics offers insight into human muscle-invasive bladder cancer
Source: Genome Biol. 2023 Aug 28;24:191. doi: 10.1186/s13059-023-03026-4 (PMC10464500; doi:10.1186/s13059-023-03026-4)
Supplement: Supplementary file 13 — Additional file 13: Fig. S7. Sequence context of single base pair deletions identified in bovine urinary bladder UC. [file 13059_2023_3026_MOESM13_ESM.pdf]

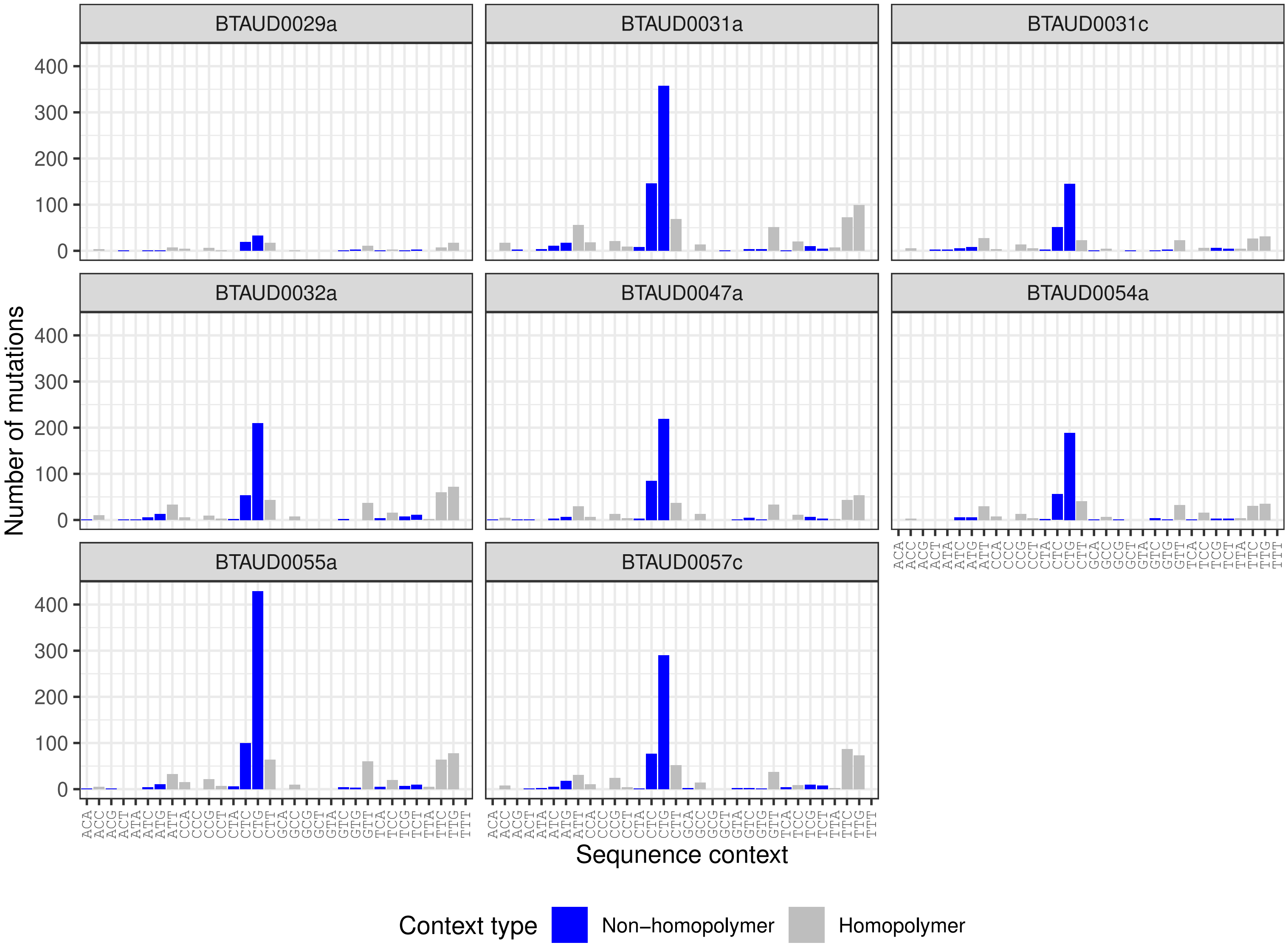

Fig. S7. Sequence context of single base pair deletions identified in bovine urinary bladder UC. Single base pair deletions in each sample are represented in trinucleotide sequence context, where the middle base is the base deleted. Grey bars represent deletions in homopolymers, defined as 2 or more adjacent bases of the same type. Blue bars represent deletions outside of homopolymers.
