# Supplementary material for: Cross-species oncogenomics offers insight into human muscle-invasive bladder cancer
Source: Genome Biol. 2023 Aug 28;24:191. doi: 10.1186/s13059-023-03026-4 (PMC10464500; doi:10.1186/s13059-023-03026-4)
Supplement: Supplementary file 14 — Additional file 14: Fig. S8. Bracken fern. [file 13059_2023_3026_MOESM14_ESM.pdf]

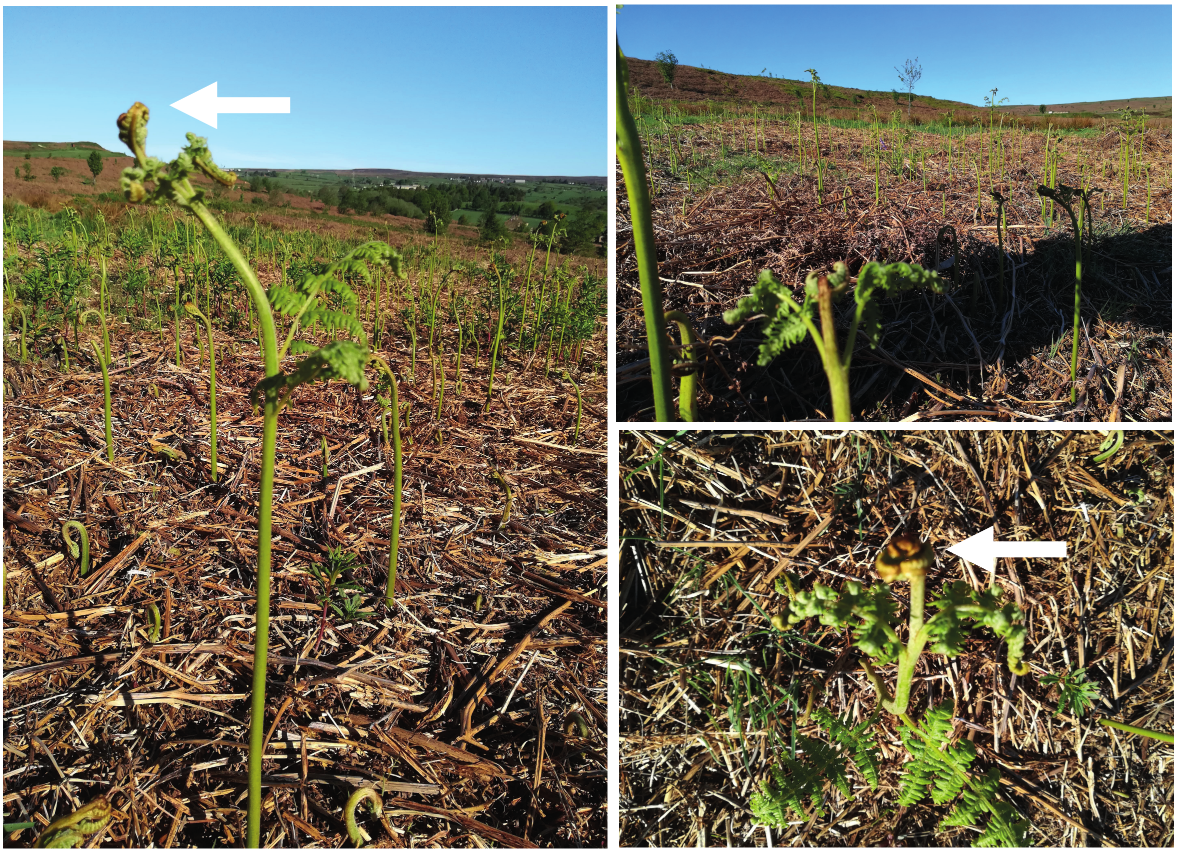

Fig. S8. Bracken fern. Images of bracken fern from the collection site in Baildon Moor, Bradford, West Yorkshire, UK. White arrows indicate the part of the ferns ('fiddleheads') that were picked for generating bracken fern extracts and purified ptaquiloside.
