# Supplementary material for: Cross-species oncogenomics offers insight into human muscle-invasive bladder cancer
Source: Genome Biol. 2023 Aug 28;24:191. doi: 10.1186/s13059-023-03026-4 (PMC10464500; doi:10.1186/s13059-023-03026-4)
Supplement: Supplementary file 15 — Additional file 15: Table S7. Mutational load and signature exposure in cells treated with bracken fern extract from acetone extraction. [file 13059_2023_3026_MOESM15_ESM.pdf]

Table S7. Mutational load and signature exposure in cells treated with bracken fern extract from acetone extraction.

Exposure dose (μg/mL)	Length of exposure (days)	Total number of mutations	Signature BFA-A exposure (% of total mutations)	Signature BFA-B exposure (% of total mutations)
**IC_{20}**				
49.1	3	1179	48.3	51.7
11.1	7	855	7.4	92.6
3.1	10	1064	12.2	87.8
6.3	14	822	8.8	91.2
**IC_{50}**				
>79.7	3	1316	67.7	32.3
28.2	7	912	36.2	63.8
12.0	10	1165	18.5	81.5
18.5	14	1289	15.8	84.2

The proportion of activity (exposure) of each signature, BFA-A and BFA-B, is shown, along with the total number of mutations. Mutation counts have been corrected to account for the number of sites considered. Varying doses were used for each time point (see Methods). Signature BFA-A has similarity to bovine Signature BF-A.
