# Supplementary material for: Cross-species oncogenomics offers insight into human muscle-invasive bladder cancer
Source: Genome Biol. 2023 Aug 28;24:191. doi: 10.1186/s13059-023-03026-4 (PMC10464500; doi:10.1186/s13059-023-03026-4)
Supplement: Supplementary file 16 — Additional file 16: Table S8. Mutational load and signature exposure in cells treated with bracken fern extract from ethyl acetate extraction. [file 13059_2023_3026_MOESM16_ESM.pdf]

Table S8. Mutational load and signature exposure in cells treated with bracken fern extract from ethyl acetate extraction

Exposure dose (μg/mL)	Length of exposure (days)	Total number of mutations	Signature BFE-A exposure (% of total mutations)	Signature BFE-B exposure (% of total mutations)
**IC_{20}**				
49.1	3	1304	58.0	42.0
14.6	7	880	21.0	79.0
4.6	10	824	9.8	90.2
5.6	14	1151	11.4	88.6
**IC_{50}**				
34.1	7	1389	55.9	44.1
12.9	10	1039	22.5	77.5
13.1	14	1205	23.6	76.4

The proportion of activity (exposure) of each signature, BFE-A and BFE-B, is shown, along with the total number of mutations. Mutation counts have been corrected to account for the number of sites considered. Varying doses were used for each time point (see Methods). Signature BFE-A has similarity to bovine Signature BF-A.
