# Supplementary material for: Cross-species oncogenomics offers insight into human muscle-invasive bladder cancer
Source: Genome Biol. 2023 Aug 28;24:191. doi: 10.1186/s13059-023-03026-4 (PMC10464500; doi:10.1186/s13059-023-03026-4)
Supplement: Supplementary file 17 — Additional file 17: Fig. S9. Mutational signatures in human bladder cancer cell lines after exposure to bracken fern extracts. [file 13059_2023_3026_MOESM17_ESM.pdf]

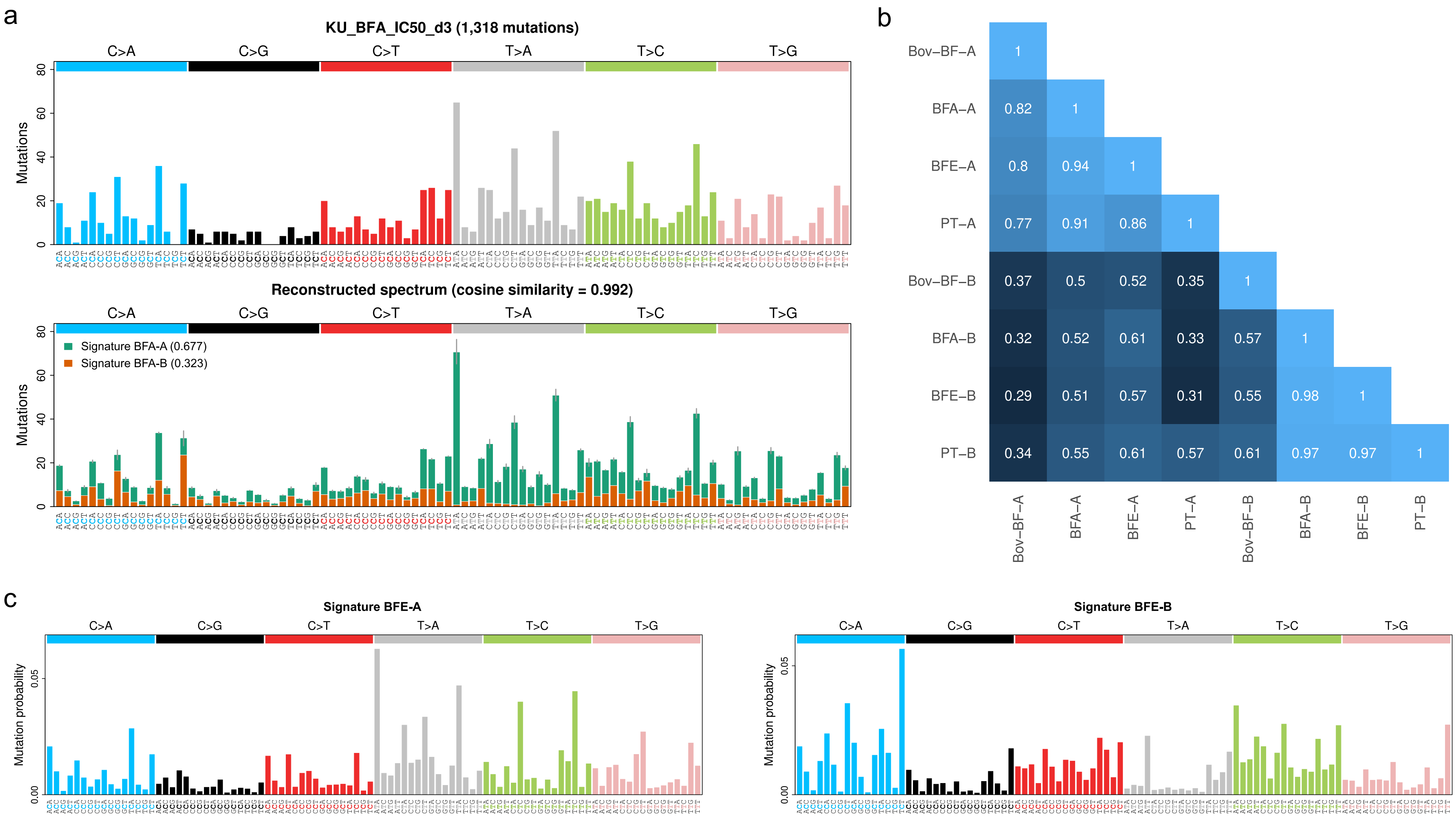

Fig. S9. Mutational signatures in human bladder cancer cell lines after exposure to bracken fern extracts. a, The observed (upper panel) and reconstructed (lower panel) single base substitution (SBS) spectra for human KU-19-19 cells treated for 3 days (IC_{50}) with bracken fern whole extract from acetate extraction (BFA). The reconstructed spectrum shows the activity, or exposure, of each signature for each substitution type. **b**, The cosine similarities between SBS signatures identified in bovine UC (Bov-BF) and KU-19-19 cells treated with either BFA, bracken fern whole extract from ethyl acetate extraction (BFE) or ptaquiloside (PT). **c,** Novel SBS signatures, designated Signature BFE-A and BFE-B, identified in KU-19-19 cells treated with BFE. The SBS mutation spectra are comprised of 96 substitution types, which are derived from six possible SBS mutations, each with 4 possible bases directly 5’ and 3’.
