# Supplementary material for: Cross-species oncogenomics offers insight into human muscle-invasive bladder cancer
Source: Genome Biol. 2023 Aug 28;24:191. doi: 10.1186/s13059-023-03026-4 (PMC10464500; doi:10.1186/s13059-023-03026-4)
Supplement: Supplementary file 18 — Additional file 18: Fig. S10. Single base substitution (SBS) mutation spectra and transcriptional strand bias in ptaquiloside-treated human bladder UC cells. [file 13059_2023_3026_MOESM18_ESM.pdf]

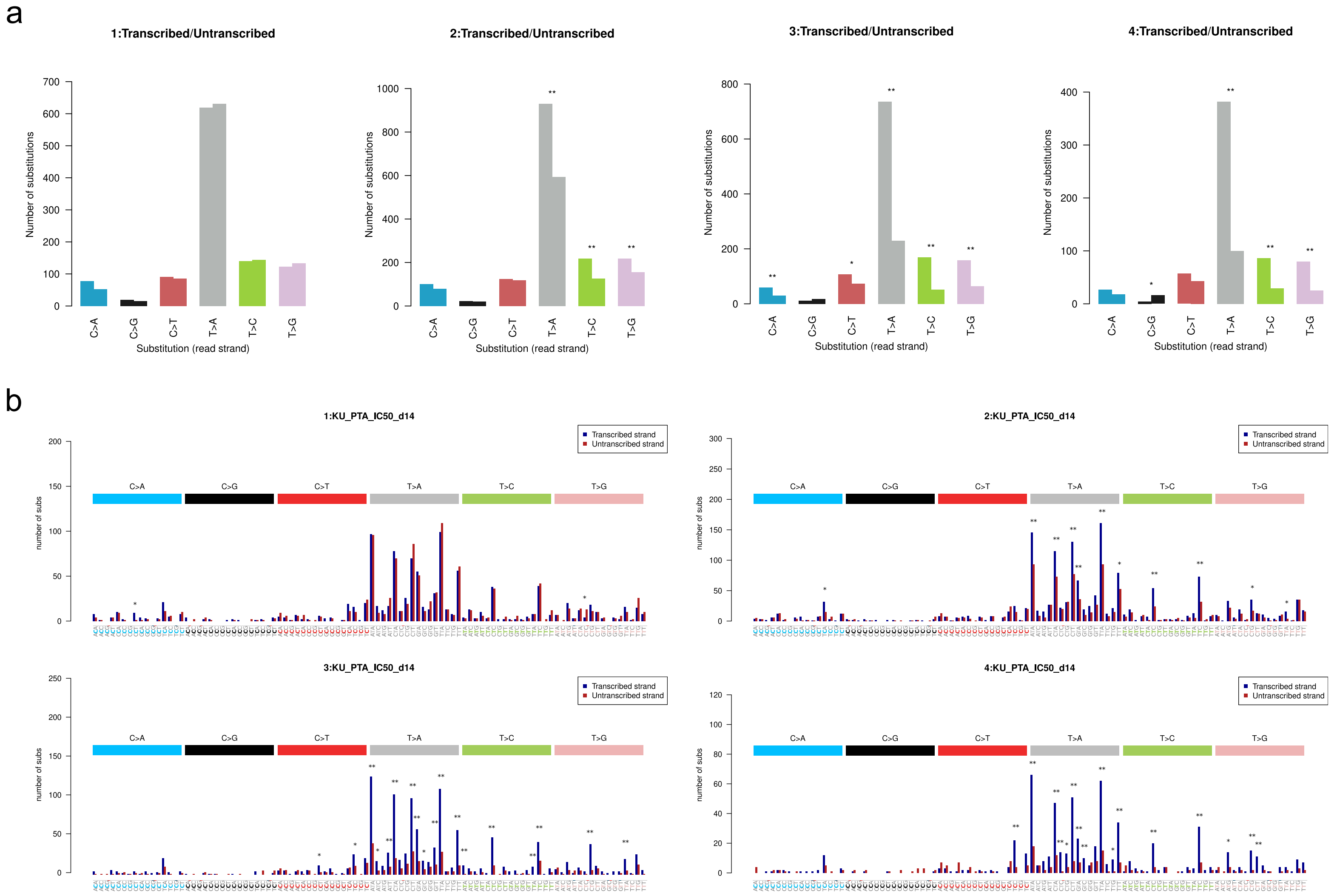

Fig. S10. Single base substitution (SBS) mutation spectra and transcriptional strand bias in ptaquiloside-treated human bladder UC cells. a, Counts of mutations on the genic transcribed and untranscribed strands in genes with low (group 1) to high (group 4) levels of expression. **b,** Counts of mutations in specific trinucleotide context on the genic transcribed and untranscribed strands in genes with low (group 1) to high (group 4) levels of expression. The SBS mutation spectra are comprised of 96 substitution types, which are derived from six possible SBS mutations, each with 4 possible bases directly 5′ and 3′. Double and single asterisks indicate significant enrichment of mutations on one strand at $p < 0.01$ and $p < 0.05$, respectively.
