# Supplementary material for: Cross-species oncogenomics offers insight into human muscle-invasive bladder cancer
Source: Genome Biol. 2023 Aug 28;24:191. doi: 10.1186/s13059-023-03026-4 (PMC10464500; doi:10.1186/s13059-023-03026-4)
Supplement: Supplementary file 19 — Additional file 19: Fig. S11. The indel mutation spectra from ptaquiloside-treated human bladder UC cells and bovine urinary bladder UC. [file 13059_2023_3026_MOESM19_ESM.pdf]

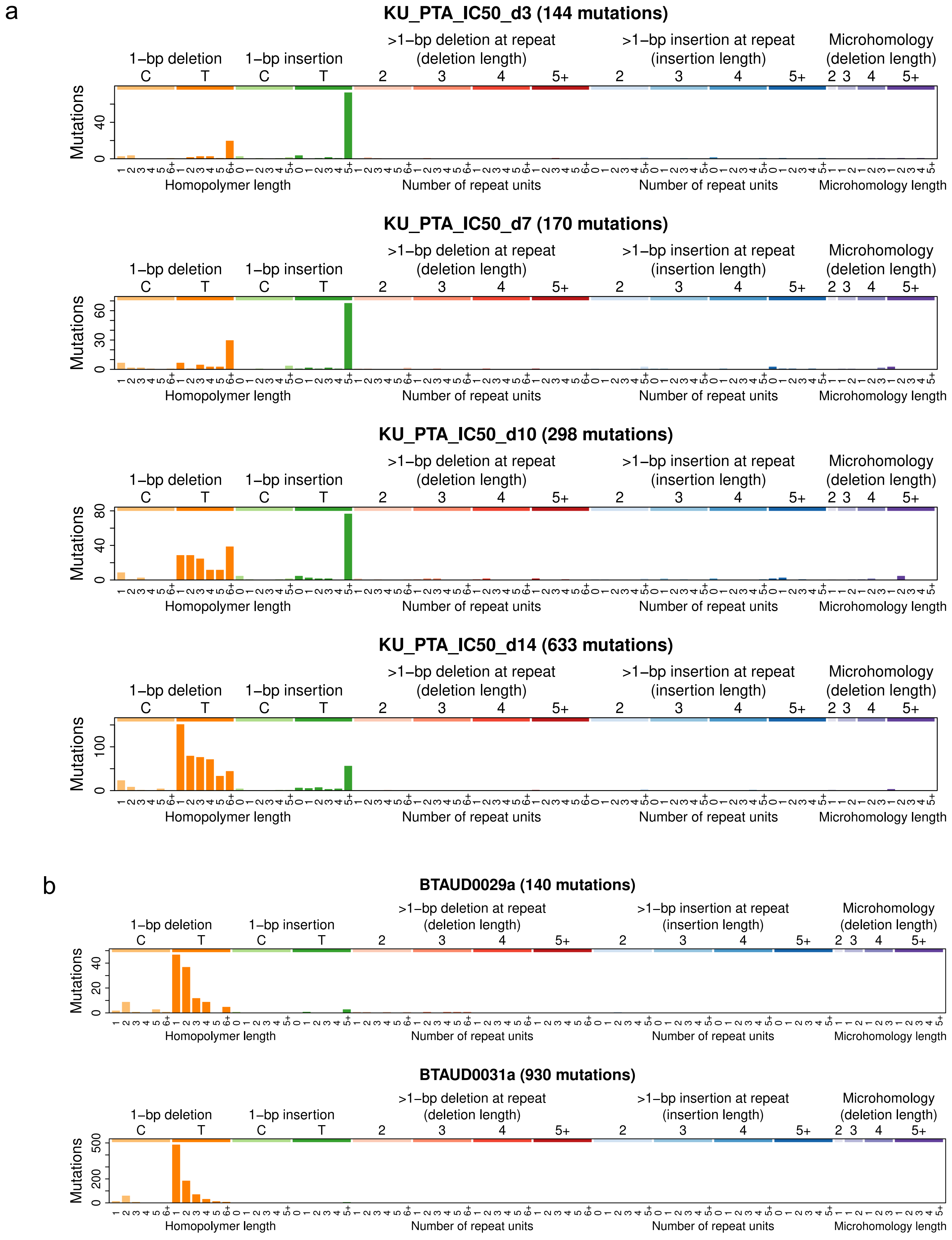

Fig. S11. The indel mutation spectra from ptaquiloside-treated human bladder UC cells and bovine urinary bladder UC. a, Indel mutation spectra of ptaquiloside-treated KU-19-19 cells (IC_{50}), at different time points; d3, d7, d10 and d14 are the number of days. **b,** The indel mutation spectra of bovine UC cases with the lowest (upper panel) and highest (lower panel) number of indels.
