# Supplementary material for: Cross-species oncogenomics offers insight into human muscle-invasive bladder cancer
Source: Genome Biol. 2023 Aug 28;24:191. doi: 10.1186/s13059-023-03026-4 (PMC10464500; doi:10.1186/s13059-023-03026-4)
Supplement: Supplementary file 24 — Additional file 24: Fig. S13. Somatic copy number alterations in bovine urinary bladder UC. [file 13059_2023_3026_MOESM24_ESM.pdf]

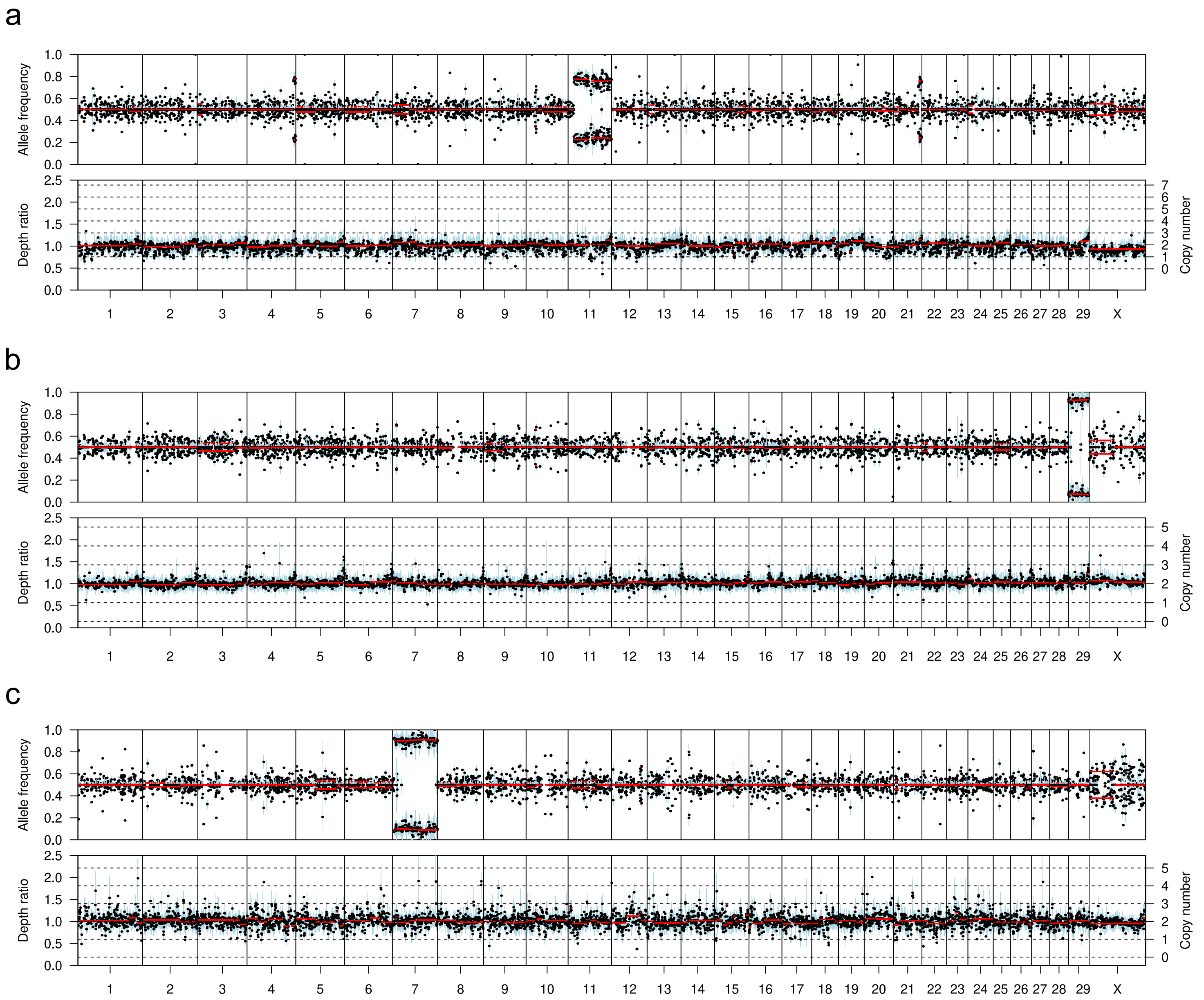

Fig. S13. Somatic copy number alterations in bovine urinary bladder UC. Genome plots showing B-allele frequencies and depth ratios derived from sequencing data for samples **a,** BTAUD0029a, **b,** BTAUD0031c and **c,** BTAUD0055a.
