# Supplementary material for: Cross-species oncogenomics offers insight into human muscle-invasive bladder cancer
Source: Genome Biol. 2023 Aug 28;24:191. doi: 10.1186/s13059-023-03026-4 (PMC10464500; doi:10.1186/s13059-023-03026-4)
Supplement: Supplementary file 25 — Additional file 25: Fig. S14. Chromothripsis-like events in feline urinary bladder UC. [file 13059_2023_3026_MOESM25_ESM.pdf]

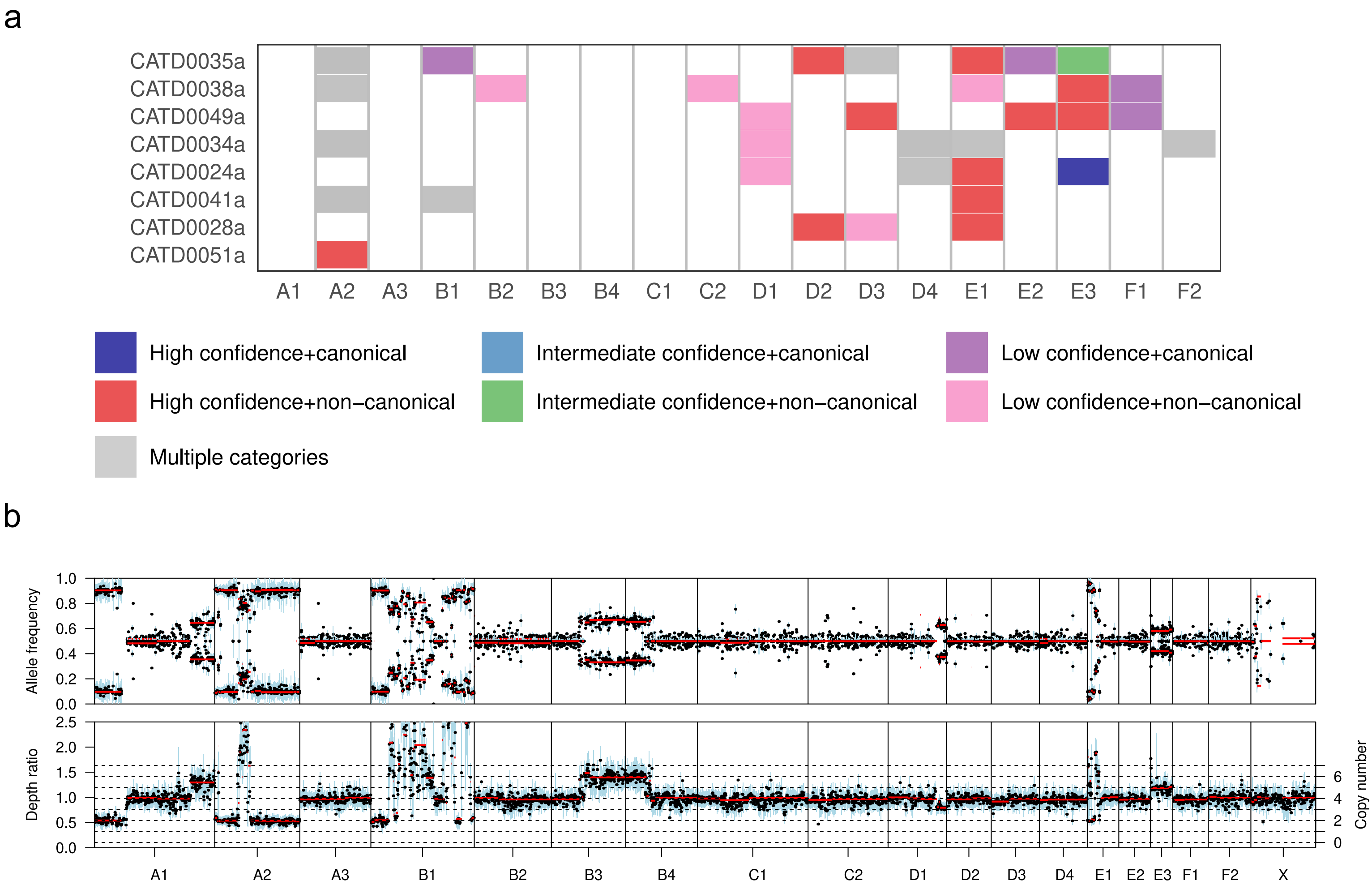

Fig. S14. Chromothripsis-like events in feline urinary bladder UC. a, Chromosomes with one or more chromothripsis-like events of high, intermediate and low confidence identified in 8 samples. **b,** Genome plot for CATD0041a, showing B-allele frequencies and depth ratios derived from sequencing data, with chromothripsis-like events on chromosomes A2, B1 and E1.
