# Supplementary material for: Cross-species oncogenomics offers insight into human muscle-invasive bladder cancer
Source: Genome Biol. 2023 Aug 28;24:191. doi: 10.1186/s13059-023-03026-4 (PMC10464500; doi:10.1186/s13059-023-03026-4)
Supplement: Supplementary file 20 — Additional file 20: Fig. S12. Sequence context single base pair deletions identified in ptaquiloside-treated human bladder UC cells. [file 13059_2023_3026_MOESM20_ESM.pdf]

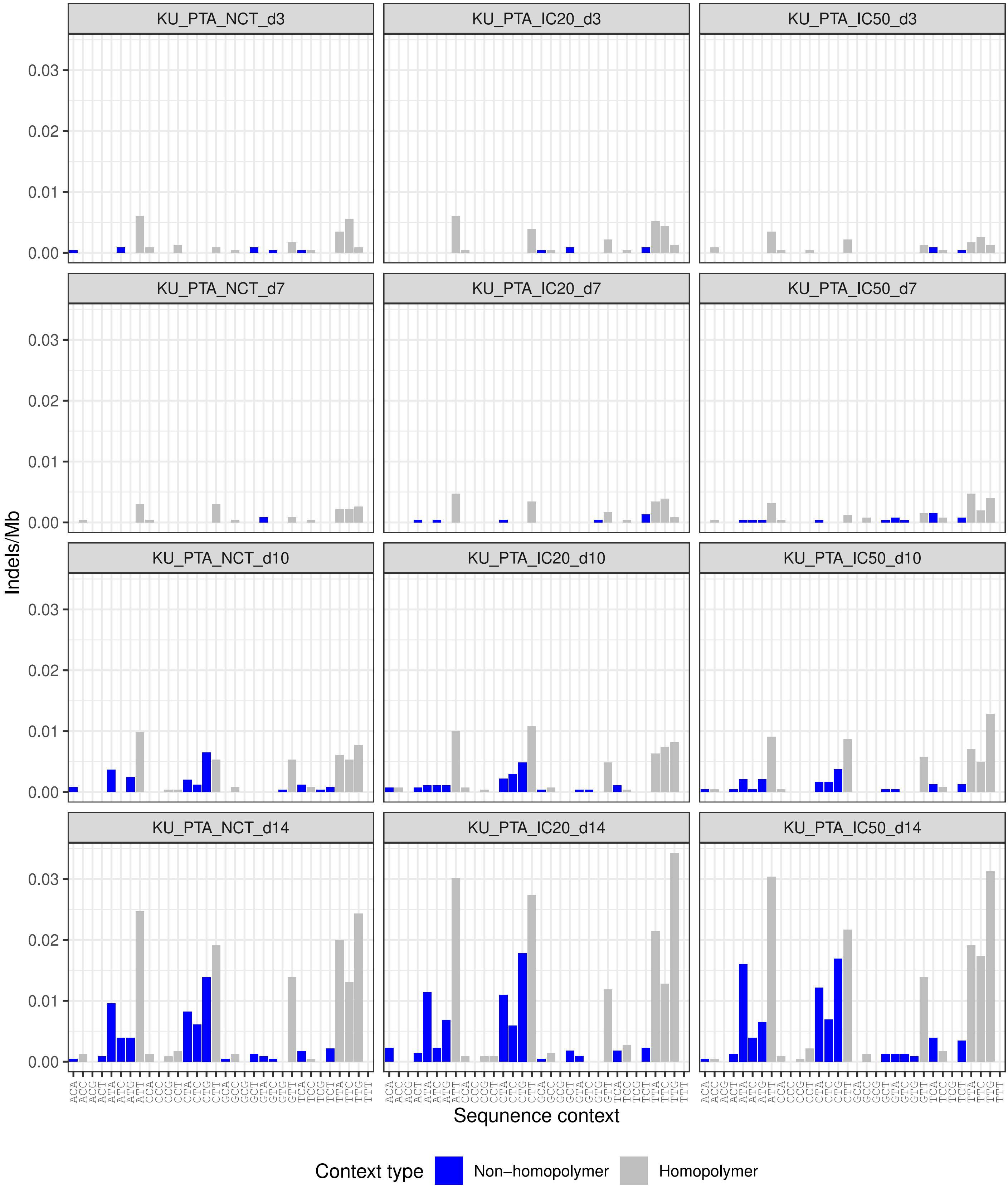

Fig. S12. Sequence context of single base pair deletions identified in ptaquiloside-treated human bladder UC cells. Single base pair deletions are represented in trinucleotide sequence context, where the middle base is the base deleted. Grey bars represent deletions in homopolymers, defined as 2 or more adjacent bases of the same type. Blue bars represent deletions not in homopolymers. NTC is non-toxic concentration; IC_{20} and IC_{50} are 20% and 50% inhibitory concentration of cell growth, respectively; d3, d7, d10 and d14 are the number of days.
