# Supplementary material for: Cross-species oncogenomics offers insight into human muscle-invasive bladder cancer
Source: Genome Biol. 2023 Aug 28;24:191. doi: 10.1186/s13059-023-03026-4 (PMC10464500; doi:10.1186/s13059-023-03026-4)
Supplement: Supplementary file 27 — Additional file 27: Table S13. Commonly amplified or deleted cancer genes from a comparison of focal amplifications and deletions across human, canine and feline urinary bladder UC. [file 13059_2023_3026_MOESM27_ESM.pdf]

Table S13. Commonly amplified or deleted cancer genes from a comparison of focal amplifications and deletions across human, canine and feline urinary bladder invasive UC.

Regions compared	Human vs dog		Human vs cat		Dog vs cat	
	Amplified	Deleted	Amplified	Deleted	Amplified	Deleted
5Mb (cat, dog), focal targets (human)	*MDM2*	-	*PPARG*	*CREBBP*	-	-
10Mb (cat, dog), focal targets (human)	*MDM2*	*RB1*	*PPARG*	*CREBBP*, *NCOR1*	-	-
20Mb (cat, dog), focal targets (human)	*MDM2*	*RB1*	*PPARG*	*CREBBP*, *NCOR1*	-	-
5Mb (cat, dog), wide peak (human)	*MDM2, ZFHX3*	*FSTL3, STK11, TCF3*	*PPARG, RNF213*	*CREBBP*	-	-
10Mb (cat, dog), wide peak (human)	*MDM2, ZFHX3*	*FSTL3, STK11, TCF3*, *RB1*	*PPARG, RNF213*	*CREBBP, NCOR1, ARHGEF10*	-	-
20Mb (cat, dog), wide peak (human)	*MDM2, ZFHX3*	*FSTL3, STK11, TCF3*, *RB1*, *CASP3, FSTL3, ARHGEF10*	*PPARG, RNF213*	*CREBBP, NCOR1, ARHGEF10*	-	*ARHGEF10*

Significantly amplified or deleted focal regions in feline, canine and human UC were compared to identify commonly amplified or deleted Cancer Gene Census (CGC) or bladder cancer (BLCA) genes. For each pairwise comparison, syntenic regions and common genes within focal regions were identified. For feline and canine UC, focal regions with a maximum size of 5Mb, 10Mb and 20Mb (see Methods) were compared to focal target regions identified in human MIBC and wider peak regions from GISTIC analysis.
