# Supplementary material for: Cross-species oncogenomics offers insight into human muscle-invasive bladder cancer
Source: Genome Biol. 2023 Aug 28;24:191. doi: 10.1186/s13059-023-03026-4 (PMC10464500; doi:10.1186/s13059-023-03026-4)
Supplement: Supplementary file 26 — Additional file 26: Table S12. Syntenic regions in human, canine and feline UC on recurrently amplified or deleted chromosomes. [file 13059_2023_3026_MOESM26_ESM.pdf]

Table S12. Syntenic regions in human, canine and feline UC on recurrently amplified or deleted chromosomes.

Alteration	Human	Dog	Cat	Common CGC/BLCA genes
Deletion	5:75,007,193-112,887,482 (synteny with dog only)	3:228,325-31,235,237	A1:141,840,445-174,631,577	*APC, ARRDC3, ARSK, ELL2, RFESD, ARSB, STARD4*
Amplification	7:31,800-6,733,390	6:11,270,653-16,620,345	E3:286,913-16,425,008	*CARD11, GPER1, PMS2, PRKAR1B, RAC1, RNF216, ZNF12*
Deletion	10:50,157,926-58,166,360	28:75,216-1,927,439	D2:48,011,741-90,168,174 (synteny with human only)	*PARG, NCOA4, ARHGAP22*

Syntenic regions within recurrently altered chromosomes (dog and cat) or chromosome arms (human) were identified for each pairwise comparison (human-dog, human-cat, dog-cat). Shown are Cancer Gene Census (CGC) or bladder cancer (BLCA) genes in syntenic regions commonly amplified or deleted in all 3 species.
