# Supplementary material for: Cross-species oncogenomics offers insight into human muscle-invasive bladder cancer
Source: Genome Biol. 2023 Aug 28;24:191. doi: 10.1186/s13059-023-03026-4 (PMC10464500; doi:10.1186/s13059-023-03026-4)
Supplement: Supplementary file 28 — Additional file 28. Review history. [file 13059_2023_3026_MOESM28_ESM.docx]

Review History

**First round of review**

**Reviewer 1**

**Are you able to assess all statistics in the manuscript, including the appropriateness of statistical tests used?** Yes, and I have assessed the statistics in my report.

**Comments to author:**

Wong et al. have presented detailed comparative genomics analysis of muscle-invasive bladder cancer (MIBC) across humans, canine, feline, and bovine species. They sequenced the largest non-human MIBC exome data (n=118) for tumor-normal pairs and did comprehensive somatic mutation analysis (mutational burden, mutational signatures, and driver gene discovery) by comparing data to previously published human MIBC cohort (n=412). Key findings from this study were: 1. Cross-species convergence of somatic driver mutations and focal syntenic copy-number changes in an otherwise dissimilar somatic mutational load (coding mutations per Mb of genome) observed in this comparative analysis, and 2. Identification of an environmental exposure (bracker fern (BF)) induced unique mutational processes in all eight of geographically separated bovine cases with in vitro cell line experiments suggesting potential carcinogenic activity in humans exposed to either BF extracts or its purified metabolite, ptaquiloside. This study involved significant collaborations across 17 countries to acquire tissue specimens and authors have performed extensive comparative analysis while ensuring rigorous implementation of methods towards somatic variant calling, significance test for somatic variants, including copy-number segments. This study is of much relevance to highlight value of comparative genomics of studying spontaneous cancers, especially to characterize conserved cancer drivers and underlying mutational processes that are observed across species. I have a few concerns related to mutational burden and mutational signature analysis.

1. For mutational burden analysis, are mutations comprise of single-nucleotide variants (SNVs) or also include SNV with indels? If the latter, is there a significant difference in bovine vs. canine + feline cohort when counting numbers per Mb for SNV vs. indels? I believe exome capture kit was custom tailored for each of canine, feline, and bovine genes, and somatic variants were filtered for FFPE artifacts. If so, do those artifacts show relative bias in bovine vs. other species? If not, much higher observed coding mutational burden in bovine UC cases is biologically intriguing even after factoring for BF extract induced mutatgenesis.

2. For recurrent hotspot driver mutations, do these driver events vary or enriched within a subset of cohort, e.g., driver events could vary across low vs. high mutational burden samples, and if so additional comparative analysis by factoring in different mutational burden could help identify driver events in say MMR-deficient vs. MMR-intact cohort?

3. Minor: Line 239-240: Unclear why authors state "appeared to be" biallelic inactivation of MSH2. Could this be supported with PCR/RNA-Seq if tissue specimen is still available? If not, statement provides less clarity and more confusion to readers.

4. Since mutational signatures, especially de novo ones in bovine cases were based on exome and not whole-genome data, authors should state such limitation of inferring genome-wide signatures based on exome data alone and could have provided additional support using whole-genoem dataset, e.g., possible if a published whole-genome dataset exists for bovine cases (normal or tumors).

5. Given the study involves detailed comparative analysis, for each of major sections showing key findings, readers may find it easier to navigate the paper if each sectiion starts with rationale and ends with a key one to two findings and highlighting biological/clinical relevance and move numbers to supplementary methods.

**Reviewer 2**

**Are you able to assess all statistics in the manuscript, including the appropriateness of statistical tests used?** Yes, and I have assessed the statistics in my report.

**Comments to author:**

This article by Wong and colleagues presents an interesting data set of whole exomes from canine, feline and bovine urothelial carcinomas (UC). The authors compare driver mutation profiles, mutational signatures and somatic copy number alterations with public human UC data and describe noteworthy similarities and differences among species. They also investigate whether an unknown mutational signature found in bovine tumors can be explained by bracken fern mutagenesis through a series of in vitro experiments.

Overall I found this paper very pleasant and straightforward to read. I have no significant concerns from a technical point of view, the sequencing data analysis is evidently well done, it recovers some known features (e.g. of canine tumors) and points to some new observations. From a narrative point of view, I think the authors could improve the impact of the manuscript if they challenged themselves to explain in more detail how cross-species analysis could contribute to our understanding of human tumors. If we observe similarities/differences in driver gene and/or SCNA frequency among species, what exactly does that teach us about the biology of UC? Do the authors have any hypotheses to explain the notable differences (BRAF mutation frequency in canines, lack of TP53 etc.)? Did the results of their analysis yield what the authors were hoping for at the outset of the work - why or why not? What could be the reason for the lack of SCNAs in bovine tumors? Currently the text is very technical, essentially a long list of similarities and differences. Giving it more biological/interpretive depth would benefit the article.

I do have one scientific concern with respect to the section in which the authors perform in vitro experiments to ascertain whether the mutational signatures recovered from bovine tumors are caused by bracken fern. First of all, this section is very hard to follow, as all the signatures have been given relatively similar names. But more importantly, it is not clear to me whether the BF in vitro experiment really recovered the bovine signature as the authors claim it did. Throughout the text, the authors state that if newly extracted signatures have a cosine similarity of less than 0.9 to existing signatures, they are considered unrelated. However, when comparing e.g. BFA-A and bovine signature BF-A, the authors conclude "strong resemblance" (line 306) based on a similarity of 0.82. By their own measuring stick this similarity should be considered weak. The same problem arises for comparison of the PT signatures and the bovine signatures. Again, the text is confusing due to an abundance of similar acronyms but it did not really clarify for me whether the authors had indeed proven that bracken fern was the origin of the bovine signatures.

**Reviewer 3**

**Are you able to assess all statistics in the manuscript, including the appropriateness of statistical tests used?** Yes, and I have assessed the statistics in my report.

**Comments to author:**

The authors perform a cross species comparison of bladder cancer using WES of canine (n=87), feline (n=23) and bovine (n=8) matched tumor-normal pairs.

They use human WES data was from TCGA study (Robertson et al. Cell, 2017) from 412 cases. They also highlight the role of a specific carcinogen (ptaquiloside).

The study is technically robust and provides interesting new insights.

We suggest to address the following issues:

1. Rationale for this study:

The authors mention that one goal of the study is to identify key driver genes and to refine cancer driver genes in human cancer (e.g. line 149).

Even though we agree that comparison across species is an interesting basic research question, there are other strategies to identify human cancer drivers that may be more relevant. We would suggest to tone down this particular goal, as other approaches to find human cancer driver genes seem more appropriate.

The authors argue that cat and dog models have advantages as compared to mouse models because they share the same environment as humans.

However, the exposure and risk factors are very different between humans and animals.

Even though they share the same environment, humans smoke, drink, have an unhealthy diet, get exposure at their workplace etc, which play a critical role in human cancer (not addressed here).

2. Relevance of the psaquiloside signature in humans

Chronic ingestion of braken fern is not really relevant in humans.

As the psaquiloside signature is not detected in humans, it seems like exposure from the milk or groundwater may not be sufficient.

The relevance of ptaquiloside exposure in humans is questionable. If this would be a carcinogen it would be important to investigate the role of this carcinogen, but from the presented data the evidence is limited.

3. Differences in signatures across countries/regions

It would be interesting to search for differences in signatures between animals and humans from different regions and countries, also between cows that are outside or kept inside.

The signatures might also be interesting outside the context of bladder cancer (e.g.

If the spores play a role, as suggested by the authors, then lung cancer might be interesting).

4. Cancer drivers

Line 470-475: were these drivers not already known before in bladder cancer?

It seems like the vast majority was already known (e.g. KDM6A, TP53, NRAS, MDM2, RB1 reported) https://doi.org/10.1038/s41467-019-08576-5)

Are there any novel drivers that were identified by this study?

If so, emphasizing these and role of these novel drivers might strengthen the study.

One potential new tumor suppressor gene (ARHGEF10) is selected, validations or further analyses on the role of this gene may be interesting.

5. The authors argue that cat and dog tumors can offer an animal model.

Even through the heterogeneity may be an advantage, spontaneous tumors are hard to use to test treatments in terms of logistics, due to the need to wait for the development of these tumors. There may be applications but limited

6. Experimental validation of the signature:

Inducing the mutational signature on normal cells and not on cancer cells would be more interesting.

Phenotypically: what else happens to the cells treated with the carcinogen? Any consequences on the transcriptome?

7. Could the authors comment on the absence of APOBEC signature and on the fact that the mutational load is not as high in animals?

It was revealed previously that the somatic mutation rate differs significantly among species and is inversely correlated with their lifespan [Nature 604, 517–524 (2022)]. However, it remains unclear whether the author considered this factor and employed an adjusted mutation rate for species comparison. Furthermore, there is insufficient evidence in this paper to support the claim that the elevated mutation rate observed in bovine is attributable to a potent environmental mutagen.

8. Methods

For canine and feline data: the authors feed existing signatures

For bovine tumors they do de novo discovery

It would be good to do de novo discovery as well on canine and feline tumors for consistency.

There is also a lack of detail regarding the de-novo estimation of mutational signatures in bovine samples. The authors mention that they used goodness of fit plots to select the number of signatures. Ideally, such plots should be provided in supplement, or at the very least, more details should be provided about how goodness of fit was evaluated.

Please also provide the code for the analyses.

9. Statistics missing for signatures

Goodness of fit plot for signatures are missing

There is not so much information on the exact statistical analysis used.

For example:

Line 618-619: please write down which statistical test was used.

Line 626: please write down which statistical test was used.

If 15 genes are identified as significant in 1 species and 12 genes in another species

How many are in common, and what is the chance of that happening randomly

LRP1B identified in a cow with 2 tumors

The authors state that it is a driver because identified in both

Maybe just hit twice independently, just a passenger

Based on n=2, hard to tell this is a driver

Strong statement

Effect of the mutation?

10. Germline variants

Discovery algorithm only used among somatic mutations

Do the authors identify any germline variants?

Only the prevalence of germline variants is reported

Do specific germline drivers co-occur with somatic drivers? Predispose to?

Mutual exclusivity and co-occurrence are evaluated among somatic mutations. Was there any co-occurrence or exclusivity detected with germline variants?

11. CT scoring and copy-number calling

There is not sufficient detail provided about how the chromothripsis scoring was done. Voronina et al. was referenced for the method of scoring, however, the referenced manuscript did both manual and computer assisted scoring. Additionally, the scoring in Voronina et al. was done after copy number calling following alignment of all reads from the exome sequencing, ignoring the baited regions. Was a similar method employed by the authors?

It would be necessary to provide a more detailed explanation of the methodology used to estimate somatic copy number alteration. Specifically, the reference genome, window size, and normal control samples should be further elucidated. Additionally, it is unclear how the authors identified and excluded samples with excessive noise; it is imperative to outline the selection criteria employed in the analysis.

12. When they look for similarity to existing signatures, the authors use a cosine cut off 0.9

But between bovine and treated cell lines: the cut-off lowered to 0.82

A consistent cut off would be better

13. Figure 6

Comparison of the signatures to COSMIC signatures

It would be nice to see the full comparison, also to understand the cut off of 0.9

**Authors Response**

**Point-by-point responses to the reviewers’ comments:**

**Reviewer #1**:

Wong et al. have presented detailed comparative genomics analysis of muscle-invasive bladder cancer (MIBC) across humans, canine, feline, and bovine species. They sequenced the largest non-human MIBC exome data (n=118) for tumor-normal pairs and did comprehensive somatic mutation analysis (mutational burden, mutational signatures, and driver gene discovery) by comparing data to previously published human MIBC cohort (n=412). Key findings from this study were: 1. Cross-species convergence of somatic driver mutations and focal syntenic copy-number changes in an otherwise dissimilar somatic mutational load (coding mutations per Mb of genome) observed in this comparative analysis, and 2. Identification of an environmental exposure (bracker fern (BF)) induced unique mutational processes in all eight of geographically separated bovine cases with in vitro cell line experiments suggesting potential carcinogenic activity in humans exposed to either BF extracts or its purified metabolite, ptaquiloside. This study involved significant collaborations across 17 countries to acquire tissue specimens and authors have performed extensive comparative analysis while ensuring rigorous implementation of methods towards somatic variant calling, significance test for somatic variants, including copy-number segments. This study is of much relevance to highlight value of comparative genomics of studying spontaneous cancers, especially to characterize conserved cancer drivers and underlying mutational processes that are observed across species.

We sincerely thank the reviewer for their appreciation of the scale of the project and the importance of its findings.

I have a few concerns related to mutational burden and mutational signature analysis.

1. For mutational burden analysis, are mutations comprise of single-nucleotide variants (SNVs) or also include SNV with indels? If the latter, is there a significant difference in bovine vs. canine + feline cohort when counting numbers per Mb for SNV vs. indels?

For the mutational burden analysis we performed, the mutations comprised both SNV/MNVs and indels. However, we have now calculated the median rates for each species (in mutations/Mb), and this information has been added to the text (lines 180-183):

| **Species** | **SNV/MNV rate** | **Indel rate** |
| --- | --- | --- |
| Canine | 0.94 | 0.074 |
| Feline | 0.96 | 0.18 |
| Bovine | 51 | 11 |

All three species show that the majority of the mutations come from SNV/MNVs rather than indels. The canine and feline samples have approximately 10-fold more SNV/MNVs than indels, and the bovine samples have approximately a 5-fold increase. The majority of the indels in the bovine cohort can be attributed the novel indel signature we identified (Figure 5), which we have shown can be recapitulated *in vitro*, whereas in the canine and feline cohort the indel rate is only elevated in a few samples with MMR deficiency.

I believe exome capture kit was custom tailored for each of canine, feline, and bovine genes, and somatic variants were filtered for FFPE artifacts. If so, do those artifacts show relative bias in bovine vs. other species? If not, much higher observed coding mutational burden in bovine UC cases is biologically intriguing even after factoring for BF extract induced mutatgenesis.

The reviewer is correct in that we designed bespoke exome baits for each of the three species and somatic variants were filtered for both FFPE artefacts (C>T) and C>A artefacts. In terms of relative bias in the artefacts seen in the bovine samples versus the other species, our experience of sequencing DNA from FFPE tissues (of human, dog, cat, cow, horse and mouse tissues) is that the frequency of FFPE-induced artefacts can vary greatly, even in samples from the same species. Typically differences in the number of FFPE-induced artefacts between samples is influenced by a number of factors during the fixation process that are related to formalin-induced fragmentation of the DNA and the storage of the FFPE blocks. For example, the use of lofw pH formalin increases the rate of apurinic/apyrimidinic site formation over time, and eventually leads to degradation and fragmentation. In addition, long-term storage of formalin-fixed blocks can also induce fragmentation due to exposure to environmental conditions (reviewed in PMID: 24885028).

2. For recurrent hotspot driver mutations, do these driver events vary or enriched within a subset of cohort, e.g., driver events could vary across low vs. high mutational burden samples, and if so additional comparative analysis by factoring in different mutational burden could help identify driver events in say MMR-deficient vs. MMR-intact cohort?

This is an interesting suggestion that could indeed add more insight into the driver events in canine and feline UC. Unfortunately, we only have evidence for MMR-deficiency in two canine tumours and one feline tumour, so there is not enough statistical power to draw any meaningful conclusions from the findings at this time.

3. Minor: Line 239-240: Unclear why authors state "appeared to be" biallelic inactivation of MSH2. Could this be supported with PCR/RNA-Seq if tissue specimen is still available? If not, statement provides less clarity and more confusion to readers.

In tumour sample DD0194a, we identified a somatic frameshift mutation in *MSH2*, with a variant allele frequency of 0.87, which indicates there was a loss of heterozygosity through deletion or copy-number neutral LOH. Further, there are 4 other somatic variants within 3.5kb upstream of *MSH2* with VAFs 0.89 to 0.96, and somatic copy number analysis indicates that there is a ~1.2Mb deletion in the region, with the predicted boundary only 50kb from the *MSH2* frameshift mutation. Given that we are using exome sequencing data to call copy number changes, which has lower resolution, it is likely that the exact boundaries of the deletion are larger and explain the VAFs of these mutations. We have altered the text to provide some additional details: “In tumour sample DD0194a from another dog, we can predict that there has been biallelic somatic inactivation of *MSH2*, through frameshift mutation and loss of heterozygosity, as the allele frequency (AF) of the frameshift was 0.87 and this and 4 other upstream mutations have an AF of between 0.89 and 0.96 and fall near or within a predicted >1Mb deletion.”

Unfortunately, we were unable to verify this with PCR or RNA-seq as we do not have additional material from this case.

4. Since mutational signatures, especially de novo ones in bovine cases were based on exome and not whole-genome data, authors should state such limitation of inferring genome-wide signatures based on exome data alone and could have provided additional support using whole-genome dataset, e.g., possible if a published whole-genome dataset exists for bovine cases (normal or tumors).

The bovine UC cases have very high mutation rates (51 SNV+MNV/Mb and 11 indels/Mb). While we feel the high SNV mutation rate provides sufficient data for SBS signature analysis (~950-4000 SNVs per sample), it is true that a greater number of mutations from genome-wide sequencing would substantially increase power for signature extraction, particularly DBS and ID signatures, as indel and MNV mutation rates are lower. Alternatively, a greater number of exomes would also increase power. We are not aware of a publicly available whole-genome dataset that exists for bovine UC tumours. We also note that whole-genome sequencing might be beneficial for signature analysis in feline and canine UC, where mutation rates are much lower. We have added the following text to the manuscript to address your comment, in the context of the discussion involving the APOBEC activation mutational signature: “While a subset of human MIBC with a high mutational load are associated with APOBEC activation signatures [22], we did not find elevated mutation rates in feline or canine UC cases other than those associated with MMR deficiency. Examination of larger cohorts may be required to confirm that APOBEC activation is not a mechanism of mutagenesis in canine and feline UC. Although *de novo* signature extraction was limited by the low number of mutations available for analysis, it is unlikely that APOBEC-mediated mutagenesis was active, given the very low mutation rate observed. Nonetheless, performing whole-genome sequencing or collecting much larger WES data sets may enable discovery of other signatures that contribute to a lower mutational burden.”

5. Given the study involves detailed comparative analysis, for each of major sections showing key findings, readers may find it easier to navigate the paper if each section starts with rationale and ends with a key one to two findings and highlighting biological/clinical relevance and move numbers to supplementary methods.

We understand the reviewer’s concerns and have added additional sentences to the Results sections to help the reader with our rationale and the relevance of our findings. We have also broken up one of the Results subheadings into two parts, to help with clarity.

However, we would prefer to leave the numbers in for clarity (which is also consistent with the journal’s style).

**Reviewer #2**:

This article by Wong and colleagues presents an interesting data set of whole exomes from canine, feline and bovine urothelial carcinomas (UC). The authors compare driver mutation profiles, mutational signatures and somatic copy number alterations with public human UC data and describe noteworthy similarities and differences among species. They also investigate whether an unknown mutational signature found in bovine tumors can be explained by bracken fern mutagenesis through a series of in vitro experiments.

Overall I found this paper very pleasant and straightforward to read. I have no significant concerns from a technical point of view, the sequencing data analysis is evidently well done, it recovers some known features (e.g. of canine tumors) and points to some new observations.

We sincerely thank the reviewer for their appreciation of our manuscript and findings.

From a narrative point of view, I think the authors could improve the impact of the manuscript if they challenged themselves to explain in more detail how cross-species analysis could contribute to our understanding of human tumors. If we observe similarities/differences in driver gene and/or SCNA frequency among species, what exactly does that teach us about the biology of UC?

Advances in genome sequencing have enabled comprehensive cataloguing of mutations and copy number events in cancer. However, distinguishing between passenger and driver mutations remains challenging. Cross-species analysis is one method that can contribute to advancing our understanding of tumourigenesis.

Firstly, identification of novel cancer genes in other species can aid in identification of driver genes in humans. There are several examples of cancer genes/pathways that were identified through cross species analysis; first identified in mice (e.g. PMID: 16814709, 15608639, 16814714) or dogs (e.g. PMID: 20028558), then subsequently investigated in humans and found to play an important role.

Secondly, identification of significantly mutated genes (SMGs) or SCNAs that are common between species can help to identify key driver genes from longer lists of candidates. For example, WES of 412 human muscle-invasive bladder cancer samples identified 58 SMGs. Two of these genes were SMGs in canine bladder UC and 3 were SMGs in feline bladder UC, thus, these genes could be prioritised in human (and animal) UC, given their conserved role in bladder UC tumorigenesis across species. Thirdly, elucidation of the similarities and differences in the oncogenomic landscape of bladder UC in non-human species allows us to determine if an animal represents a relevant model. It is fair to say that no single animal model by itself will be entirely faithful to the disease, thus, multiple models, such as dogs and cats, alongside the more traditional models of mice and zebrafish, can provide valuable insight into the disease.

Thus, similarities between human and canine/feline tumours affords opportunities via a comparative oncology approach, as a means to improve and expedite our understanding of cancer biology and potential therapies (such as through the outcomes of canine clinical trials; PMID: 36658200).

New text has been added to the beginning of the Discussion section: “Advances in genome sequencing have enabled comprehensive cataloguing of mutations and copy number events in cancer. However, distinguishing between passenger and driver mutations remains challenging. Cross-species analysis is one method that can contribute to advancing our understanding of tumourigenesis. Firstly, identification of SMGs or SCNAs that are common between species can help prioritise and refine candidate driver genes and potentially refine our mechanistic understanding of cancer gene function. Secondly, elucidation of the similarities and differences in the oncogenomic landscape of tumours in non-human species allows us to determine whether they represent relevant models that can be utilised as a means to improve and expedite our understanding of cancer biology and potential therapies.”

Do the authors have any hypotheses to explain the notable differences (BRAF mutation frequency in canines, lack of TP53 etc.)?

In the Discussion section we noted that *MDM2* was significantly amplified in canine UC. As *MDM2* is a negative regulator of *TP53*, amplification of *MDM2* is an alternative mechanism leading to the disruption of the p53 pathway in canine UC. Additionally, *RB1*, which is frequently co-mutated with *TP53*, was also significantly deleted. We have altered the wording of this section and moved it to follow the discussion of *TP53* and *RB1* in cats and human: “Mutation of *TP53* was notably absent in canine UC, however, like human MIBC, *MDM2*, which is a negative regulator of *TP53*[67] and *RB1*[68], was significantly amplified in canine UC (**Fig. 3**), and *RB1* itself was also significantly deleted. This suggests that disruption of the p53 pathway, through amplification of *MDM2* rather than *TP53* mutation, may be a key driver of tumourigenesis in canine UC”. Additionally, we have modified Figure 3 to show the canine UC samples in which *MDM2* is amplified.

While we are the first to report on the genetics of feline and bovine UC, the genetics of canine UC has been reported previously, albeit by the analysis of smaller cohorts. From these studies it has been determined that *BRAF* is frequently mutated in canine UC (references 25-28). We have communicated with tens of veterinarians throughout the course of this project, and none of them have been able to offer any suggestions/insight as to why *BRAF* is so commonly mutated in canine UC, in stark contrast to the other species. As we currently do not understand why there is a selection for different driver genes in different species (and tumour types), we do not feel in a position to speculate in the manuscript.

Did the results of their analysis yield what the authors were hoping for at the outset of the work - why or why not?

The goals of this project were to several-fold. Firstly, we wanted to elucidate the similarities and differences of bladder UC amongst different species, and leverage this to make further insights into understanding the drivers of bladder UC in humans. Secondly, we wanted to provide insights into the genetics of canine and feline bladder UC for the benefit of these species themselves, by allowing a better understanding of the biology of the disease and also to inform treatment options. Lastly, we wanted to provide genetic evidence that ptaquiloside from bracken fern, long considered a potential carcinogen, is involved in bovine bladder UC, which has implications for humans (who consume this fern, in particular parts of the world). We very much feel that we achieved our goals.

What could be the reason for the lack of SCNAs in bovine tumors?

A possible reason for the lack of SCNAs could simply be that the mutagen functions by inducing point mutations and indels (ptaquiloside preferentially alkylates adenine bases in specific sequence context) at a high rate, and once the mutagen has generated sufficient SNV/MNV/indels for the mutagenic process to occur, there would be no need to generate additional genomic aberrations such as SCNAs. Indeed, analysis of TCGA data from 12 different cancer types has shown a striking inverse relationship (independent of tumour tissue of origin) between the number of recurrent SCNAs and the number of somatic mutations (PMID: 24071851).

This has now been added to the text in the Results section ‘Cross-species comparative analysis of somatic copy number alterations: “The lack of SCNAs in bovine UC is in line with the previous observation that there in an inverse relationship between the number of somatic mutations and SCNAs[49]. Additionally, PT preferentially alkylates adenine bases[50] leading to small DNA aberrations rather than genomic instability.”

Currently the text is very technical, essentially a long list of similarities and differences. Giving it more biological/interpretive depth would benefit the article.

As requested, we have added more biological/interpretive comments at various points throughout the Results and Discussion sections.

I do have one scientific concern with respect to the section in which the authors perform in vitro experiments to ascertain whether the mutational signatures recovered from bovine tumors are caused by bracken fern. First of all, this section is very hard to follow, as all the signatures have been given relatively similar names. But more importantly, it is not clear to me whether the BF in vitro experiment really recovered the bovine signature as the authors claim it did. Throughout the text, the authors state that if newly extracted signatures have a cosine similarity of less than 0.9 to existing signatures, they are considered unrelated. However, when comparing e.g. BFA-A and bovine signature BF-A, the authors conclude "strong resemblance" (line 306) based on a similarity of 0.82. By their own measuring stick this similarity should be considered weak. The same problem arises for comparison of the PT signatures and the bovine signatures. Again, the text is confusing due to an abundance of similar acronyms but it did not really clarify for me whether the authors had indeed proven that bracken fern was the origin of the bovine signatures.

We have added text to help clarify whether we are referring to the signature found in the bovine bladder tumour samples (specifically the ‘bovine UC signature BF-A or BF-B’) or the signature found in the human bladder cancer cell line (specifically the ‘*in vitro* Signature BFA-A or BFA-B’’ to indicate the signature found from the bracken fern extract experiment and ‘*in vitro* Signature PT-A or PT-B’ to indicate the signature found from the PT experiment).

We have also included an additional figure, Additional file 11: Fig. S5, in the supplementary materials. The aim of this figure is to visualise the bovine UC and *in vitro* signatures alongside the known signatures from COSMIC and Signal with the highest cosine similarity. This figure shows that both cosine similarity and visual inspection are necessary to judge the degree to which signatures resemble each other. We have also altered the main text (‘**Characterisation of a bracken fern-associated mutational signature’** section) to provide specific details of cosine similarities and differences and similarities from visual inspection:

“There was no confident match with any known mutational signatures, as the highest cosine similarity between bovine Signature BF-A and a COSMIC or Signal[44] signature was only 0.49 and 0.63, respectively, and for bovine Signature BF-B, the highest cosine similarities were 0.68 and 0.73, respectively. Visual comparison of the spectra also confirmed the matches as low confidence (**Additional file 11: Fig. S5**)”…………….“Using mutations pooled from all BF extract doses and time points, mutational signature extraction was performed (see **Methods**). Based on goodness-of-fit (see **Methods**), it was estimated that two SBS signatures, which we designated *in vitro* Signatures BFA-A and BFA-B, were active in the human UC cells treated with BFA (**Fig. 6a**). The relative exposure of each signature within each individual sample is shown in **Additional file 15: Table S7**. The cosine similarities between the observed mutational spectra in the BFA treated cells and the mutational spectra reconstructed from the extracted signatures at each dose and time point were between 0.974-0.992. An example from cells exposed to BFA for 3 days (at IC_50_) is shown in **Additional file 17: Fig. S9a**. The *in vitro* Signatures BFA-A and BFA-B highly resembled the two *in vitro* SBS signatures active in cells treated with BFE (cosine similarity = 0.94 and 0.98 to Signatures BFE-A and BFE-B, respectively; **Additional file 17: Fig. S9b-c**), henceforth, the discussion will focus on the signatures identified in cells treated with BFA. *In vitro* Signature BFA-A did not confidently match any known mutational signature, with the highest cosine similarities being 0.7 to COSMIC SBS25 and 0.74 to Signal SBS141 (**Additional file 11: Fig. S5a**). Additionally, a visual comparison of the mutational spectra for COSMIC SBS25 and Signal SBS141 do not contain the T>C and T>A peaks in specific sequence contexts, which are the distinguishing feature of the novel Signature A observed in bovine UC and *in vitro*. Signature BFA-A had a good resemblance to the bovine UC Signature BF-A that we identified (cosine similarity = 0.82), with a prevalence of T mutations in specific trinucleotide contexts (**Fig. 6a**). *In vitro* Signature BFA-B did not resemble bovine UC Signature BF-B (cosine similarity = 0.57). *In vitro* signature BFA-B had cosine similarities of 0.84 to 0.87 to three very distinct known signatures, COSMIC SBS40, Signal SBS18 and Signal SBS167, which is annotated as tentative or having a lack of evidence (**Additional file 11: Fig. S5b**), which provides some uncertainty as to whether any of these are true matches. In summary, there is a good resemblance between the bovine UC Signature BF-A and the *in vitro* Signature BFA-A that was seen in the BF extract-treated human UC cell line, and these signatures do not resemble any COSMIC or Signal signatures. The number of indels found in BFA-treated cells ranged from 108-193 and we did not find a propensity toward deletion of T in CTG or CTC sequence context as we did with bovine UC.”

**Reviewer #3:**

The authors perform a cross species comparison of bladder cancer using WES of canine (n=87), feline (n=23) and bovine (n=8) matched tumor-normal pairs.

They use human WES data was from TCGA study (Robertson et al. Cell, 2017) from 412 cases. They also highlight the role of a specific carcinogen (ptaquiloside).

The study is technically robust and provides interesting new insights.

We sincerely thank the reviewer for their appreciation of our study.

We suggest to address the following issues:

1. Rationale for this study.

The authors mention that one goal of the study is to identify key driver genes and to refine cancer driver genes in human cancer (e.g. line 149). Even though we agree that comparison across species is an interesting basic research question, there are other strategies to identify human cancer drivers that may be more relevant. We would suggest to tone down this particular goal, as other approaches to find human cancer driver genes seem more appropriate.

We appreciate the reviewers’ perspective, however, we are simply presenting one of many strategies to identify driver genes, not suggesting it as an alternative. Of note, cross-species oncogenomics has been applied with success across various species to identify human cancer drivers in a high throughput manner, including zebrafish (reviewed in PMID: 26070506), mouse (e.g. PMIDs: 20103622, 25965574, 28463229), dog (e.g. PMIDs: 20028558, 31440272, 32680987, 33649193, 26056372), and combinations of species, such as dog/horse and dog/mouse (e.g. PMIDs: 30093633, 30664638).

The authors argue that cat and dog models have advantages as compared to mouse models because they share the same environment as humans. However, the exposure and risk factors are very different between humans and animals. Even though they share the same environment, humans smoke, drink, have an unhealthy diet, get exposure at their workplace etc, which play a critical role in human cancer (not addressed here).

We understand the reviewer’s comment that pet dogs and cats may not entirely share the same scope/spectrum of environmental exposures as humans, however, they do share far more than with laboratory mice. It is important to note that pet dogs and cats get exposed to carcinogens that humans are commonly exposed to, such as second hand smoke and air pollution. In terms of workplace and other exposure, there are links between exposure to pesticides in pet dogs and cancer, including bladder cancer (PMID: 15112777, 22222006), and a study has shown that people and their dogs have similar pesticide exposures in a home environment (PMID: 34964617). With regards to diet, obesity is the most common nutritional disease in dogs, which is associated with inflammation and cancer (reviewed in PMID: 36262532). Thus, the evidence suggests that pet dogs (and cats) do represent relevant animal models.

2. Relevance of the psaquiloside signature in humans.

Chronic ingestion of braken fern is not really relevant in humans. As the psaquiloside signature is not detected in humans, it seems like exposure from the milk or groundwater may not be sufficient.The relevance of ptaquiloside exposure in humans is questionable. If this would be a carcinogen it would be important to investigate the role of this carcinogen, but from the presented data the evidence is limited.

Bracken fern (BF) is suspected to cause human cancers and is classified by the WHO/IARC as *‘possibly carcinogenic to humans*’, due to links between oesophageal and gastric cancer and BF/ptaquiloside (PT) exposure (references 74-77). In addition, bracken and PT have been placed on the WHO/IARC urgency list of potentially carcinogenic compounds/products to be re-evaluated. As we mentioned in the Discussion, humans are exposed to PT when ingesting the bracken fern itself as a food source, which is common in East Asia in stir-fries, soups, and stews (PMID: 36850990). Similarly, consumption of milk or meat from cows that have consumed BF (as reported in Venezuela (PMID: 11146454) and Korea (PMID: 36977122)), consumption of drinking water from PT-contaminated groundwater wells (as reported in Europe (*Environmental Sciences Europe, Vol. 33, Article no. 44, 2021*)) or consumption of bracken-based traditional medicine (e.g. traditional Chinese medicine; PMID: 34458862). We cannot say whether the PT signature is detected in humans or not, as we are not aware of any tumours sequenced from individuals known to have had long-term exposure to PT.

3. Differences in signatures across countries/regions.

It would be interesting to search for differences in signatures between animals and humans from different regions and countries, also between cows that are outside or kept inside. The signatures might also be interesting outside the context of bladder cancer (e.g. If the spores play a role, as suggested by the authors, then lung cancer might be interesting).

It is an interesting suggestion to see if there would be any variations in the novel signature we identified when looking across different regions or countries. However, the data we have is already from three different geographical areas across 2 different countries (see Methods), and so, although our numbers are small, we still find the signature is consistent amongst the samples.

Cows do not typically develop bladder UC in the absence bracken fern consumption, so it would be extremely difficult to assess the differences in mutational signatures between animals kept outside (where they have free access to consume bracken) and those kept inside (which would not have exposure to bracken), as the chance of the latter group developing UC would be small-to-none.

It was not suggested by the authors that bracken spores can play a role in cancer development per se; we quoted a reference which found that mice fed bracken fern extract or bracken spores developed tumours in their upper gastrointestinal tract tissues (reference 84). While we agree it would be interesting to look at signatures caused by bracken fern outside the context of bladder cancer, this is out of the scope of this paper.

4. Cancer drivers.

Line 470-475: were these drivers not already known before in bladder cancer? It seems like the vast majority was already known (e.g. KDM6A, TP53, NRAS, MDM2, RB1 reported). Are there any novel drivers that were identified by this study? If so, emphasiing these and role of these novel drivers might strengthen the study. One potential new tumor suppressor gene (ARHGEF10) is selected, validations or further analyses on the role of this gene may be interesting.

As large-scale sequencing studies have been performed on cohorts of human MIBC, established human bladder cancer driver genes have previously been identified, along with a long list of candidate drivers. *ARHGEF10* has been suggested to be a putative tumour suppressor in several human cancer types, including UC, where reduced expression has been reported in >50% human UC cell lines (reference 56). Our findings help to confirm this gene has an important tumour suppressor function in human and animal cancers, and suggest that indeed, *ARHGEF10* should be further characterised. Some functional work on *ARHGEF10* has been performed (e.g. PMID: 31477830, 28448737) and further functional analysis is out of the scope of this paper.

5. The authors argue that cat and dog tumors can offer an animal model.

Even though heterogeneity may be an advantage, spontaneous tumors are hard to use to test treatments in terms of logistics, due to the need to wait for the development of these tumors. There may be applications but limited

We understand the reviewer’s concerns but would again like to stress that pet dogs and cats really do represent a valid animal model. The time for development of spontaneously arising tumours in pet dogs and cats is longer than would be in the mouse, however, this in of itself represents a more faithful recapitulation of the human disease.

Bladder cancer comprises ~1.5–2% of all naturally-occurring cancers in dogs (a rate similar to that reported in humans; PMID: 32039002)), and there are an estimated population of 65.1 million pet dogs in the USA/11 million in the UK. Thus, this represents a large pool from which a potential cohort for analysis could be obtained. Indeed, a study published earlier this year demonstrated the feasibility of using pet dogs with cancer enrolled in clinical trials to assess the effectiveness of targeted therapies (using drugs designed for humans), and dogs with bladder UC represented the second biggest tumour cohort in this study (PMID: 36658200). Further, the NCI/NIH fund the PRECINCT programme with the expressed aim of developing immunotherapies for canine cancers that can then be trialled in humans (https://www.vet.upenn.edu/about/news-room/news-stories/news-story-detail/nih-funded-canine-immunotherapy-data-center-charts-a-path-toward-transformative-therapies).

6. Experimental validation of the signature.

Inducing the mutational signature on normal cells and not on cancer cells would be more interesting. Phenotypically: what else happens to the cells treated with the carcinogen? Any consequences on the transcriptome?

We agree with the reviewer that testing for an induced signature in normal bladder cells would have been interesting and it was something that we discussed. While we have patient-derived normal human urothelial cells, they are challenging to sustain in culture and to bulk up sufficiently for experiments. They do not respond well to either low or high density seeding and have quite a high propensity to senesce. The need to first perform chemosensitivity assays (on different days) to determine what concentrations of extracts and ptaquiloside would be appropriate to use (non-toxic dose/IC_20_/IC_50_ determination) and then follow on experiments scaled up to T25s at 3, 7, 10 and 14 days for DNA extraction meant using these cells was not feasible.

Using the human bladder-derived KU19-19 cell line, down the microscope there were no obvious phenotypic effects from the treatments, although quantitative studies were not performed. Despite the lack of an obvious phenotype, given the induced mutational signatures, we agree that analysis of transcriptome changes may be interesting. However, given the limited quantities of bracken extracts available which was prepared from fresh seasonal fronds and of ptaquiloside, it was only possible to treat cells for mutational signature analyses. In addition, the intention of the cell line experiment was to determine if we could recapitulate what we were seeing in the bovine tumours in terms of the DNA mutational signature, thus we focussed on these studies.

7. Could the authors comment on the absence of APOBEC signature and on the fact that the mutational load is not as high in animals?

It was revealed previously that the somatic mutation rate differs significantly among species and is inversely correlated with their lifespan [Nature 604, 517–524 (2022)]. However, it remains unclear whether the author considered this factor and employed an adjusted mutation rate for species comparison.

While APOBEC-AID signatures 2 and 9 have been reported in canine gliomas (PMID: 32049048), we did not see any evidence of these signatures in the canine bladder UCs in our study. The absence of an APOBEC activation signature, however, should be confirmed with further examination of larger cohorts, as APOBEC-mediated mutagenesis may be active in a smaller subset of canine and feline UC. We have included the comment below in the main text to address an analogous question from Reviewer #2: “While a subset of human MIBC with a high mutational load are associated with APOBEC activation signatures [22], we did not find elevated mutation rates in feline or canine UC cases other than those associated with MMR deficiency. Although *de novo* signature extraction was limited by the low number of mutations available for analysis, it is unlikely that APOBEC-mediated mutagenesis signatures are present, given the very low mutation rate. Nonetheless, performing whole-genome sequencing or collecting much larger WES data sets may enable discovery of other signatures that contribute to a lower mutational burden.”

The Nature paper entitled “S*omatic mutation rates scale with lifespan across mammals*” investigated rates of somatic mutation across species in normal tissue (intestinal crypts) in relation to ageing and cancer risk over a lifetime, not tumour tissue. Therefore, the link between this study and the work presented in our manuscript is unclear.

Furthermore, there is insufficient evidence in this paper to support the claim that the elevated mutation rate observed in bovine is attributable to a potent environmental mutagen.

There are several factors that strongly point to bracken fern/ptquiloside being causal of the high mutation load and signature we observed:

- Bracken fern contains ptaquiloside which has been shown to be mutagenic and carcinogenic in numerous studies using a variety different experimental methods (e.g. PMIDs: 3328033**,** 9514907, 9753659, 11827712, 2957538, 3690048).
- It is well-established that cattle fed on a diet of bracken fern develop bladder cancer (e.g. PMID: 28559101, 5723967, 15737346, and references pre-PubMed indexing, including Pamukcu, 1963; Pamukcu et al., 1966).
- The veterinary pathologists who contributed the bovine samples confirmed the cows had been raised in bracken fern endemic areas and that the cows had grazed on the ferns.
- The point mutations in bovine UC were primarily T/A mutations in a very specific sequence context, which is in line with previous studies indicating that ptaquiloside preferentially alkylates adenine bases (references 32 and 33). This SBS signature is not in the COSMIC or Signal databases and thus cannot be explained by other exposures.
- Indels rates were also elevated in bovine UC, and indels also primarily involved deletion of single T/A bases. The indels predominately fell in the sequence context CTC or CTG, and not in polyT/A regions, which excludes mechanisms such as defective mismatch repair as a cause of the elevated indel rate. This ID signature is not in the COSMIC or Signal databases.
- Although fewer dinucleotide variants were identified, almost all involved mutation of T/A bases.
- We recapitulated the the bovine UC signatures *in vitro* after exposing cell lines to bracken fern extract and purified ptaquiloside.

8. Methods

For canine and feline data: the authors feed existing signatures

For bovine tumors they do de novo discovery

It would be good to do de novo discovery as well on canine and feline tumors for consistency.

Signature extraction was performed on feline and canine mutational catalogues (data was not shown), however, because mutation rates in the majority of samples was low, power to extract signatures was low and almost all reconstructed spectra had a cosine similarity < 0.75 to the original spectra in the feline cohort and < 0.80 in the canine cohort. Visual inspection of the mutational spectra from extracted signatures indicated that there was likely a combination of SBS signatures that SigFit was unable to deconvolute. As such, we performed SigFit signature fitting (which runs on individual samples), on the samples that were observed to have a much higher mutation rate than others in their respective cohorts to determine if additional signatures were present. We have added additional details to the Methods section to indicate that signature extraction was also performed for feline and canine samples: “Signature extraction was performed using 10,000 sampling iterations and a goodness-of-fit plot was generated using the SigFit functions ‘calculate_gof’ and ‘plot_gof’ to estimate the optimal number of signatures. Signature re-fitting was then performed using the extracted signatures in order to calculate the signature exposures per sample. Signature extraction was performed in the same manner for the mutational catalogues from canine and feline UC, converting mutational counts relative to the human genome using mutational opportunities calculated from the canine and feline exome plus 2bp flanking each exon. Signature extraction was performed for mutational catalogues from the human urinary bladder cells as described above, without conversion of the catalogue counts.”

There is also a lack of detail regarding the de-novo estimation of mutational signatures in bovine samples. The authors mention that they used goodness of fit plots to select the number of signatures. Ideally, such plots should be provided in supplement, or at the very least, more details should be provided about how goodness of fit was evaluated. Please also provide the code for the analyses.

Assessment of goodness-of-fit is provided as part of the Sigfit package, and therefore we do not have any custom code to provide. We used the ‘calculate_gof’ and ‘plot_gof’ functions as described in the Sigfit documentation here:

<http://htmlpreview.github.io/?https://github.com/kgori/sigfit/blob/master/doc/sigfit_vignette.html>

In all cases the best estimate of the number of signatures calculated by the algorithm was 2. We have added additional text in the Methods to indicate the above.

9. Statistics missing for signatures

Goodness of fit plot for signatures are missing

There is not so much information on the exact statistical analysis used. For example:

Line 618-619: please write down which statistical test was used.

Line 626: please write down which statistical test was used.

The statistics we used have all been provided by the tools themselves, which we have cited in the manuscript, so we do not feel it is necessary to describe them again in our Methods section.

- As above, the goodness-of-fit assessment is part of the Sigfit package and we used the estimates suggested by the algorithm.
- The derivation of the P-values provided by STAC is described in detail in their publication (https://www.ncbi.nlm.nih.gov/pmc/articles/PMC1557772/) so we do not feel it is necessary to reiterate the methods in our paper. A reference to the publication describing STAC has already been provided in the manuscript. We have added a line in the Methods to indicate the P-value is calculated by STAC.
- Similarly, the SMG test and FDR calculation is derived from MuSiC2 and described in the original MuSic publication (<https://www.ncbi.nlm.nih.gov/pmc/articles/PMC3409272/>). We have added a line in the Methods section to refer readers to the MuSiC publication for the statistical analysis.

If 15 genes are identified as significant in 1 species and 12 genes in another species

How many are in common, and what is the chance of that happening randomly

As algorithms to identify SMGs are different from calculating the probability of overlapping events, and different species may have different background mutation rates, it is not possible to provide a meaningful answer to this question.

LRP1B identified in a cow with 2 tumors

The authors state that it is a driver because identified in both

Maybe just hit twice independently, just a passenger

Based on n=2, hard to tell this is a driver

Strong statement

Effect of the mutation?

The effect of the mutation is stated in the main text, S2686P, and is a missense mutation. We have changed the statement in question to “...the only mutation they shared was in putative tumour suppressor *LRP1B* (p.S2686P), implicating this as a driver mutation in the tumours from this cow”. However, we do discuss in the manuscript that *LRP1B* has already been identified as a putative driver gene in UC and other cancers, and *LRP1B* is also significantly mutated in the feline and canine cohort and recurrently mutated in the bovine cohort. Our cross-species comparison shows that *LRP1B* is indeed a key driver gene in UC, as significant mutation of this gene is conserved across multiple species.

10. Germline variants

Discovery algorithm only used among somatic mutations

Do the authors identify any germline variants? Only the prevalence of germline variants is reported.

Do specific germline drivers co-occur with somatic drivers? Predispose to?

Mutual exclusivity and co-occurrence are evaluated among somatic mutations. Was there any co-occurrence or exclusivity detected with germline variants?

As the discovery algorithms used for somatic mutations are not suitable for germline variant analysis, we searched for candidate germline predisposition variants by calling all variants in the germline and identifying frameshift and nonsense variants in COSMIC CGC genes orthologs (see section “Germline predisposition variants'' and Methods), as these variants are more likely to have a detrimental effect on gene function. For more in-depth analysis such as co-occurrence or mutual exclusivity, we would require more comprehensive lists of SNPs in each species, in order to eliminate common SNPs from the lists of variants called. Although we do have lists of some common SNPs in cat, cow and dog (see Methods), the data is too limited to completely eliminate common SNPs for downstream analyses. Additionally, in genes not previously associated with cancer, it is difficult to predict if a germline missense mutation, for example, confers susceptibility to cancer.

11. CT scoring and copy-number calling

There is not sufficient detail provided about how the chromothripsis scoring was done. Voronina et al. was referenced for the method of scoring, however, the referenced manuscript did both manual and computer assisted scoring. Additionally, the scoring in Voronina et al. was done after copy number calling following alignment of all reads from the exome sequencing, ignoring the baited regions. Was a similar method employed by the authors?

It would be necessary to provide a more detailed explanation of the methodology used to estimate somatic copy number alteration. Specifically, the reference genome, window size, and normal control samples should be further elucidated. Additionally, it is unclear how the authors identified and excluded samples with excessive noise; it is imperative to outline the selection criteria employed in the analysis.

The results from Sequenza copy number calling were used to estimate the prevalence of chromothripsis. Sequenza uses aligned reads as a standard input and generates alleles-specific genomic segments. We have included additional details in the Methods sections “Somatic copy number alterations” and “Chromothripsis” (indicated in red below):

**Somatic copy number alterations**

Sequenza (v3.0.0)[99] was used to identify allele-specific SCNAs from aligned tumour and matched normal sequence reads (described above). All results were manually curated, and, where applicable, alternative ploidy and purity estimates were used to replace the default best-fit solutions, as previously described[99]. While Sequenza does not provide a specific measurement to evaluate noise and the quality of the estimates, various plots are provided for this purpose. Manual curation included visual inspection of the model fit plots, which show the correlation of the B-allele frequencies (BAF) and depth ratios with joint log posterior probability (LPP) density, the contours plot, which show the LPPs of a range of ploidy and purity combinations, and genome plots showing BAF and depth ratios, as described in the Supplemental Materials of Favero *et al*.[99]. Samples that were deemed to have excessive noise were excluded from CN analysis. Sequenza provides absolute copy number calls for segments, therefore, to determine if a segment represents a relative CN gain or loss, the tumour ploidy was first determined as the most frequent CN assigned to segments with mean B-allele frequencies > 0.3, and a CN gain or loss was called if the absolute CN of the segment was above or below the ploidy, respectively. STAC[54] was used to find significant CN gains and losses in the canine and feline samples. Regions of gain or loss with frequency p-value < 0.05 or footprint p-value < 0.05 were considered statistically significant. The derivation of the frequency and footprint p-values are described in the STAC publication[54].

**Chromothripsis**

We used the definition and scoring of chromothripsis as outlined in Vorinina *et al*.[53] to estimate the extent of these events in the canine and feline tumours from Sequenza CN segmentation results (see above). Regions positive for chromothripsis were defined as high-confidence (10 or more CN state switches in 50Mb), intermediate-confidence (8 or 9 CN state switches in 50Mb) or low-confidence (6-7 CN state switches in 50Mb) If positive, chromothripsis was further classified as canonical (2 or 3 CN states) or non-canonical (>3 CN states). For chromosomes < 50Mb, the number of required CN state switches was scaled accordingly, and rounded to the nearest whole number. For example, if a chromosome is 30Mb, a scaling factor of 0.6 (30/50Mb) was applied to the definitions of high-confidence (6 or more CN state switches), intermediate-confidence (5 CN state switches) and low-confidence (4 CN state switches) regions of chromothripsis.

12. When they look for similarity to existing signatures, the authors use a cosine cut off 0.9

But between bovine and treated cell lines: the cut-off lowered to 0.82

A consistent cut off would be better

Reviewer #2 also noticed this inconsistency, and we have altered the text to provide additional details on cosine similarity and visual inspection of mutational spectra to judge how well signatures resemble each other. We have also added Additional file 11: Fig. S5 to aid in visualisation of the best matches between the signatures found in bovine UC, *in vitro* and known signatures.

13. Figure 6

Comparison of the signatures to COSMIC signatures

It would be nice to see the full comparison, also to understand the cut off of 0.9

We have provided an additional figure in the supplementary materials, Additional file 11: Fig. S5, to show visually the comparison of the novel signatures we describe compared to COSMIC and Signal signatures. The figure shows the highest matching known signature for each of the bovine UC and *in vitro* signatures. It is evident that the mutation profiles for Signature A (in bovine UC and *in vitro)* are quite different from the highest matching known signatures, as the known signatures lack the peaks in the context-specific T>A and T>C mutations, and have cosine similarities of 0.78 or lower.

With bovine Signature B (BF-B), which was different to Signature B found *in vitro*, the highest cosine similarities were to COSMIC SBS40 (with unknown aetiology), with a cosine similarity of only 0.67, and signal Signal SBS161 (annotated as tentative of lack of evidence of existence) and SBS100 (not annotated), with cosine similarities of 0.73 and 0.72, respectively). The *in vitro* signature B had higher cosine similarities to COSMIC SBS40 (0.87 and 0.88) and 3 other Signal signatures (SBS18 (BER deficiency), SBS127 (not annotated) and SBS167 (annotated as tentative of lack of evidence of existence)), with cosine similarities between 0.83 and 0.85). Again, while cosine similarities are higher and visual comparison reveals similarities of some parts of the signatures, there are enough differences to provide uncertainty in the match, especially given that the cosine similarities of the top 2 matches are very similar (cosine similarity difference of 0.03 or less).

**Second round of review**

**Reviewer 1**

Thank you for addressing comments and providing candid response to my comments.

**Reviewer 2**

I thank the authors for their replies and edits. I have no more questions.

**Reviewer 3**

This revised manuscript has adequately addressed most suggestions. I have no further comments.
